# Supplementary material for: SARS-CoV-2 infection induces beta cell transdifferentiation
Source: Cell Metab. 2021 Aug 3;33(8):1577–1591.e7. doi: 10.1016/j.cmet.2021.05.015 (PMC8133495; doi:10.1016/j.cmet.2021.05.015)
Supplement: Document S1. Figures S1–S7 and Tables S1–S5 [file mmc1.pdf]

Cell Metabolism, Volume *33*

Supplemental information

SARS-CoV-2 infection induces

beta cell transdifferentiation

Xuming Tang, Skyler Uhl, Tuo Zhang, Dongxiang Xue, Bo Li, J. Jeya Vandana, Joshua A. Acklin, Lori L. Bonnycastle, Narisu Narisu, Michael R. Erdos, Yaron Bram, Vasuretha Chandar, Angie Chi Nok Chong, Lauretta A. Lacko, Zaw Min, Jean K. Lim, Alain C. Borczuk, Jenny Xiang, Ali Naji, Francis S. Collins, Todd Evans, Chengyang Liu, Benjamin R. tenOever, Robert E. Schwartz, and Shuibing Chen

SUPPLEMENTAL INFORMATION

Supplemental Figure S1-S7 and Tables S1-S5.

Figure S1

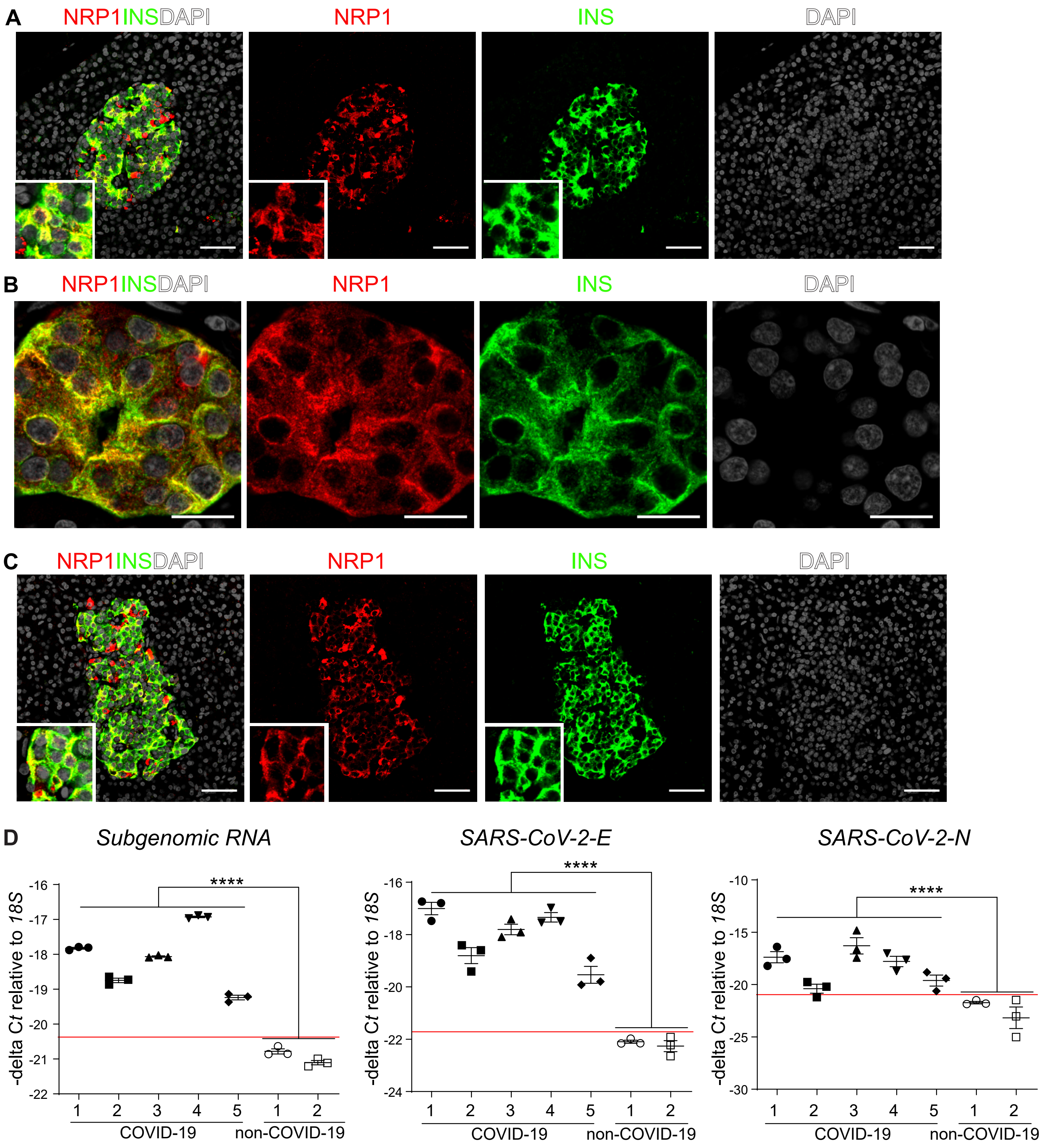
A
NRP1INSDAPI
NRP1
INS
DAPI
B
NRP1INSDAPI
NRP1
INS
DAPI
C
NRP1INSDAPI
NRP1
INS
DAPI
D
Subgenomic RNA
SARS-CoV-2-E
SARS-CoV-2-N
-delta Ct relative to 18S
****
COVID-19
non-COVID-19
1
2
3
4
5
1
2

SUPPLEMENTAL FIGURES.

Figure S1. Related to Figure 1 and 2. Immunostaining and qRT-PCR analysis of the autopsy pancreas samples of a non-COVID-19 and COVID-19 subjects.

(A) A representative image of NRP1 expression in the autopsy pancreas sample of a non-COVID-19 subject. 20X confocal image using NRP1 antibody from abcam. The insert represents a high-resolution image from the larger field. Scale bar = 50 µm. (*n* = 6 images examined in total) Red: NRP1; Green: INS; Grey: DAPI.

(B) A representative image of NRP1 in the autopsy pancreas sample of a non-COVID-19 subject. 63X confocal image using NRP1 antibody from NOVUS. Scale bar = 20 µm. (*n* = 2 images examined in total) Red: NRP1; Green: INS; Grey: DAPI.

(C) A representative image of NRP1 in the autopsy pancreas sample of a non-COVID-19 subject. 20X confocal image using NRP1 antibody from NOVUS. The insert represents a high-resolution image from the larger field. Scale bar = 50 µm. (*n* = 6 images examined in total) Red: NRP1; Green: INS; Grey: DAPI.

(D) qRT-PCR analysis of *subgenomic RNA*, *SARS-CoV-2-E*, *SARS-CoV-2-N* in the autopsy pancreas samples of non-COVID-19 and COVID-19 subjects. -Delta C*t* = C*t* (*18S*)-C*t* (*subgenomic RNA*, *SARS-CoV-2-E*, or *SARS-CoV-2-N*). Red line highlights the detection limit. Data was presented as mean ± STDEV. *P* values were calculated by one-way ANOVA, ****$P < 0.0001$.

Figure S2

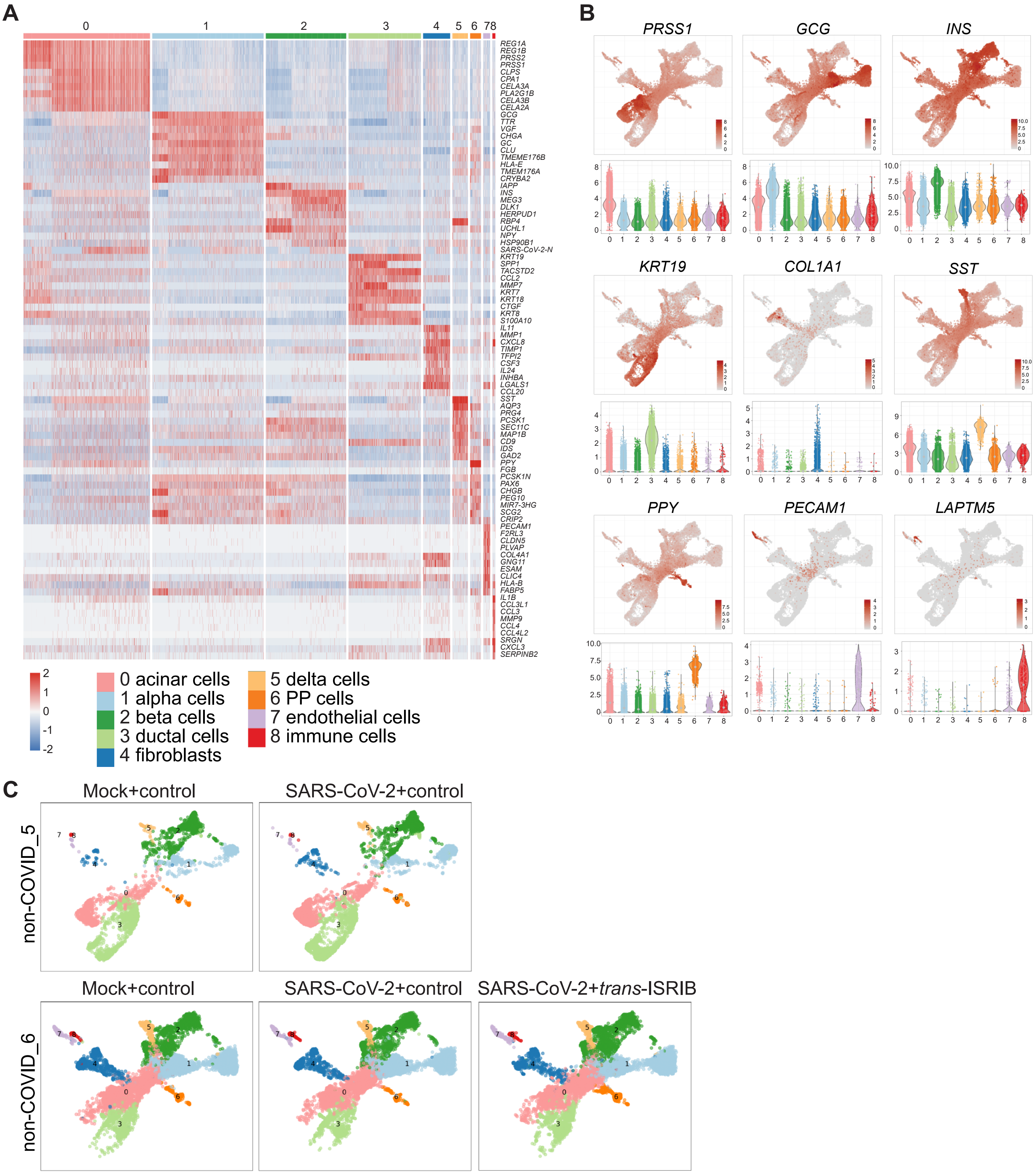
A
0
1
2
3
4
5
6
78
REG1A
REG1B
PRSS2
PRSS1
CLPS
CPA1
CELA3A
PLA2G1B
CELA3B
CELA2A
GCG
TTR
VGF
CHGA
GC
CLU
TMEME176B
HLA-E
TMEM176A
CRYBA2
IAPP
INS
MEG3
DLK1
HERPUD1
RBP4
UCHL1
NPY
HSP90B1
SARS-CoV-2-N
KRT19
SPP1
TACSTD2
CCL2
MMP7
KRT7
KRT18
CTGF
KRT8
S100A10
IL11
MMP1
CXCL8
TIMP1
TFPI2
CSF3
IL24
INHBA
LGALS1
CCL20
SST
AQP3
PRG4
PCSK1
SEC11C
MAP1B
CD9
IDS
GAD2
PPY
FGB
PCSK1N
PAX6
CHGB
PEG10
MIR7-3HG
SCG2
CRIP2
PECAM1
F2RL3
CLDN5
PLVAP
COL4A1
GNG11
ESAM
CLIC4
HLA-B
FABP5
IL1B
CCL3L1
CCL3
MMP9
CCL4
CCL4L2
SRGN
CXCL3
SERPINB2
2
1
0
-1
-2
0 acinar cells
1 alpha cells
2 beta cells
3 ductal cells
4 fibroblasts
5 delta cells
6 PP cells
7 endothelial cells
8 immune cells
B
PRSS1
GCG
INS
KRT19
COL1A1
SST
PPY
PECAM1
LAPTM5
C
Mock+control
SARS-CoV-2+control
SARS-CoV-2+trans-ISRIB
non-COVID_5
non-COVID_6

Figure S2. Related to Figure 2. Single-cell RNA-seq analysis of mock versus SARS-CoV-2 infected human islets.

(A) Heatmap showing relative expression of top 10 markers defining the nine cell types in human islets. (*n* = 2 individual islet donors).

(B) UMAP and violin plots showing expression levels of pancreatic cell markers. (*n* = 2 individual islet donors).

(C) UMAP of human islets by conditions.

Figure S3

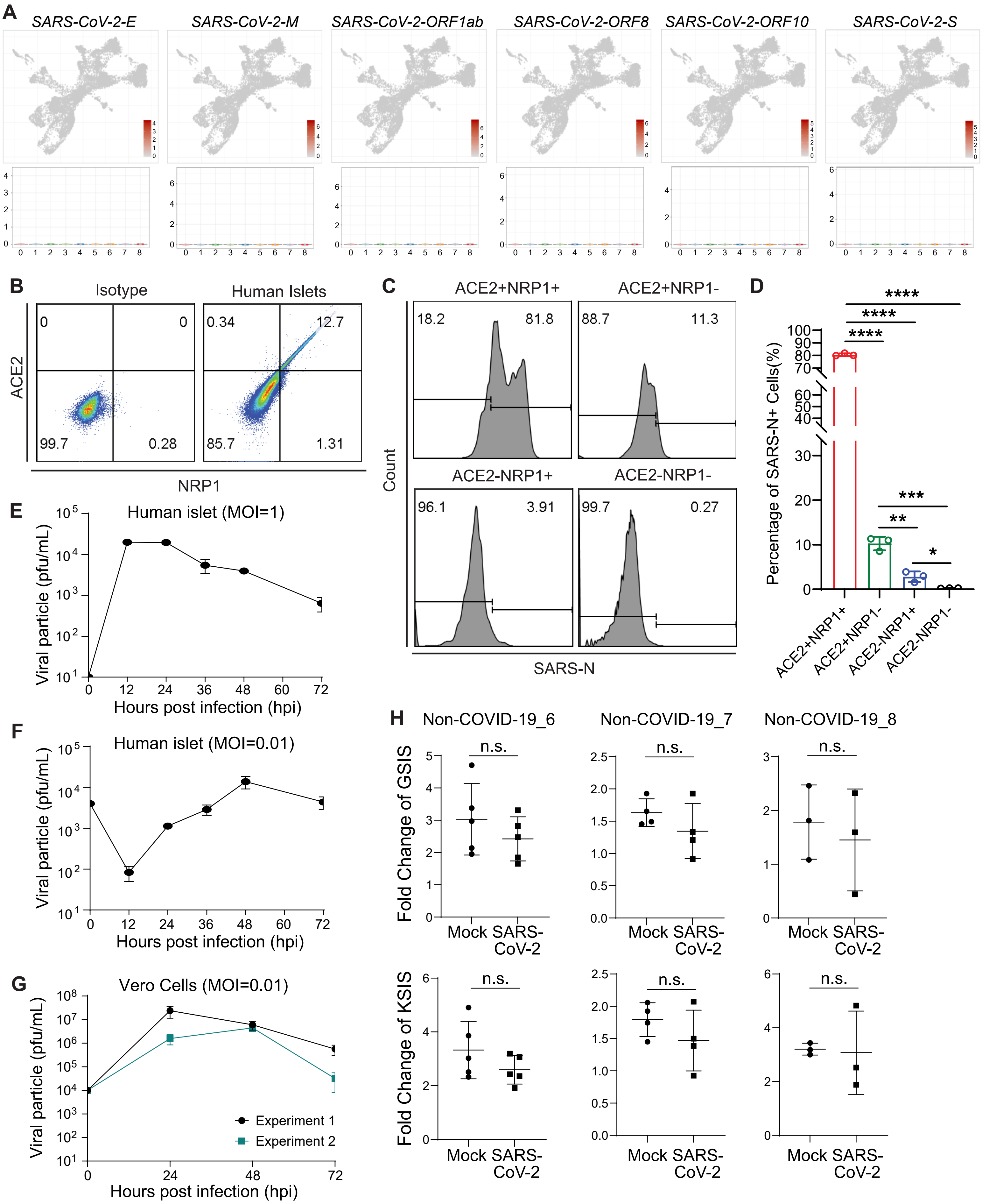
A
SARS-CoV-2-E
SARS-CoV-2-M
SARS-CoV-2-ORF1ab
SARS-CoV-2-ORF8
SARS-CoV-2-ORF10
SARS-CoV-2-S
B
Isotype
Human Islets
ACE2
NRP1
0
0
99.7
0.28
0.34
12.7
85.7
1.31
C
ACE2+NRP1+
ACE2+NRP1-
ACE2-NRP1+
ACE2-NRP1-
18.2
81.8
88.7
11.3
96.1
3.91
99.7
0.27
Count
SARS-N
D
Percentage of SARS-N+ Cells(%)
****
***
**
*
ACE2+NRP1+
ACE2+NRP1-
ACE2-NRP1+
ACE2-NRP1-
E
Human islet (MOI=1)
Viral particle (pfu/mL)
Hours post infection (hpi)
F
Human islet (MOI=0.01)
Viral particle (pfu/mL)
Hours post infection (hpi)
G
Vero Cells (MOI=0.01)
Viral particle (pfu/mL)
Experiment 1
Experiment 2
Hours post infection (hpi)
H
Non-COVID-19_6
Non-COVID-19_7
Non-COVID-19_8
Fold Change of GSIS
Fold Change of KSIS
n.s.
Mock
SARS-CoV-2

Figure S3. Related to Figure 2. SARS-CoV-2 infects multiple types of human pancreatic cells.
(A) UMAP and violin plots showing the expression levels of SARS-CoV-2 genes of mock infected human islets. (*n* = 2 individual islet donors).
(B and C) Flow cytometry analysis of SARS-CoV-2 (MOI=1) infected human islets at 48 hpi. Gating strategy of ACE2 and NRP1 (B). SARS-N expression in $ACE2^+/NRP1^+$, $ACE2^-/NRP1^+$, $ACE2^+/NRP1^-$, and $ACE2^-/NRP1^-$ cells (C). *n* = 3 replicates.
(D) Quantification of $SARS\text{-}N^+$ percentage in $ACE2^+/NRP1^+$, $ACE2^+/NRP1^-$, $ACE2^-/NRP1^+$, and $ACE2^-/NRP1^-$ cells.
(E) Plaque assays of SARS-CoV-2 (MOI=1) infected human islets at 12, 24, 36, 48, 60, and 72 hpi (*n* = 3 replicates per time point) with replacement of media and 3 times washes after initial 3 hr infection.
(F) Plaque assays of SARS-CoV-2 (MOI=0.01) infected human islets at 12, 24, 36, 48, 60 and 72 hpi (*n* = 3 replicates per time point).
(G) Plaque assays of SARS-CoV-2 (MOI=0.01) infected Vero cells at 24, 48, and 72 hpi (*n* = 3 replicates per time points from two individual experiments).
(H) Fold change of glucose stimulated insulin secretion (GSIS) or KCl stimulated insulin secretion (KSIS) of SARS-CoV-2 (MOI=1) infected human islets. Human islets from non-COVID-19_6 (*n* = 5 replicates per condition) and non-COVID-19_7 (*n* = 4 replicates per condition) were analyzed at 48 hpi. Human islets from non-COVID-19_8 (*n* = 3 replicates per condition) were analyzed at 72 hpi.
Data was presented as mean ± STDEV. *P* values were calculated by two-way ANOVA. $^*P < 0.05$, $^{**}P < 0.01$, $^{***}P < 0.001$ and $^{****}P < 0.0001$.

Figure S4

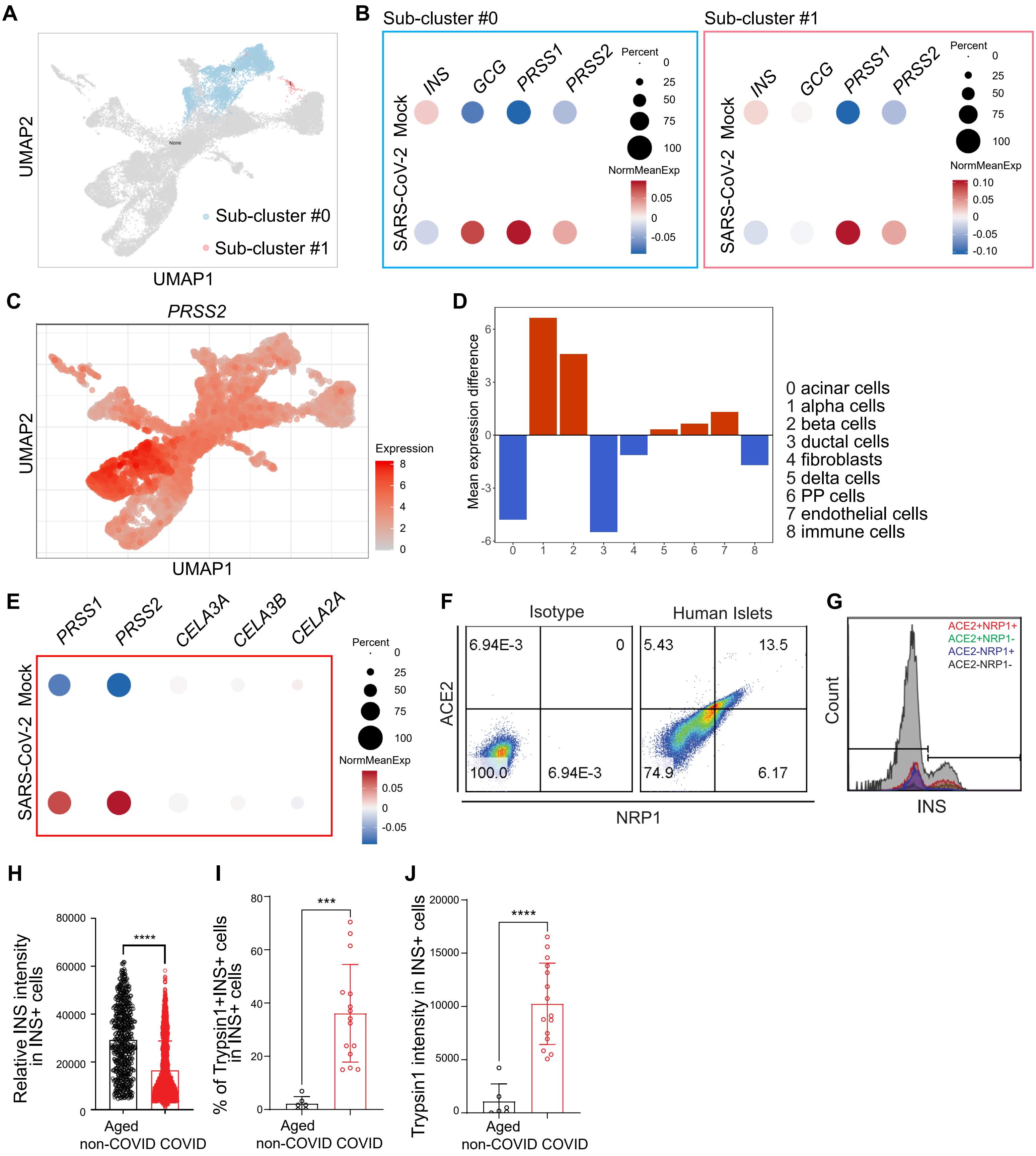

A
UMAP2
UMAP1
Sub-cluster #0
Sub-cluster #1
B
Sub-cluster #0
Sub-cluster #1
INS
GCG
PRSS1
PRSS2
Mock
SARS-CoV-2
Percent
0
25
50
75
100
NormMeanExp
0.10
0.05
0
-0.05
-0.10
C
PRSS2
UMAP2
UMAP1
Expression
8
6
4
2
0
D
Mean expression difference
0 acinar cells
1 alpha cells
2 beta cells
3 ductal cells
4 fibroblasts
5 delta cells
6 PP cells
7 endothelial cells
8 immune cells
E
PRSS1
PRSS2
CELA3A
CELA3B
CELA2A
Mock
SARS-CoV-2
F
Isotype
Human Islets
ACE2
NRP1
6.94E-3
0
100.0
6.94E-3
5.43
13.5
74.9
6.17
G
ACE2+NRP1+
ACE2+NRP1-
ACE2-NRP1+
ACE2-NRP1-
Count
INS
H
Relative INS intensity in INS+ cells
80000
60000
40000
20000
0
****
Aged non-COVID
COVID
I
% of Trypsin1+INS+ cells in INS+ cells
80
60
40
20
0
***
Aged non-COVID
COVID
J
Trypsin1 intensity in INS+ cells
20000
15000
10000
5000
0
****
Aged non-COVID
COVID

Figure S4. Related to Figure 4. Human beta cells undergo transdifferentiation upon SARS-CoV-2 infection.
(A) UMAP plot showing the two sub-clusters of beta cell population. (*n* = 2 individual islet donors).
(B) Dot blot illustrating expression level of *INS*, *GCG, PRSS1, PRSS2* in two sub-clusters of mock versus SARS-CoV-2-infected human islets at 24 hpi (MOI=1). Dot size shows the fraction of cells with non-zero expression; Dot color indicates the relative expression level in the two conditions. (*n* = 2 individual islet donors).
(C) UMAP plot showing the expression level of *PRSS2* of SARS-CoV-2 infected human islets. (*n* = 2 individual islet donors).
(D) Bar plot illustrating mean expression difference of *PRSS2* between mock and SARS-CoV-2 infected cells in different clusters of human islets; Bar color indicates increased (red) or decreased (blue) expression in the SARS-CoV-2 infected cells. (*n* = 2 individual islet donors).
(E) Dot blot illustrating expression level of acinar cell markers, including *PRSS1, PRSS2, CELA3A, CELA3B, CELA2A* in beta cell cluster of mock versus SARS-CoV-2-infected human islets at 24 hpi (MOI=1). Dot size shows the fraction of cells with non-zero expression; Dot color indicates the relative expression level in the two conditions. (*n* = 2 individual islet donors).
(F and G) Flow cytometry analysis of SARS-CoV-2 (MOI=1) infected human islets at 48 hpi. Gating strategy of ACE2 and NRP1 (F). Insulin expression in $ACE2^+/NRP1^+$, $ACE2^-/NRP1^+$, $ACE2^+/NRP1^-$, and $ACE2^-/NRP1^-$ cells (G). (*n* = 3 replicates).
(H-J) Quantification of the relative INS intensity in INS^+ cells (H), the percentage of $Typsin1^+INS^+$ cells in INS^+ cells (I) and the average Typsin1 intensity in INS^+ cells (J) of autopsy samples of COVID-19 subjects or age-matched non-COVID-19 subjects. Three images of each sample were used for quantification for each subject (*n* = 2 individual non-COVID-19 subjects; *n* = 5 individual COVID-19 subjects).
Data was presented as mean ± STDEV. *P* values were calculated by paired or unpaired two-tailed Student's t test. ***$P < 0.001$ and ****$P < 0.0001$.

Figure S5

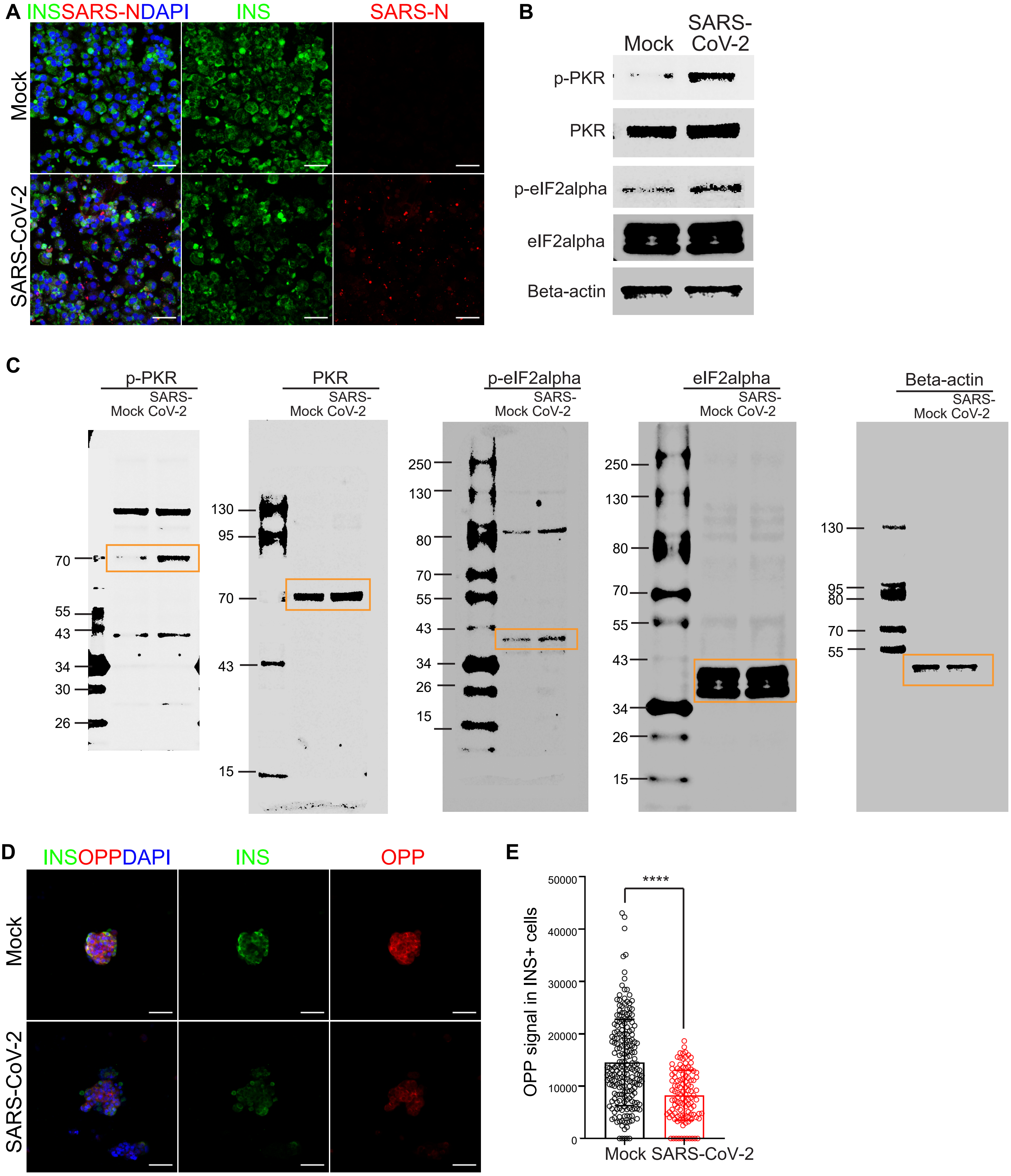
A
INSSARS-NDAPI
INS
SARS-N
Mock
SARS-CoV-2
B
Mock
SARS-CoV-2
p-PKR
PKR
p-eIF2alpha
eIF2alpha
Beta-actin
C
p-PKR
SARS-
Mock CoV-2
70
55
43
34
30
26
PKR
130
95
70
43
15
p-eIF2alpha
250
130
80
70
55
43
34
26
15
eIF2alpha
250
130
80
70
55
43
34
26
15
Beta-actin
130
95
80
70
55
D
INSOPPDAPI
INS
OPP
Mock
SARS-CoV-2
E
OPP signal in INS+ cells
50000
40000
30000
20000
10000
0
****
Mock
SARS-CoV-2

Figure S5. Related to Figure 4. SARS-CoV-2 infection causes the change of eIF2 pathway and the decrease of protein synthesis.
(A) Representative confocal images of INS and SARS-N of mock and SARS-CoV-2 (MOI=1) infected EndoC-betaH1 cells at 48 hpi (MOI=1). Scale bar = 50 μm. Red: SARS-N; Green: INS; Blue: DAPI.
(B) Western blotting of p-PKR, PKR, p-eIF2alpha, eIF2alpha and beta-actin of mock and SARS-CoV-2 (MOI=1) infected EndoC-betaH1 cells at 48 hpi (MOI=1).
(C) Whole gel images of western blotting at (B).
(D and E) Representative confocal images of INS and OPP (O-propargyl-puromycin) (D) and quantification of OPP staining intensity (E) of mock versus SARS-CoV-2 infected human islets at 48 hpi (*n* = 3 replicates, MOI=1). Scale bar = 50 μm. Red: OPP; Green: INS; Blue: DAPI.

Figure S6

A

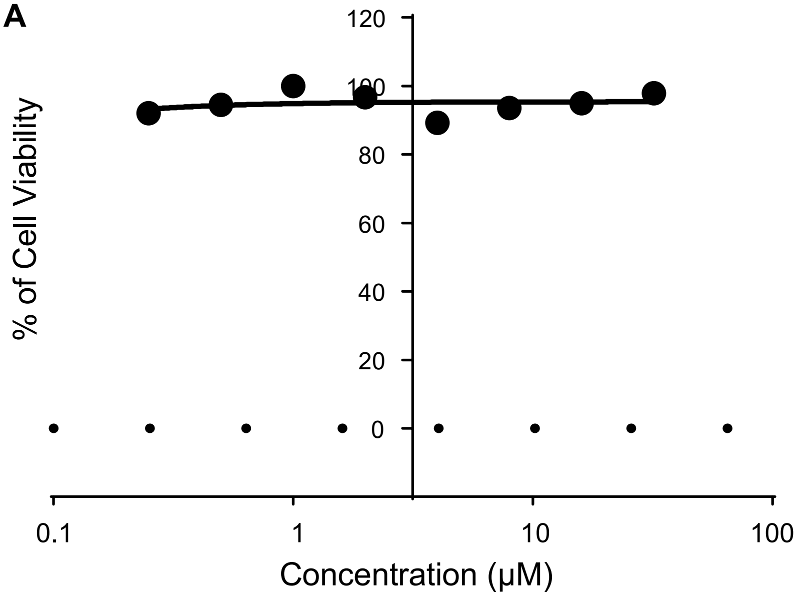

B

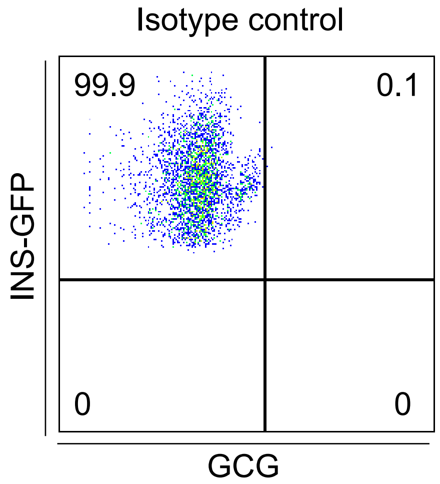

C

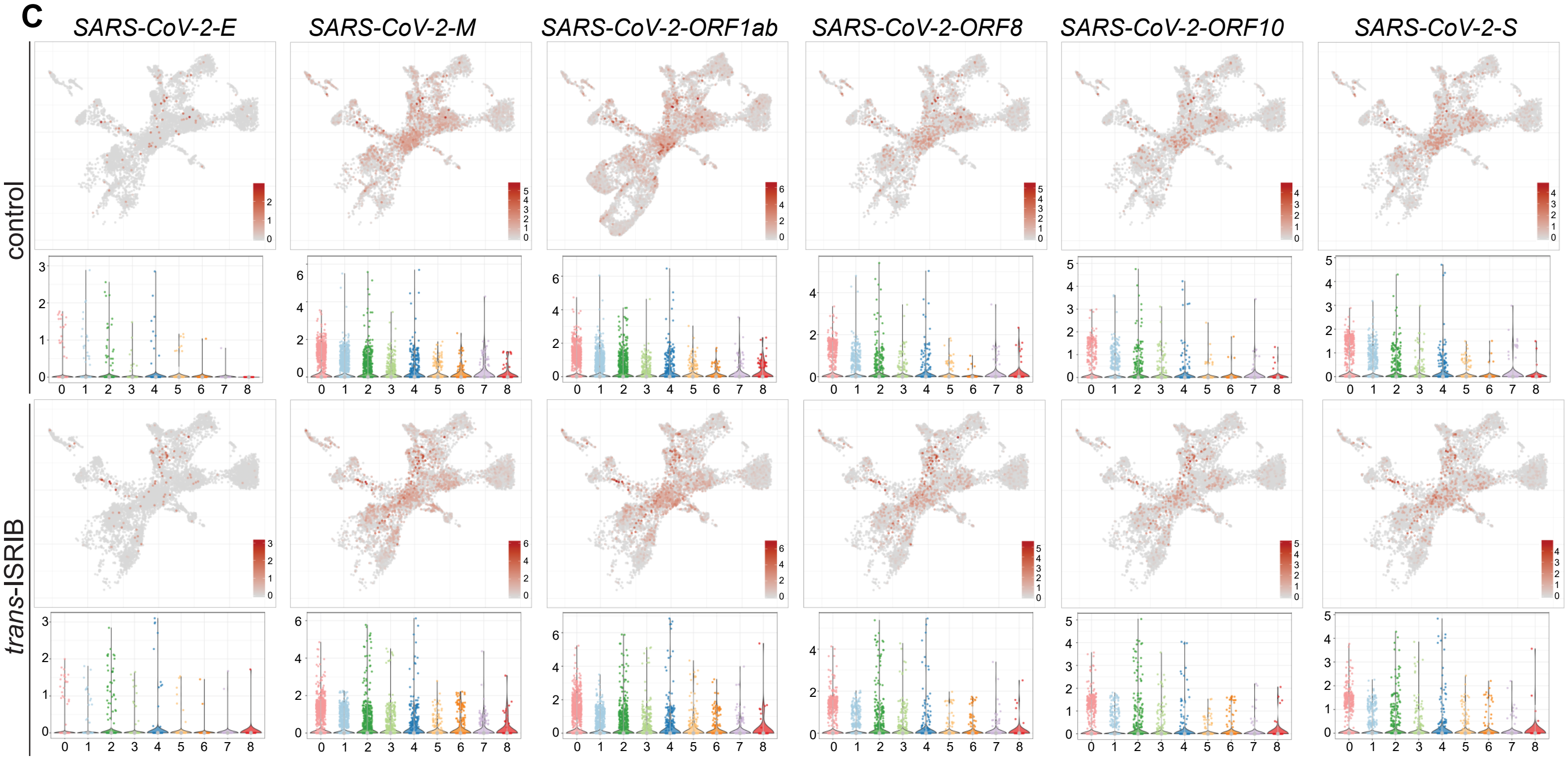

Figure S6. Related to Figure 6 and 7. High-throughput chemical screen.
(A) Toxicity curve of *trans*-ISRIB on hESC-derived *INS*-GFP$^+$ beta-like cells. Data was presented as mean ± STDEV. (n = 3 biological replicates).
(B) Isotype control of flow cytometry analysis of *trans*-ISRIB treated hESC-derived *INS*-GFP$^+$ beta-like cells.
(C) UMAP and violin plots showing the expression levels of SARS-CoV-2 genes of control or 10 µM *trans*-ISRIB treated human islets at 24 hpi (n = 2 individual islet donors, MOI=1).

Figure S7

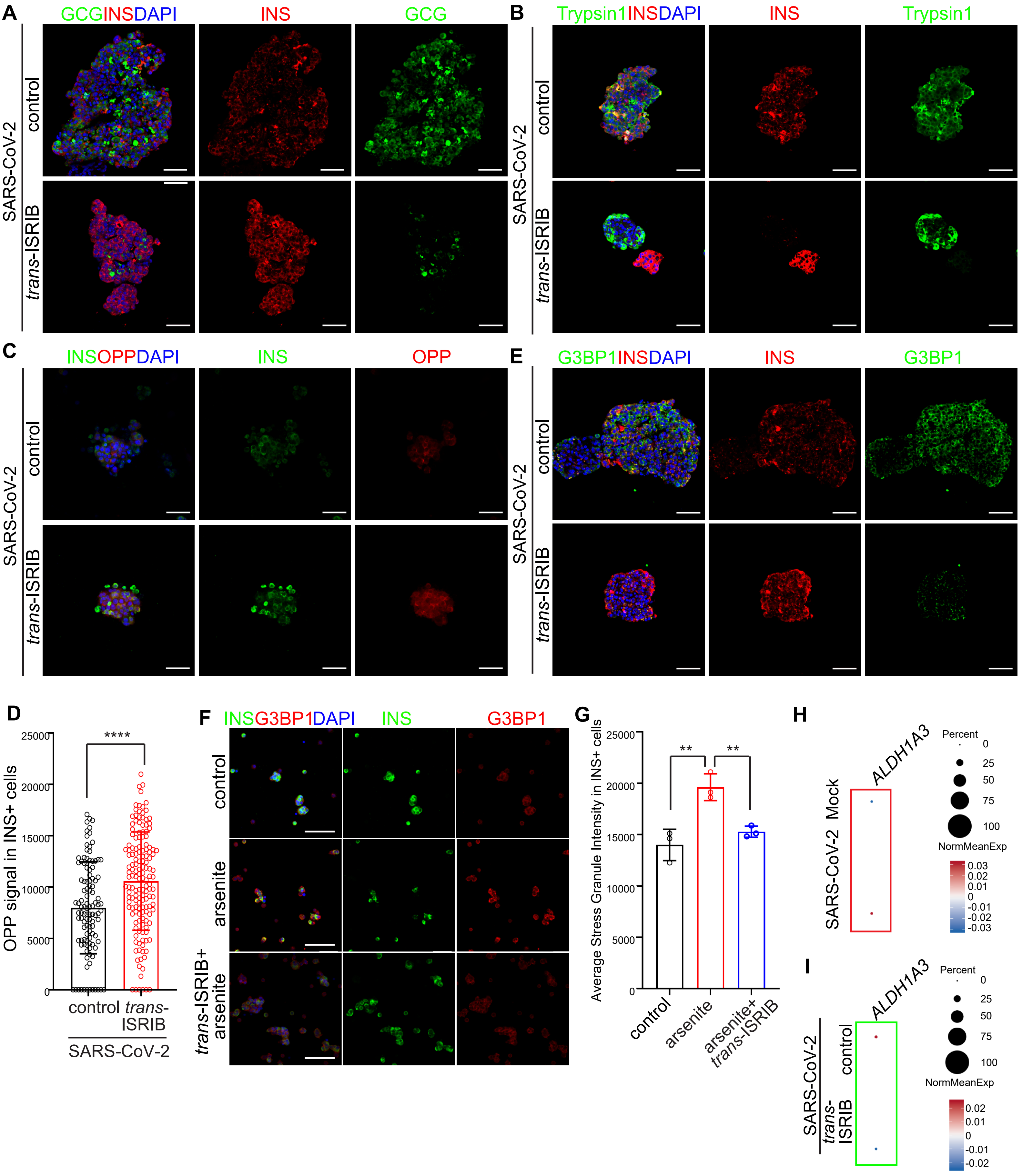
A
GCGINSDAPI
INS
GCG
SARS-CoV-2
control
trans-ISRIB
B
Trypsin1INSDAPI
INS
Trypsin1
SARS-CoV-2
control
trans-ISRIB
C
INSOPPDAPI
INS
OPP
SARS-CoV-2
control
trans-ISRIB
E
G3BP1INSDAPI
INS
G3BP1
SARS-CoV-2
control
trans-ISRIB
D
OPP signal in INS+ cells
25000
20000
15000
10000
5000
0
****
control
trans-ISRIB
SARS-CoV-2
F
INSG3BP1DAPI
INS
G3BP1
control
arsenite
trans-ISRIB+ arsenite
G
Average Stress Granule Intensity in INS+ cells
25000
20000
15000
10000
5000
0
**
**
control
arsenite
arsenite+ trans-ISRIB
H
ALDH1A3
SARS-CoV-2 Mock
Percent
0
25
50
75
100
NormMeanExp
0.03
0.02
0.01
0
-0.01
-0.02
-0.03
I
ALDH1A3
SARS-CoV-2
control
trans-ISRIB
Percent
0
25
50
75
100
NormMeanExp
0.02
0.01
0
-0.01
-0.02

Figure S7. Related to Figure 7. *Trans*-ISRIB blocks human beta cells transdifferentiation upon SARS-CoV-2 infection.

(A) Representative confocal images of GCG and INS expression in control or 10 μM *trans*-ISRIB treated human islets at 48 hpi (n = 3 individual islet donors, MOI=1). Scale bar = 50 μm. Red: INS; Green: GCG; Blue: DAPI.

(B) Representative confocal images of Typsin1 and INS expression in control or 10 μM *trans*-ISRIB treated human islets at 48 hpi (n = 3 individual islet donors, MOI=1). Scale bar = 50 μm. Red: INS; Green: Typsin1; Blue: DAPI.

(C and D) Representative confocal images of INS and OPP (C) and quantification of OPP staining intensity (D) of control or 10 μM *trans*-ISRIB treated human islets at 48 hpi (n = 3 replicates, MOI=1). Scale bar = 50 μm. Red: OPP; Green: INS; Blue: DAPI.

(E) Representative confocal images of G3BP1 and INS expression in control or 10 μM *trans*-ISRIB treated human islets at 48 hpi (n = 3 individual islet donors, MOI=1). Scale bar = 50 μm. Red: INS; Green: G3BP1; Blue: DAPI.

(F and G) Representative confocal images of G3BP1 and INS (F) and quantification of G3BP1 expression (G) in control, 0.5 mM $NaAsO_2$ and 10 μM *trans*-ISRIB+ 0.5 mM $NaAsO_2$ treated human islets after 30 min treatment (n = 3 replicates). Scale bar = 50 μm. Green: INS; Red: G3BP1; Blue: DAPI.

(H) Dot blot illustrating expression level of *ALDH1A3* in mock versus SARS-CoV-2 infected human islets at 24 hpi (n = 2 individual islet donors, MOI=1).

(I) Dot blot illustrating expression level of *ALDH1A3* in control or 10 μM *trans*-ISRIB treated human islets at 24 hpi (n = 2 individual islet donors, MOI=1).

Dot size shows the fraction of cells with non-zero expression; Dot color indicates the relative expression level in the two conditions.

Data was presented as mean ± STDEV. P values were calculated by paired or unpaired two-tailed Student's t test. $^{**}P < 0.01$ and $^{****}P < 0.0001$.

Table S1. Related to Figure 1. Summary of samples.

Sample ID	Gender	Age	Assays	Chronic comorbiditi es	Inflammat ory status WBC	COVID-19 disease course	Blood glucose level (mg/dL)
COVID-19_1	Female	87	Immunostaining	DM2, HTN	17.4	7	248
COVID-19_2	Female	70	Immunostaining	Dementia, DM2	4.9	12	223
COVID-19_3	Male	87	Immunostaining	None	9.7	26	213
COVID-19_4	Male	91	Immunostaining	Dementia, HTN	6.8	5	229
COVID-19_5	Female	71	Immunostaining	Alzheimer's, HTN, AFIB	4.2	7	100
Non-COVID-19_1	Male	75	Immunostaining	HTN, CAD	18.9	N/A	201
Non-COVID-19_2	Female	74	Immunostaining	CAD, mild HTN	9.2	N/A	190
Non-COVID-19_3	Male	19	Immunostaining	Asthma	20.4	N/A	150
Non-COVID-19_4	Male	27	Immunostaining	Ashama, HTN, OB	21.6	N/A	137
Non-COVID-19_5	Male	27	SARS-CoV-2 infection, scRNA-seq and immunostaining	Allergic rhinitis, pharyngitis	7.7	N/A	129
Non-COVID-19_6	Female	58	SARS-CoV-2 infection, scRNA-seq and immunostaining, ELISA	HTN, Stroke	12.4	N/A	144
Non-COVID-19_7	Male	52	SARS-CoV-2 infection and immunostaining, ELISA	HTN	7.1	N/A	104
Non-COVID-19_8	Male	18	SARS-CoV-2 infection, immunostaining, ELISA	N/A	N/A	N/A	N/A
Non-COVID-19_15	Male	50	scRNA-seq (Table S3)	N/A	N/A	N/A	N/A
Non-COVID-19_16	Male	28	scRNA-seq (Table S3)	N/A	N/A	N/A	N/A
Non-COVID-19_17	Male	35	scRNA-seq (Table S3)	N/A	N/A	N/A	N/A

Non-COVID-19_18	Male	22	scRNA-seq (Table S3)	N/A	N/A	N/A	N/A
Non-COVID-19_19	Female	41	scRNA-seq (Table S3)	N/A	N/A	N/A	N/A
Non-COVID-19 (T2D)_20	Male	61	scRNA-seq (Table S3)	N/A	N/A	N/A	N/A

DM2: diabetes mellitus, type 2; HTN: Hypertension; AFIB: Atrial fibrillation; CAD: Coronary Artery Disease; OB: obesity.

Table S2. Related to Figure 1. The SARS-N$^+$ cells in autopsy samples*.

Sample ID	COVID-19_1	COVID-19_2	COVID-19_3	COVID-19_4	COVID-19_5
# of SARS-N$^+$INS$^+$ cells	6.5±2.0	9.7±4.7	14.5±4.8	8.0±7.0	23.6±7.5
% of SARS-N$^+$INS$^+$ cells in INS$^+$ cells	10.0±10.9%	15.7±15.3%	7.7±4.1%	8.1±5.9%	12.0±9.1%
# of SARS-N$^+$ KRT19$^+$ cells	22.0±18.5	4.9±5.8	8.3±8.5	3.3±3.2	8.0±13.9
% of SARS-N$^+$ KRT19$^+$ cells in KRT19$^+$ cells	9.0±6.8%	2.1±2.4%	7.8±6.6%	1.9±1.4%	3.0±5.1%
# of SARS-N$^+$ CD31$^+$ cells	29.3±6.0	34.1±22.6	3.0±3.0	9.3±3.5	1.6±1.5
% of SARS-N$^+$ CD31$^+$ cells in CD31$^+$ cells	21.0±0.9%	27.4±13.1%	4.1±4.8%	11.8±4.2%	2.3±2.3%
# of SARS-N$^+$ VIM$^+$ cells	84.7±68.7	5.3±1.5	10.4±4.8	4.2±2.4	7.7±4.0
% of SARS-N$^+$ VIM$^+$cells in VIM $^+$ cells	16.3±10.8%	2.4±1.5%	2.6±1.0%	4.1±1.9%	4.1±0.4%
# of SARS-N$^+$ Trypsin1$^+$ cells	133.7±111.4	4.0±1.0	21.6±15.9	18.2±4.4	5.7±4.5
% of SARS-N$^+$ Trypsin1$^+$ cells in Trypsin1$^+$ cells	15.4±11.0%	0.8±0.0%	2.9±2.1%	2.2±0.5%	1.2±1.0%

*The staining was only performed using autopsy samples that contain viral transcripts as confirmed by qRT-PCR.

Table S3. Related to Figure 2. The percentage of *FURIN*⁺ or *CTSL*⁺ cells in six human islet samples.

	**Percentage of *FURIN*$^+$ cells** (Mean±STDEV)	**Percentage of *CTSL*$^+$ cells** (Mean±STDEV)
acinar cells	27.3±17.5	25.9±19.0
alpha cells	23.5±13.8	51.5±22.9
beta cells	31.1±19.0	51.4±30.2
delta cells	33.1±12.1	62.7±22.7
PP cells	36.6±26.0	48.5±28.7
ductal cells	42.8±17.9	51.3±24.6
fibroblasts	47.2±19.4	76.1±18.5
endothelial cells	32.8±32.8	47.3±28.6
immune cells	29.9±17.8	90.5±11.5

Table S4. Related to STAR Methods. Antibodies used for immunocytochemistry, intracellular flow cytometry analysis.

Usage	Antibody	Clone#	Host	Catalog #	Vendor	Dilution
Immuno-staining	Polyclonal Guinea Pig Anti-Insulin	Polyclonal	Guinea Pig	#A0564	Dako	1:500
Immuno-staining	Anti-Glucagon antibody	Monoclonal	Mouse	#ab10988	Abcam	1:1,000
Immuno-staining	Human/Mouse Somatostatin Antibody	Monoclonal	Rat	#MAB2358	R&D Systems	1:100
Immuno-staining	Pancreatic Polypeptide/PP Antibody	Polyclonal	Goat	#NB100-1793	NOVUS Biologicals	1:200
Immuno-staining	Human Serpin A1/ alpha 1-Antitrypsin Antibody	Monoclonal	Mouse	#MAB1268	R&D Systems	1:200
Immuno-staining	Trypsin1/PRSS1 Antibody	Polyclonal	Sheep	#AF3848	NOVUS Biologicals	1:2,500
Immuno-staining	Anti-Cytokeratin 19 antibody	Monoclonal	Mouse	# ab7754	Abcam	1:200
Immuno-staining	Human CD31/PECAM-1 Antibody	Polyclonal	Sheep	#AF806	R&D Systems	1:200
Immuno-staining	Vimentin antibody	Monoclonal	Mouse	#ab8978	Abcam	1:200
Immuno-staining	NRP1 antibody	Monoclonal	Rabbit	#ab81321	Abcam	1:200
Immuno-staining	NRP1 antibody	Monoclonal	Rabbit	#ST05-30	NOVUS Biologicals	1:200
Immuno-staining	G3BP1 Antibody	Polyclonal	Rabbit	#17798	Cell Signaling Technology	1:500
Immuno-staining	SARS-CoV/SARS-CoV-2 Nucleocapsid Antibody	Monoclonal	Rabbit	#40143-R001	Sino Biological	1:500
Immuno-staining	Human ACE-2 Antibody	Polyclonal	Goat	#AF933	R & D Systems	1:400
Immuno-staining	Alexa Fluor 488 AffiniPure Donkey Anti-Guinea Pig IgG (H+L)	Polyclonal	Donkey	#706-545-148	Jackson ImmunoResearch Labs	1:1,000
Immuno-staining	Donkey anti-Mouse IgG (H+L) Highly Cross-Adsorbed Secondary Antibody, Alexa Fluor 488	Polyclonal	Donkey	#A-21202	Thermo Fisher Scientific	1:1,000

Immuno-staining	Donkey anti-Mouse IgG (H+L) Highly Cross-Adsorbed Secondary Antibody, Alexa Fluor 594	Polyclonal	Donkey	#A-21203	Thermo Fisher Scientific	1:1,000
Immuno-staining	Donkey anti-Mouse IgG (H+L) Highly Cross-Adsorbed Secondary Antibody, Alexa Fluor 647	Polyclonal	Donkey	#A-31571	Thermo Fisher Scientific	1:1,000
Immuno-staining	Donkey anti-Rabbit IgG (H+L) Secondary Antibody, Alexa Fluor 594 conjugate	Polyclonal	Donkey	#A-21207	Thermo Fisher Scientific	1:1,000
Immuno-staining	Donkey anti-Rabbit IgG (H+L) Secondary Antibody, Alexa Fluor 647 conjugate	Polyclonal	Donkey	#A-31573	Thermo Fisher Scientific	1:1,000
Immuno-staining	Donkey anti-Goat IgG (H+L) Cross-Adsorbed Secondary Antibody, Alexa Fluor 488	Polyclonal	Donkey	#A-11055	Thermo Fisher Scientific	1:1,000
Immuno-staining	Donkey anti-Rat IgG (H+L) Highly Cross-Adsorbed Secondary Antibody, Alexa Fluor 488	Polyclonal	Donkey	#A-21208	Thermo Fisher Scientific	1:1,000
Immuno-staining	Donkey anti-Sheep IgG (H+L) Cross-Adsorbed Secondary Antibody, Alexa Fluor 488	Polyclonal	Donkey	#A-11015	Thermo Fisher Scientific	1:1,000
Flow Cytometry	Polyclonal Guinea Pig Anti-Insulin	Polyclonal	Guinea Pig	#A0564	Dako	1:500
Flow Cytometry	Anti-Glucagon antibody	Monoclonal	Mouse	#ab10988	Abcam	1:1,000
Flow Cytometry	Neuropilin-1 antibody	Polyclonal	Sheep	#AF3870	NOVUS Biologicals	1:200
Flow Cytometry	Alexa Fluor 488 AffiniPure Donkey Anti-Guinea Pig IgG (H+L)	Polyclonal	Donkey	#706-545-148	Jackson ImmunoResearch Labs	1:1,000
Flow Cytometry	Donkey anti-Mouse IgG (H+L) Highly Cross-Adsorbed Secondary Antibody, Alexa Fluor 647	Polyclonal	Donkey	#A-31571	Thermo Fisher Scientific	1:1,000

Flow Cytometry	Goat anti-Rabbit IgG (H+L) Cross-Adsorbed Secondary Antibody, Pacific Blue	Polyclonal	Goat	#P-10994	Thermo Fisher Scientific	1:1,000
Western Blot	eIF2alpha Rabbit mAb	Monoclonal	Rabbit	#5324S	Cell Signaling	1:1,000
Western Blot	Phospho-eIF2alpha (Ser51) Rabbit mAb	Monoclonal	Rabbit	#3398S	Cell Signaling	1:1,000
Western Blot	PKR Rabbit mAb	Monoclonal	Rabbit	#12297S	Cell Signaling	1:1,000
Western Blot	β-Actin Rabbit mAb #8457	Monoclonal	Rabbit	#8457S	Cell Signaling	1:1,000
Western Blot	Recombinant Anti-PKR (phospho T446) antibody	Monoclonal	Rabbit	# ab32036	Abcam	1:1,000
Western Blot	IRDye® 800CW Donkey anti-Rabbit IgG Secondary Antibody	Polyclonal	Donkey	#926-32213	LI-COR	1:15,000

Table S5. Related to STAR Methods. Primers used for qRT-PCR.

Primer name	Sequence
ACTB-human-F	*CGTCACCAACTGGGACGACA*
ACTB- human-R	*CTTCTCGCGGTTGGCCTTGG*
SARS-CoV-2-TRS-F	*CTCTTGTAGATCTGTTCTCTAAACGAAC*
SARS-CoV-2-TRS-R	*GGTCCACCAAACGTAATGCG*
SARS-CoV-2-N-R	*TAATCAGACAAGGAACTGATTA*
SARS-CoV-2-N-R	*CGAAGGTGTGACTTCCATG*
SARS-CoV-2-E-F	*ACAGGTACGTTAATAGTTAATAGCGT*
SARS-CoV-2-E-R	*ATATTGCAGCAGTACGCACACA*
18S-F	*GGCCCTGTAATTGGAATGAGTC*
18S-R	*CCAAGATCCAACTACGAGCTT*
