# Supplementary material for: SARS-CoV-2 infection induces beta cell transdifferentiation
Source: Cell Metab. 2021 Aug 3;33(8):1577–1591.e7. doi: 10.1016/j.cmet.2021.05.015 (PMC8133495; doi:10.1016/j.cmet.2021.05.015)
Supplement: Document S3. Article plus supplemental information [file mmc4.pdf]

Cell Metabolism
Clinical and Translational Report

SARS-CoV-2 infection induces beta cell transdifferentiation

Graphical abstract

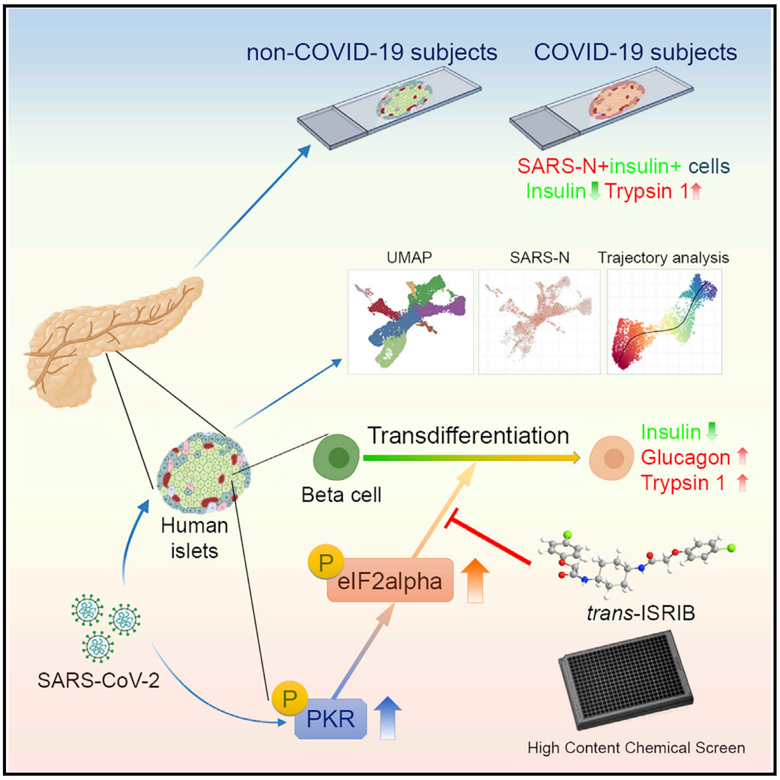

Authors

Xuming Tang, Skyler Uhl, Tuo Zhang, ..., Benjamin R. tenOever, Robert E. Schwartz, Shuibing Chen

Correspondence

chliu@pennmedicine.upenn.edu (C.L.),
benjamin.tenoever@mssm.edu (B.R.t.),
rec2025@med.cornell.edu (R.E.S.),
shc2034@med.cornell.edu (S.C.)

In brief

Here, Tang et al. reported the detection of SARS-CoV-2 viral antigen in autopsy samples from COVID-19 subjects. In addition, SARS-CoV-2 infection induces eIF2-pathway-mediated beta cell transdifferentiation, a phenotype that can be reversed by *trans*-ISRIB.

Highlights

- SARS-CoV-2 viral antigen is detected in beta cells of autopsies of COVID-19 subjects
- SARS-CoV-2 infection causes beta cell transdifferentiation
- SARS-CoV-2-induced beta cell transdifferentiation is mediated by eIF2 pathway
- *Trans*-ISRIB reverses SARS-CoV-2 infection-induced beta cell transdifferentiation

Tang et al., 2021, Cell Metabolism *33*, 1577–1591
August 3, 2021 © 2021 The Author(s). Published by Elsevier Inc.
https://doi.org/10.1016/j.cmet.2021.05.015

CellPress

Cell Metabolism

CellPress
OPEN ACCESS

Clinical and Translational Report

SARS-CoV-2 infection induces beta cell transdifferentiation

Xuming Tang,[1,11] Skyler Uhl,[2,3,11] Tuo Zhang,[4,11] Dongxiang Xue,[1,11] Bo Li,[1] J. Jeya Vandana,[1,5] Joshua A. Acklin,[2,3] Lori L. Bonnycastle,[6] Narisu Narisu,[6] Michael R. Erdos,[6] Yaron Bram,[7] Vasuretha Chandar,[7] Angie Chi Nok Chong,[1] Lauretta A. Lacko,[1] Zaw Min,[8] Jean K. Lim,[2] Alain C. Borczuk,[9] Jenny Xiang,[4] Ali Naji,[8] Francis S. Collins,[6] Todd Evans,[1] Chengyang Liu,[8,*] Benjamin R. tenOever,[2,*] Robert E. Schwartz,[7,10,*] and Shuibing Chen[1,12,*]

[1]Department of Surgery, Weill Cornell Medicine, 1300 York Avenue, New York, NY 10065, USA
[2]Department of Microbiology, Icahn School of Medicine at Mount Sinai, 1468 Madison Avenue, New York, NY 10029, USA
[3]Graduate School of Biomedical Sciences, Icahn School of Medicine at Mount Sinai, 1468 Madison Avenue, New York, NY 10029, USA
[4]Genomics Resources Core Facility, Weill Cornell Medicine, 1300 York Avenue, New York, NY 10065, USA
[5]Tri-Institutional PhD Program in Chemical Biology, Weill Cornell Medicine, the Rockefeller University, Memorial Sloan Kettering Cancer Center, New York, NY 10065, USA
[6]The Center for Precision Health Research, National Human Genome Research Institute, National Institutes of Health, 9000 Rockville Pike, Bethesda, MD 20892, USA
[7]Division of Gastroenterology and Hepatology, Department of Medicine, Weill Cornell Medicine, 1300 York Avenue, New York, NY 10065, USA
[8]Department of Surgery, University of Pennsylvania School of Medicine, Philadelphia, PA 19104, USA
[9]Department of Pathology and Laboratory Medicine, Weill Cornell Medicine, 1300 York Avenue, New York, NY 10065, USA
[10]Department of Physiology, Biophysics and Systems Biology, Weill Cornell Medicine, 1300 York Avenue, New York, NY 10065, USA
[11]These authors contributed equally
[12]Lead contact
*Correspondence: chliu@pennmedicine.upenn.edu (C.L.), benjamin.tenoever@mssm.edu (B.R.t.), rec2025@med.cornell.edu (R.E.S.), shc2034@med.cornell.edu (S.C.)
https://doi.org/10.1016/j.cmet.2021.05.015

SUMMARY

Recent clinical data have suggested a correlation between coronavirus disease 2019 (COVID-19) and diabetes. Here, we describe the detection of SARS-CoV-2 viral antigen in pancreatic beta cells in autopsy samples from individuals with COVID-19. Single-cell RNA sequencing and immunostaining from *ex vivo* infections confirmed that multiple types of pancreatic islet cells were susceptible to SARS-CoV-2, eliciting a cellular stress response and the induction of chemokines. Upon SARS-CoV-2 infection, beta cells showed a lower expression of insulin and a higher expression of alpha and acinar cell markers, including glucagon and trypsin1, respectively, suggesting cellular transdifferentiation. Trajectory analysis indicated that SARS-CoV-2 induced eIF2-pathway-mediated beta cell transdifferentiation, a phenotype that could be reversed with *trans*-integrated stress response inhibitor (*trans*-ISRIB). Altogether, this study demonstrates an example of SARS-CoV-2 infection causing cell fate change, which provides further insight into the pathomechanisms of COVID-19.

INTRODUCTION

Coronavirus disease 2019 (COVID-19) encompasses a myriad of pathologies caused by severe acute respiratory syndrome coronavirus 2 (SARS-CoV-2). Although respiratory failure is the most common clinical feature, many individuals with COVID-19 present with additional clinical complications, including cardiac defects, gastrointestinal symptoms, kidney damage, abnormal liver function, neurological manifestations, and a number of metabolic phenotypes. Clinical studies have also suggested a close interaction between COVID-19 and diabetes. Individuals with diabetes and severe obesity are more likely to be symptomatic, are at a higher risk for complications, and have a higher COVID-19 mortality rate (Cummings et al., 2020; Zhou et al., 2020; Zhu et al., 2020). Conversely, new-onset diabetes and severe metabolic complications of pre-existing diabetes have also been observed in individuals with COVID-19. Recently, several cases have been reported involving the new onset of diabetes or diabetic ketoacidosis following SARS-CoV-2 infection (Chee et al., 2020; Heaney et al., 2020; Hollstein et al., 2020; Liang et al., 2021; Soliman et al., 2020; Unsworth et al., 2020). In addition, subjects with newly diagnosed diabetes have been reported to have significantly higher rates of admittance to intensive care unit (Li et al., 2020), in-hospital complications, and death (Fadini et al., 2020; Wang et al., 2020; Yang et al., 2020a).

© 2021 The Author(s). Published by Elsevier Inc.
This is an open access article under the CC BY license (http://creativecommons.org/licenses/by/4.0/).

SARS-CoV-2 is an enveloped, non-segmented, positive-strand RNA virus. Several host factors have been shown to be involved in SARS-CoV-2 viral entry, including angiotensin-converting enzyme 2 (ACE2) (Hoffmann et al., 2020), the entry factor neurophilin-1 (Cantuti-Castelvetri et al., 2020; Daly et al., 2020), the two proteinases TMPRSS2 (Hoffmann et al., 2020) and CTSL (Ou et al., 2020), and the pro-protein convertase FURIN (Shang et al., 2020), among others (Daniloski et al., 2021; Schneider et al., 2021; Wang et al., 2021; Wei et al., 2021). Using single-cell RNA sequencing (scRNA-seq), *ACE2* expression has been detected in a wide variety of cell types, including ciliated and secretory cells in the nasal cavity; basal, ciliated, and secretory cells in lung bronchi; alveolar epithelial type 2 (AT2) cells in lung parenchyma; basal corneal epithelium, limbal niche, corneal wing cells, limbal superficial cells, superficial conjunctiva, and corneal epithelial superficial cells in the cornea; and enterocytes in ileum, enterocytes, and goblet cells (Ashary et al., 2020; Sungnak et al., 2020; Ziegler et al., 2020; Zou et al., 2020). However, overall *ACE2* expression in these tissues based on scRNA-seq analyses is low. As an example, the percentage of $ACE2^{+}$ cells in lung AT2 cells, one of the major target cells of SARS-CoV-2, varies between 0.3% and 2.4% depending on the subjects (Hou et al., 2020; Ortiz Bezara et al., 2020; Qi et al., 2020), which might reflect the low detection sensitivity of scRNA-seq platforms, as suggested elsewhere (Hou et al., 2020). Several groups have reported the detection of *ACE2* and *TMPRSS2* in human islet cells, particularly in beta cells, via scRNA-seq (Liu et al., 2020; Yang et al., 2020b). Consistent with our finding, Coate et al. reported <1.5% $ACE2^{+}$ cells in human islets based on scRNA-seq (Coate et al., 2020). In addition, *TMPRSS2* is also expressed at low levels in human endocrine cells. However, TMPRSS2 is not required for SARS-CoV-2 entry. This is exemplified by the fact that Vero E6 cells, which lack *TMPRSS2* expression, are a commonly used cell line to propagate SARS-CoV-2. Thus, the expression of *TMPRSS2* alone is not sufficient to predict SARS-CoV-2 permissiveness.

Previously, we reported that beta cells of primary human islets can be infected by SARS-CoV-2 (Yang et al., 2020b). Additional studies confirmed that SARS-CoV-2 could infect and replicate in cells of the human endocrine pancreas (Müller et al., 2021). Here, we directly validated the detection of SARS-CoV-2 viral antigen in pancreatic beta cells using autopsy samples of COVID-19 subjects. In addition, we applied scRNA-seq to analyze SARS-CoV-2-infected human islets. scRNA-seq has been broadly used to study heterogeneity within human pancreatic islets in both healthy (Baron et al., 2016; Li et al., 2016; Muraro et al., 2016; Wang et al., 2016) and disease states, such as type 2 diabetes (Segerstolpe et al., 2016; Xin et al., 2016) and aging (Enge et al., 2017). Moreover, scRNA-seq has also been applied to study the molecular mechanisms controlling pancreatic beta cell development, maturation (Qiu et al., 2017), proliferation (Zeng et al., 2017), insulin production (Xin et al., 2018), and intra-species differences (Baron et al., 2016).

Here, we applied scRNA-seq analysis of SARS-CoV-2-infected human islets and showed that most types of endocrine cells can be infected. Upon SARS-CoV-2 infection, beta cells transdifferentiate, which leads to lower insulin (*INS*) expression and higher production of glucagon (*GCG*) and trypsin1 (*PRSS1*). Combining a trajectory analysis and the results of a high-throughput chemical screen, we found that SARS-CoV-2-induced beta cell transdifferentiation is mediated by the eIF2 pathway. In summary, we dissected the host pancreatic cellular response upon SARS-CoV-2 infection at the single-cell level and provide an example of cell fate change caused by SARS-CoV-2.

RESULTS AND DISCUSSION

Detection of SARS-CoV-2 viral antigen in human endocrine cells of autopsy samples of COVID-19 subjects

Recently, two studies reported neuropilin-1 as a host factor that facilitates SARS-CoV-2 infection (Cantuti-Castelvetri et al., 2020; Daly et al., 2020). We stained autopsy pancreatic tissues from non-COVID-19 subjects using two different antibodies and found that neuropilin-1 is highly expressed in human islets, in particular human beta cells (Figures 1A and S1A–S1C; Video S1). We also inspected the autopsy samples of COVID-19 subjects to determine whether SARS-CoV-2 viral antigen is detectable in human islet cells. qRT-PCR confirmed the presence of viral transcripts, including *SARS-CoV-2-E* and *SARS-CoV-2-N*, as well as a low amount of replicating viral RNA, as indicated by the presence of subgenomic RNA (Figure S1D). SARS-CoV-2 nucleocapsid $(SARS\text{-}N)^{+}$ cells were detected in the autopsy samples of COVID-19 subjects, but not in non-COVID-19 subjects. Co-staining with pancreatic endocrine and non-endocrine markers confirmed the detection of SARS-N in INS^{+}E-Cadherin $(E\text{-}Cad)^{+}$ beta cells, $CD31^{+}$ endothelial cells, keratin 19 $(KRT19)^{+}$ ductal cells, $Trypsin1^{+}$ acinar cells, and vimentin $(VIM)^{+}$ mesenchymal cells (Figures 1B–1D; Tables S1 and S2), suggesting that both endocrine and non-endocrine cells in the pancreas can harbor SARS-CoV-2 viral antigen. High-resolution confocal images (Figure 1E) and 3D reconstruction of the confocal images (Video S2) further validated the SARS-N staining in INS^{+} cells.

scRNA-seq validates the permissiveness of human endocrine cells to SARS-CoV-2 infection

To determine the response of human islets to SARS-CoV-2 infection, we infected primary human islets *ex vivo* with SARS-CoV-2 (MOI = 1). Cells were collected at 24 h post infection (hpi) and analyzed by scRNA-seq. Clustering analysis confirmed the presence of $PRSS1^{+}$ acinar cells, GCG^{+} alpha cells, INS^{+} beta cells, $KRT19^{+}$ ductal cells, $COL1A1^{+}$ fibroblasts, PPY^{+} PP cells, SST^{+} delta cells, $PECAM1^{+}$ endothelial cells, and $LAPTM5^{+}$ immune cells (Figures 2A and S2). Here, we assessed the expression of other proteinases known to be involved in the entry of SARS-CoV-2, including *FURIN* (Shang et al., 2020) and *CTSL* (Ou et al., 2020). We found that *FURIN* and *CTSL* are expressed in all types of pancreatic cells (Figure 2B; Table S3). SARS-CoV-2 viral RNAs, including *SARS-CoV-2-E*, *SARS-CoV-2-M*, *SARS-CoV-2-ORF1ab*, *SARS-CoV-2-ORF8*, *SARS-CoV-2-ORF10*, and *SARS-CoV-2-S*, were highly expressed in acinar cells, alpha cells, beta cells, ductal cells, and fibroblasts and were expressed at relatively a low level in PP cells, delta cells, endothelial cells, and immune cells in the SARS-CoV-2-infected condition, but not in the mock-infected condition (Figures 2C and S3A). Immunostaining confirmed the presence of SARS-N protein in INS^{+} beta cells (38.7% ± 4.9%), GCG^{+} alpha cells (16.7% ± 14.4%), SST^{+} delta cells (34.0% ± 5.7%), PPY^{+} PP cells (44.1% ±

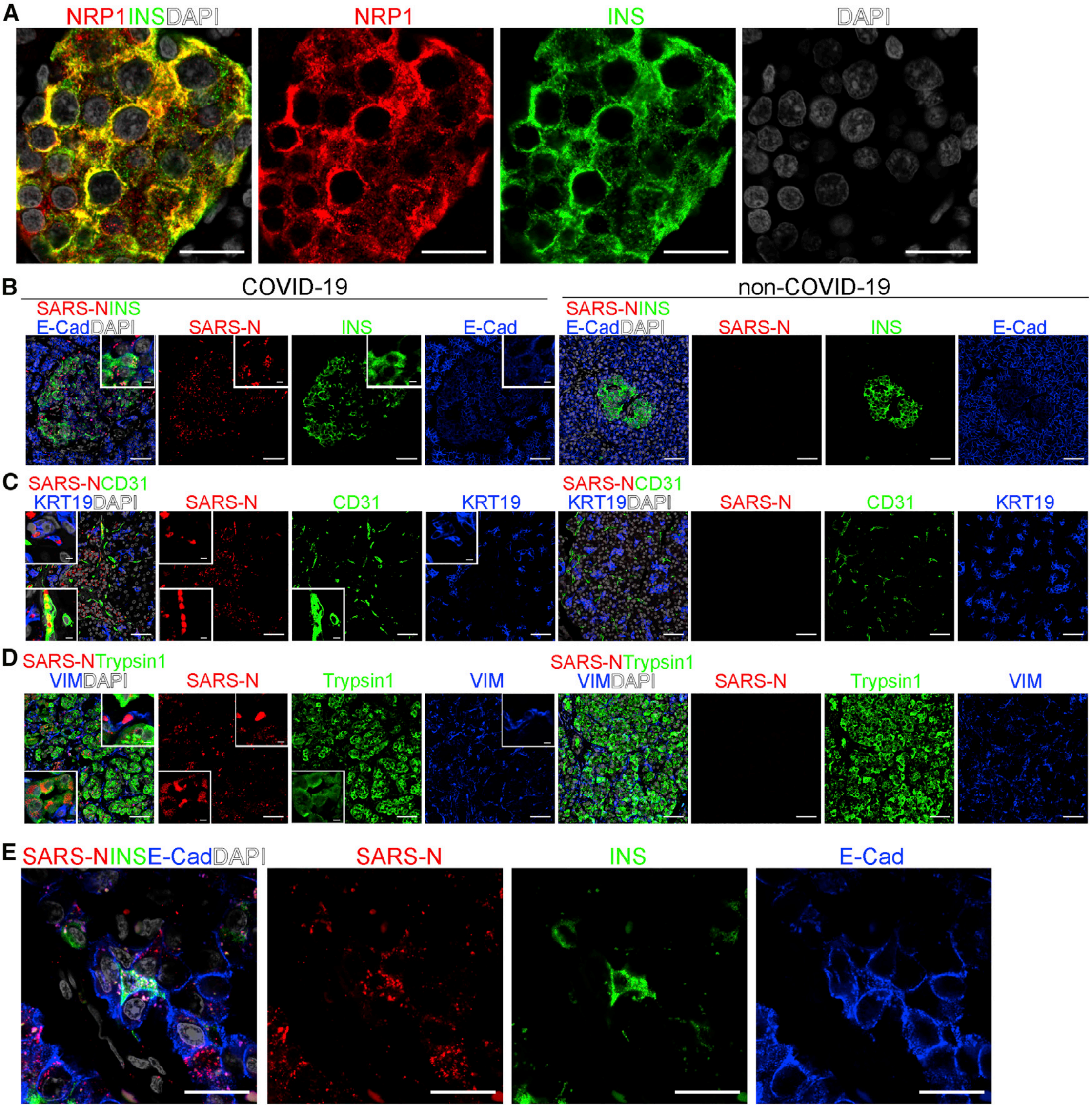

Figure 1. SARS-CoV-2 viral antigen is detected in beta cells and other pancreatic cells of COVID-19 subjects

(A) A representative 63× confocal image of NRP1 (Abcam) in the autopsy pancreas sample of a non-COVID-19 subject (n = 2 images examined in total). Scale bar, 20 μm. Red, NRP1; green, INS; gray, DAPI.

(B) Representative confocal images of INS, E-Cad, and SARS-N in the autopsy pancreas sample of non-COVID-19 and COVID-19 subjects (n = 2 images examined in non-COVID-19, n = 5 images examined in COVID-19). The inserts represent high-resolution images from the larger field. Scale bar, 50 μm. Scale bar of insert, 12 μm. Red, SARS-N; green, INS; blue, E-Cad; gray, DAPI.

(C) Representative confocal images of CD31, KRT19, and SARS-N in the autopsy pancreas samples of non-COVID-19 and COVID-19 subjects (n = 2 images examined in non-COVID-19, n = 5 images examined in COVID-19). The inserts represent high-resolution images from the larger field. Scale bar, 50 μm. Scale bar of insert, 12 μm. Red, SARS-N; green, CD31; blue, KRT19; gray, DAPI.

(D) Representative confocal images of trypsin1, VIM, and SARS-N in the autopsy pancreas samples of non-COVID-19 and COVID-19 subjects (n = 2 images examined in non-COVID-19, n = 5 images examined in COVID-19). The inserts represent high-resolution images from the larger field. Scale bar, 50 μm. Scale bar of insert, 12 μm. Red, SARS-N; green, trypsin1; blue, VIM; gray, DAPI.

(E) A representative 63× confocal image of INS, E-Cad, and SARS-N in autopsy pancreas sample of a COVID-19 subject (n = 5 images examined in total). Scale bar, 20 μm. Red, SARS-N; green, INS; blue, E-Cad; gray, DAPI.

INS, insulin; NRP1, neuropilin 1; E-Cad, E-cadherin; KRT19, keratin 19; VIM, vimentin; SARS-N, SARS-CoV-2 nucleocapsid.

19.6%), AAT^+ acinar cells (5.1% ± 2.2%), VIM^+ fibroblasts (16.6% ± 2.2%), and $CD31^+$ endothelial cells (3.3% ± 3.1%; Figure 2D). The percentage of SARS-N^+ cells was significantly higher in $ACE2^+NRP1^+$ cells than $ACE2^+NRP1^-$ cells, $ACE2^-NRP1^+$ cells, or $ACE2^-NRP1^-$ cells (Figures S3B–S3D). Plaque assay further validated the production of infectious viral particles at 12, 24, 36, 48, 60, and 72 hpi at MOI = 1 (Figure S3E) and MOI = 0.01 (Figures S3F and S3G), suggesting that in the absence of inflammation, the pancreas represented a potential site of productive virus infection if exposed.

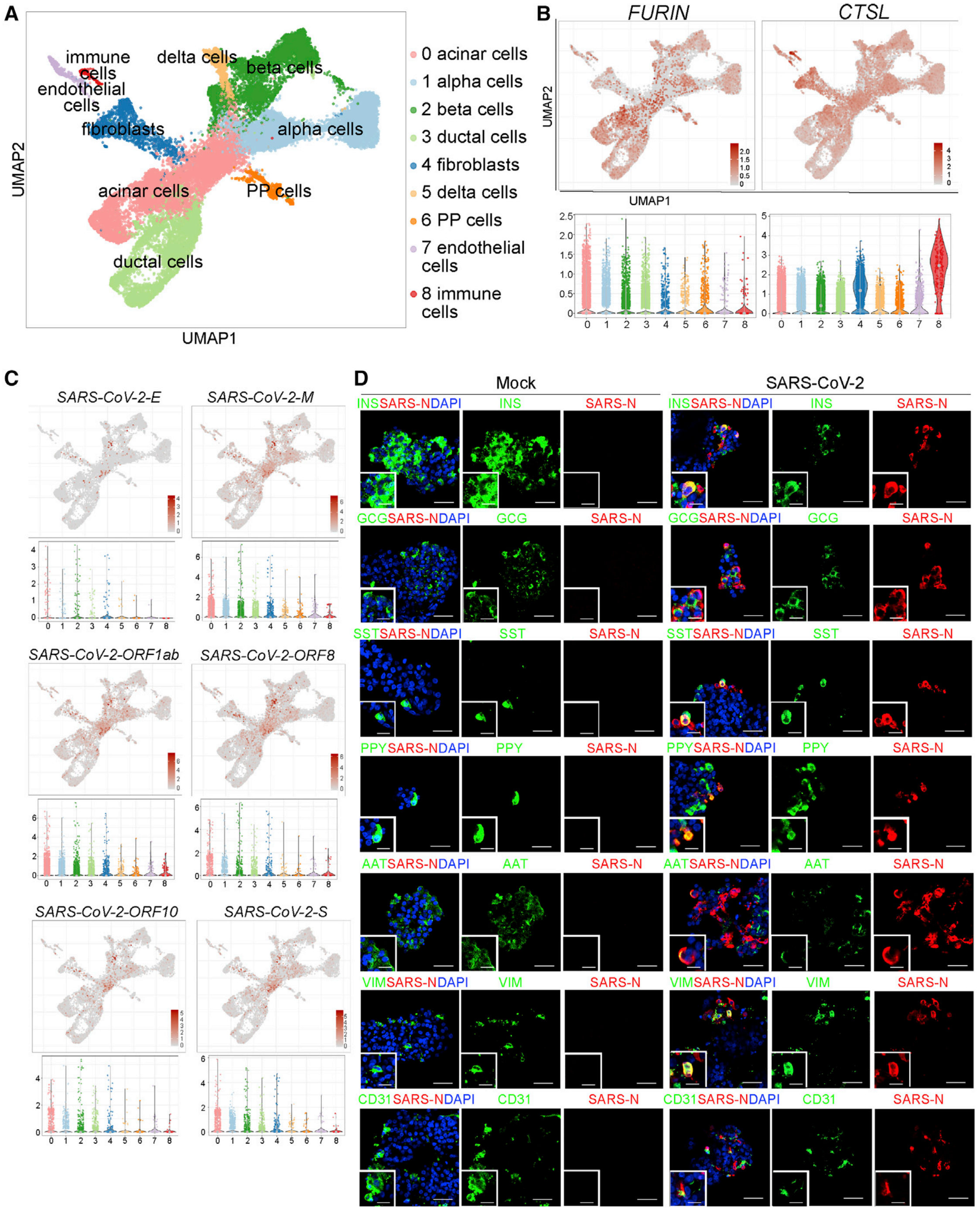

Figure 2. scRNA-seq analysis of mock- and SARS-CoV-2-infected human islets

(A) UMAP of human islets (n = 2 individual islet donors).

(B) UMAP and violin plots showing the expression levels of SARS-CoV-2 entry factors, including *FURIN* and *CTSL* (n = 2 individual islet donors).

(C) UMAP and violin plots showing the expression levels of SARS-CoV-2 genes, including *SARS-CoV-2-E*, *SARS-CoV-2-M*, *SARS-CoV-2-ORF1ab*, *SARS-CoV-2-ORF8*, *SARS-CoV-2-ORF10*, and *SARS-CoV-2-S* (n = 2 individual islet donors).

(D) Representative confocal images of INS, GCG, SST, PPY, AAT, VIM, CD31, and SARS-N of mock- and SARS-CoV-2- (MOI = 1) infected human islets at 48 hpi. The insert represents a high-resolution image from the larger field. Scale bar, 50 μm. Scale bar of insert, 25 μm. Red, SARS-N; green, INS, GCG, SST, PPY, AAT, VIM, and CD31; blue, DAPI (n = 3 individual islet donors).

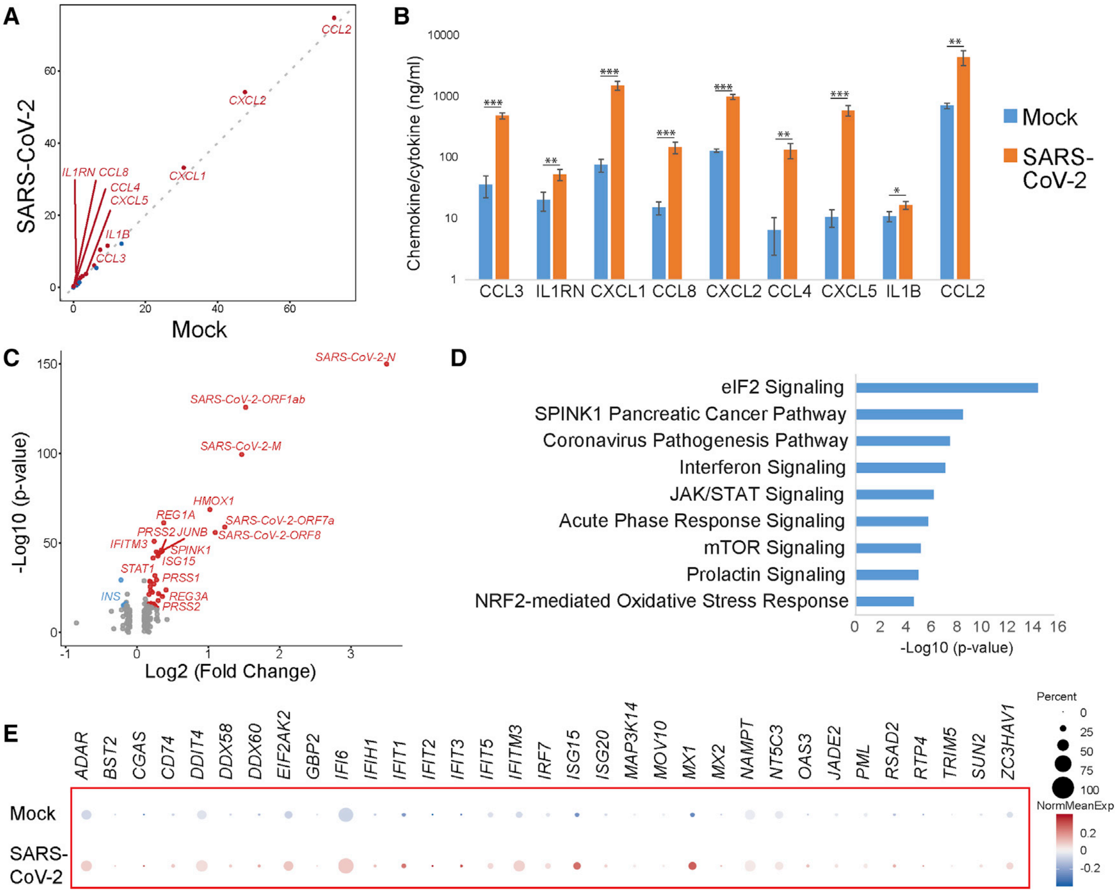

Figure 3. Human islets show upregulated chemokine response, cell stress, and interferon signaling upon SARS-CoV-2 infection

(A) Scoring the chemokine and cytokine expression levels in mock- versus SARS-CoV-2-infected human islets at 24 hpi. Higher score indicates higher expression level and more cells expressing a gene. The red dots indicate the genes encoding upregulated chemokines and cytokines. The blue dots indicate the genes encoding downregulated chemokines and cytokines (n = 2 individual islet donors).

(B) ELISA analysis of chemokine and cytokine expression in mock- versus SARS-CoV-2-infected human islets at 24 hpi (n = 3 replicates). Data are presented as mean ± SD. p values were calculated by paired two-tailed Student's t test. $^{*}p < 0.05$, $^{**}p < 0.01$, and $^{***}p < 0.001$.

(C) Volcano plot highlighting genes differentially expressed in mock- versus SARS-CoV-2-infected whole human islets at 24 hpi (n = 2 individual islet donors).

(D) Ingenuity pathway analysis of genes differentially expressed in mock- versus SARS-CoV-2-infected human islets at 24 hpi (n = 2 individual islet donors).

(E) Dot blot illustrating gene expression levels involved in interferon signaling pathway in mock- versus SARS-CoV-2-infected human islets at 24 hpi. Dot size shows the fraction of cells with non-zero expression; dot color indicates the relative expression level in the two conditions (n = 2 individual islet donors).

SARS-CoV-2 infection results in higher chemokine and cytokine expression and cell stress signals

We next compared the gene expression profiles in SARS-CoV-2- versus mock-infected human islets. Expression levels of multiple chemokines and cytokines, including *CCL2*, *CXCL2*, *CXCL1*, *CCL4*, *CCL3*, *CXCL5*, *CCL8*, *IL1RN*, and *IL1B*, were higher in SARS-CoV-2-infected condition than those of mock-infected condition (Figure 3A), which is consistent with previous reports in lung autopsy samples of COVID-19 subjects (Blanco-Melo et al., 2020) and organoid models (Han et al., 2021). ELISA analysis further validated the upregulation of chemokines and cytokines encoded by these genes (Figure 3B). Volcano plot analysis comparing the genes differentially expressed in SARS-CoV-2- versus mock-infected conditions further validated the presence of SARS-CoV-2 transcripts (Figure 3C). Ingenuity pathway analysis of the genes enriched in SARS-CoV-2-infected human islets highlighted canonical biological pathways associated with virus infection, including the cellular stress response, eukaryotic translation initiation factor 2 (eIF2) signaling pathway, as well as interferon and JAK-STAT signaling (Figure 3D). Dot blots further validated the upregulation of interferon stimulated genes in SARS-CoV-2-infected human islets (Figure 3E). We also monitored the function of the human islets upon SARS-CoV-2 infection. Due to the high variation, we did not detect statistically significant changes in insulin secretion upon glucose or KCl stimulation (Figure S3H).

Human pancreatic beta cells undergo transdifferentiation upon SARS-CoV-2 infection

To determine the response of beta cells to SARS-CoV-2 infection, we analyzed the scRNA-seq data to examine the expression level of *INS* in the beta cell cluster of mock- versus SARS-CoV-2-infected human islets. A dot blot suggested that the average *INS* transcriptional expression level is lower in beta cells upon SARS-CoV-2 infection than that of mock

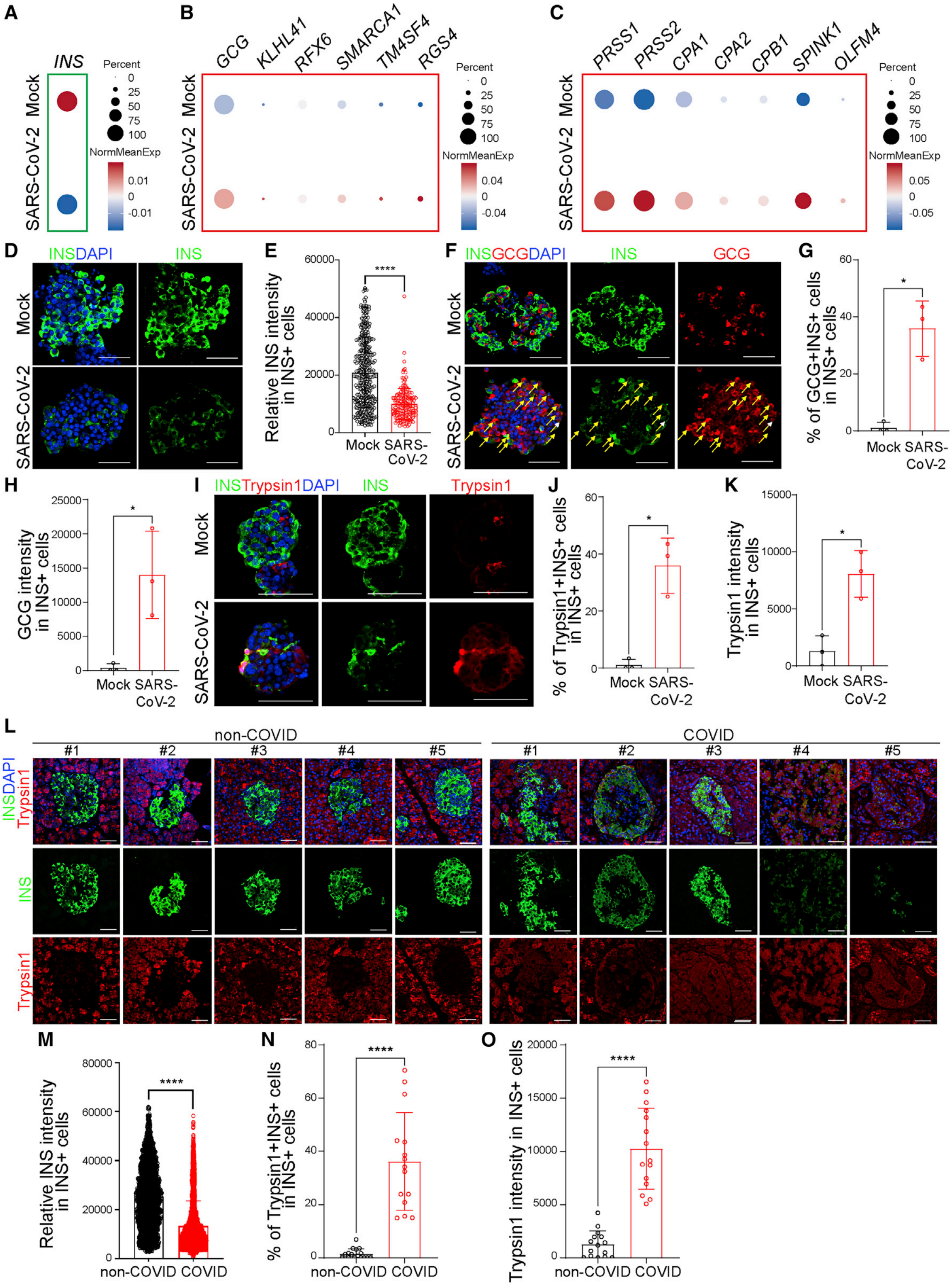

Figure 4. Human beta cells undergo transdifferentiation upon SARS-CoV-2 infection

(A–C) Dot blot illustrating expression level of *INS* (A); alpha cell markers, including *GCG*, *KLHL41*, *RFX6*, *SMARCA1*, *TM4SF4*, and *RGS4* (B); and acinar cell markers, including *PRSS1*, *PRSS2*, *CPA1*, *CPA2*, *CPB1*, *SPINK1*, and *OLFM4* (C), in mock- versus SARS-CoV-2-infected human islets at 24 hpi (MOI = 1). Dot size shows the fraction of cells with non-zero expression; dot color indicates the relative expression level in the two conditions (n = 2 individual islet donors).

(legend continued on next page)

condition (Figure 4A). Interestingly, the average transcriptional expression levels of alpha cell markers, such as *GCG*, *KLHL41*, *RFX6*, *SMARCA1*, *TM4SF4*, and *RGS4* (Figure 4B), and acinar cell markers, such as *PRSS1*, *PRSS2*, *CPA1*, *CPA2*, *CPB1*, *SPINK1*, and *OLFM4* (Figure 4C), were upregulated in beta cells after SARS-CoV-2 infection. The beta cells were further separated as sub-clusters 0 and 1, in which sub-cluster 1 showed relatively higher *GCG* expression (Figure S4A). In both beta cell sub-clusters, the expression of *INS* was lower and the expression of *GCG*, *PRSS1*, and *PRSS2* was higher in SARS-CoV-2-infected condition than those in mock condition, suggesting that the relative change of *INS*, *GCG*, *PRSS1*, and *PRSS2* expression is independent of beta cell sub-clusters (Figure S4B). We further examined the change of *PRSS2* in different cell clusters in SARS-CoV-2-infected islets (Figure S4C). The expression of *PRSS2* was higher in SARS-CoV-2-infected condition than mock condition only in alpha and beta cell clusters (Figure S4D). The expression of some other acinar cell markers, such as *CELA3A*, *CELA3B*, and *CELA2A*, was not significantly changed in SARS-CoV-2-infected beta cells (Figure S4E).

Immunostaining confirmed the lower level of INS expression in beta cells upon SARS-CoV-2 infection than that of mock condition (Figures 4D and 4E). No significant difference of insulin expression was detected among $ACE2^{+}NRP1^{+}$ cells, $ACE2^{+}NRP1^{-}$ cells, $ACE2^{-}NRP1^{+}$ cells, and $ACE2^{-}NRP1^{-}$ cells (Figures S4F and S4G). In addition, both the percentage of $GCG^{+}INS^{+}$ cells in INS^{+} cells and the average intensity of GCG in INS^{+} cells were significantly higher in SARS-CoV-2-infected condition than mock condition (Figures 4F–4H). Moreover, both the percentage of $trypsin1^{+}INS^{+}$ cells in INS^{+} cells and the average intensity of trypsin1 in INS^{+} cells are significantly higher in SARS-CoV-2-infected condition than mock condition (Figures 4I–4K). Together, these data suggest the beta cells show higher GCG and trypsin1 expression at both transcriptional and protein levels in SARS-CoV-2-infected condition than mock condition.

We then confirmed these changes in protein expression using the autopsy samples of COVID-19 subjects. Consistent with SARS-CoV-2-infected islets, the average intensity of insulin in INS^{+} cells was significantly lower in the autopsy samples of COVID-19 subjects than those of non-COVID-19 subjects (Figures 4L and 4M). Furthermore, both the percentage of $trypsin1^{+}INS^{+}$ cells in INS^{+} cells and the average intensity of trypsin1 in INS^{+} cells were significantly higher in the autopsy samples of COVID-19 subjects than those of non-COVID-19 subjects in all samples (Figures 4N and 4O) and age-matched groups (Figures S4H–S4J). Together, these data suggest that beta cells undergo transdifferentiation both *ex vivo* upon SARS-CoV-2 infection and in COVID-19 subjects.

Trajectory analysis identifies the eIF2 pathway as regulating beta cell transdifferentiation

To determine the pathway regulating beta cell transdifferentiation, we reconstructed a trajectory by pseudotemporal ordering of single beta cells, in which the start of the trajectory showed less SARS-CoV-2 infection while the end displayed high SARS-CoV-2 infection (Figure 5A). This trajectory analysis identified a "beta cell transdifferentiation" path. Along this path, the level of the beta cell marker *INS* decreased, while both alpha cell markers, such as *GCG*, and acinar cell markers, including *CPA1*, *PRSS1*, and *PRSS2*, increased (Figure 5B). In addition, transcript levels of SARS-CoV-2 genes, such as *SARS-CoV-2-N* and *SARS-CoV-2-ORF1ab*, increased along with beta cell transdifferentiation (Figure 5C).

Ingenuity pathway analysis also identified eIF2 signaling as the topmost pathway changed along with beta cell transdifferentiation (Figure 5D). This is consistent with eIF2 signaling being identified as the topmost pathway when comparing the genes that are differentially expressed in SARS-CoV-2- versus mock-infected beta cells (Figure 3D). A heatmap of the results showed that genes involved in the eIF2 pathway were significantly changed during beta cell transdifferentiation (Figure 5E), which indicated activation of the cellular stress response via phosphorylation of eIF2α. Western blotting confirmed the higher level of phosphorylated PKR and phosphorylated eIF2α in SARS-CoV-2-infected EndoC-BetaH1 cells than mock-treated cells (Figures S5A–S5C). The eIF2 signaling pathway is required for most forms of eukaryotic translation initiation. Protein translation was lower in SARS-CoV-2-infected human islet cells than mock-infected islet cells, including beta cells (Figures S5D and S5E). To further validate the higher level of cellular stress in SARS-CoV-2-infected condition, we applied immunostaining to examine the expression of G3BP1, which is an essential component of stress granules. Both the average intensity of stress granules (Figures 5F and 5G) and the average number of stress granules (Figures 5F and 5H) in INS^{+} cells were significantly higher in the SARS-CoV-2-infected islets compared with the mock-infected islets. Dot blots further validated the modest upregulation of cell-stress-associated genes in beta cells upon SARS-CoV-2 infection (Figure 5I).

(D and E) Representative confocal images (D) and quantification of relative INS intensity in INS^{+} cells (E) of mock- versus SARS-CoV-2-infected human islets at 48 hpi. Three images of each sample were used for quantification for each donor (n = 3 individual islet donors, MOI = 1). Scale bar, 50 μm. Green, INS; blue, DAPI.
(F–H) Representative confocal images (F) and quantification of the percentage of $GCG^{+}INS^{+}$ cells in INS^{+} cells (G) and the average GCG intensity in INS^{+} cells (H) of mock- versus SARS-CoV-2-infected human islets at 48 hpi (n = 3 individual islet donors, MOI = 1). Scale bar, 50 μm. Red, GCG; green, INS; blue: DAPI. Yellow arrows highlight $GCG^{+}INS^{+}$ cells.
(I–K) Representative confocal images (I) and quantification of the percentage of $Typsin1^{+}INS^{+}$ cells in INS^{+} cells (J) and the average Typsin1 intensity in INS^{+} cells (K) of mock- versus SARS-CoV-2-infected human islets at 48 hpi (n = 3 individual islet donors, MOI = 1). Scale bar, 50 μm. Red, trypsin1; green, INS; blue, DAPI.
(L and M) Representative confocal images (L) and quantification of the relative INS intensity in INS^{+} cells (M) of autopsy samples of non-COVID-19 and COVID-19 subjects. Three images of each sample were used for quantification for each subject. (M) n = 5 non-COVID-19 subjects; n = 5 COVID-19 subjects; scale bar, 50 μm. Red, trypsin1; green, INS; blue, DAPI.
(N and O) Quantification of the percentage of $Typsin1^{+}INS^{+}$ cells in INS^{+} cells (N) and the average Typsin1 intensity in INS^{+} cells (O) of autopsy samples of non-COVID-19 or COVID-19 subjects. Three images of each subject were used for quantification (n = 5 COVID-19 subjects; n = 5 non-COVID-19 subjects).
Data are presented as mean ± SD. p values were calculated by paired or unpaired two-tailed Student's t test. *$p < 0.05$ and ****$p < 0.0001$.

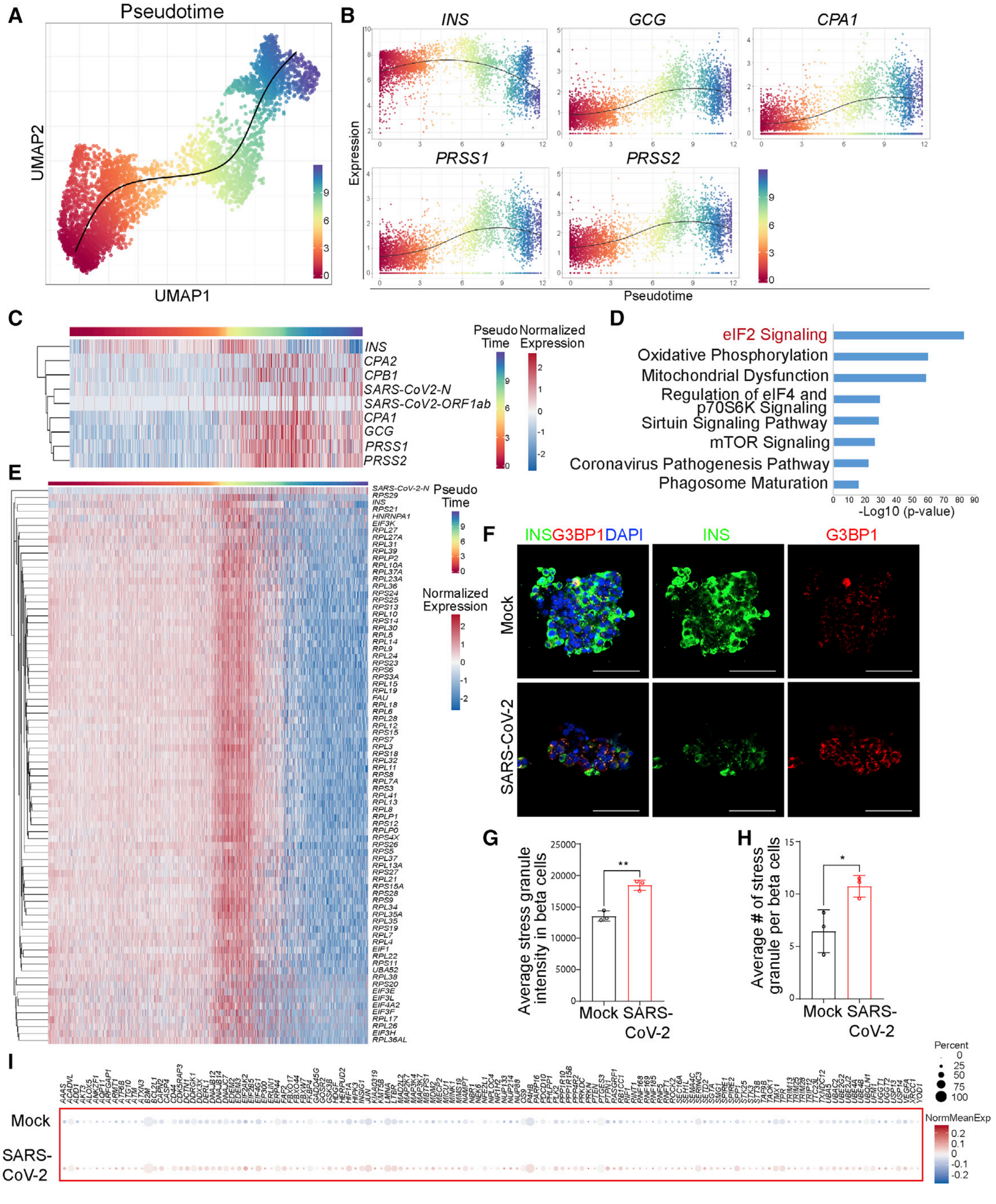

Figure 5. Trajectory analysis identifies a change of eIF2 signaling during beta cell transdifferentiation

(A) Ordering beta cells along a transdifferentiation transition in mock- and SARS-CoV-2-infected islets; each cell was assigned a "pseudotime" indicating its relative position in the transition (n = 2 individual islet donors).

(B) Changes in expression of *INS*, *GCG*, *CPA1*, *PRSS1*, and *PRSS2* during beta cell transdifferentiation (n = 2 individual islet donors).

(C) Heatmap showing expression changes in *INS*, *GCG*, *CPA1*, *CPA2*, *CPB1*, *PRSS1*, *PRSS2*, *SARS-CoV-2-N*, and *SARS-CoV-2-ORF1ab* during beta cell transdifferentiation. Cells were ordered in pseudotime (n = 2 individual islet donors).

(D) Ingenuity pathway analysis on genes changed during beta cell transdifferentiation (n = 2 individual islet donors).

(E) Heatmap showing expression changes in eIF2-pathway-associated genes during beta cell transdifferentiation. Cells were ordered in pseudotime (n = 2 individual islet donors).

(F–H) Representative confocal images (F) and quantification of the average stress granule intensity in INS^+ cells (G) and the average stress granule number in INS^+ cells (H) of mock- versus SARS-CoV-2-infected human islets at 48 hpi (n = 3 individual islet donors, MOI = 1). Scale bar, 50 μm. Data are presented as mean ± SD. p values were calculated by paired or unpaired two-tailed Student's t test. *$p < 0.05$ and **$p < 0.01$. Red, G3BP1; green, INS; blue, DAPI.

(I) Dot blot illustrating expression of cell-stress-associated genes in mock- versus SARS-CoV-2-infected human islets at 24 hpi (MOI = 1). Dot size shows the fraction of cells with non-zero expression; dot color indicates the relative expression level in the two conditions (n = 2 individual islet donors).

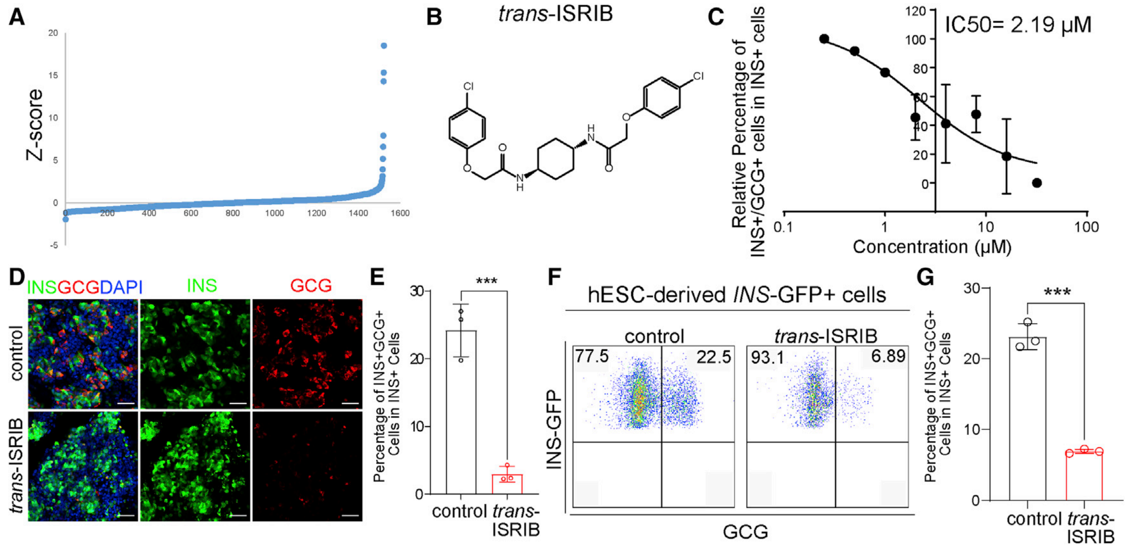

Figure 6. A high-throughput screen to identify a compound rescuing beta cell transdifferentiation

(A) Primary screen data. hESC-derived pancreatic endocrine cells were treated at 10 μM with compounds from an in-house library containing US FDA-approved drugs and signaling pathway regulators. DMSO treatment was used as a negative control. After 4 days of culture, cells were fixed and stained with antibodies against INS and GCG.

(B) Chemical structure of *trans*-ISRIB.

(C) Dose curve of *trans*-ISRIB on the relative polyhormonal rate (n = 3 biological replicates).

(D and E) Representative images (D) and quantification of the polyhormonal rate (E) of hESC-derived *INS*-GFP$^+$ cells after 4 days treatment of 4 μM *trans*-ISRIB (n = 3 biological replicates). Scale bar, 50 μm. Red, GCG; green, INS; blue, DAPI.

(F and G) Flow cytometry analysis (F) and quantification of polyhormonal rate (G) of hESC-derived *INS*-GFP$^+$ cells after 4 days of treatment of 4 μM *trans*-ISRIB (n = 3 biological replicates).

Data are presented as mean ± SD. p values were calculated by unpaired two-tailed Student's t test. ***p < 0.001.

A high-throughput chemical screen shows that *trans*-ISRIB can reverse the transdifferentiation of polyhormonal cells

Synthetic small molecules provide powerful tools to dissect the molecular pathways of biological processes. To determine the molecular pathway controlling pancreatic beta cell transdifferentiation, we performed a high-throughput chemical screen to identify small molecules that can decrease the number of transdifferentiated polyhormonal cells. It is technically challenging to perform a high-throughput screen using primary human islets and SARS-CoV-2, so we used human pluripotent stem cell (hPSC)-derived endocrine cells, which also contain a high percentage of polyhormonal cells. Briefly, we differentiated MEL-1$^{INS/GFP}$ hESCs to pancreatic GCG$^+$INS$^+$ polyhormonal cells (Amin et al., 2018; D'Amour et al., 2006). Around 20% of the derived *INS*-GFP$^+$ cells expressed glucagon. We plated the cells on 384-well plates and treated them for 4 days with a chemical library containing the US FDA-approved drugs as well as other non-approved molecules that regulate various signaling pathways. Then, we fixed and stained the cells with GCG and INS antibodies. The percentage of GCG$^+$INS$^+$ cells in *INS*-GFP$^+$ cells was defined as the polyhormonal rate.

The small molecules that decreased the *Z* score to <−1.5 were defined as primary hits (Figure 6A). After confirmation, one compound, *trans*-integrated stress response inhibitor (*trans*-ISRIB; Figure 6B), was identified to decrease the polyhormonal rate. *Trans*-ISRIB was identified from a chemical screen for small molecule inhibitors of PERK signaling (Sekine et al., 2015), which was shown to directly bind with eIF2B by cryoelectron microscopy (cryo-EM) (Zyryanova et al., 2018). Here, we found that the compound decreased the polyhormonal rate through a dose-dependent manner (Figure 6C; IC_{50} = 2.19 μM), which was independent of cytotoxicity (Figure S6A). Further, we found that the polyhormonal rate was significantly lower at 4 μM *trans*-ISRIB treatment condition than control condition, as indicated by both immunostaining (Figures 6D and 6E) and flow cytometry (Figures 6F, 6G, and S6B).

Trans-ISRIB blocks beta cell transdifferentiation upon SARS-CoV-2 infection

To determine whether *trans*-ISRIB was sufficient to block beta cell transdifferentiation upon SARS-CoV-2 infection, we treated human islets with 10 μM *trans*-ISRIB and infected with SARS-CoV-2. At 24 hpi, we applied scRNA-seq to compare the gene expression profiles of control- and *trans*-ISRIB-treated human islets. No major difference of SARS-CoV-2 gene expression was detected between *trans*-ISRIB- and control-treated human islets (Figure S6C), suggesting that *trans*-ISRIB does not affect SARS-CoV-2 infection.

Dot blots of gene expression levels suggested that the average *INS* transcriptional expression level was higher in *trans*-ISRIB-treated beta cells than that of control-treated beta cells upon SARS-CoV-2 infection (Figure 7A). *Trans*-ISRIB treatment also decreased the expression of alpha cell markers (Figure 7B) and acinar cell markers (Figure 7C) in beta cells upon SARS-CoV-2 infection. Immunostaining further validated the

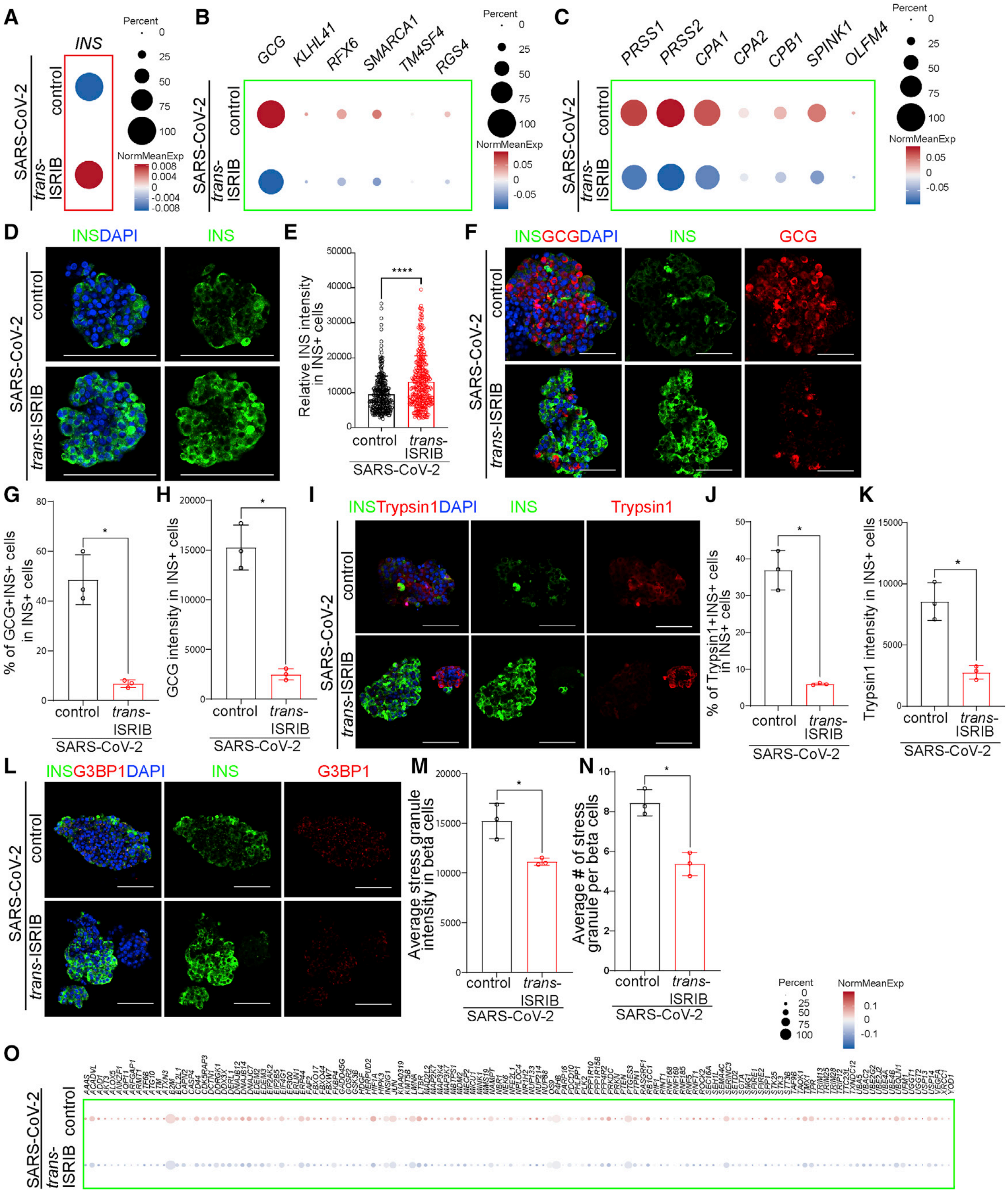

Figure 7. *Trans*-ISRIB blocks human beta cell transdifferentiation upon SARS-CoV-2 infection

(A–C) Dot blot illustrating expression level of *INS* (A); alpha cell markers, including *GCG*, *KLHL41*, *RFX6*, *SMARCA1*, *TM4SF4*, and *RGS4* (B); and acinar cell markers, including *PRSS1*, *PRSS2*, *CPA1*, *CPA2*, *CPB1*, *SPINK1*, and *OLFM4* (C), in beta cells of control or 10 μM *trans*-ISRIB-treated human islets at 24 hpi (MOI = 1). Dot size shows the fraction of cells with non-zero expression; dot color indicates the relative expression level in the two conditions (n = 1 islet donor).

(D and E) Representative confocal images (D) and quantification of relative INS intensity in INS^+ cells (E) of control or 10 μM *trans*-ISRIB-treated human islets at 48 hpi (n = 3 individual islet donors, MOI = 1). Scale bar, 50 μm. Green, INS; blue, DAPI.

(F–H) Representative confocal images (F) and quantification of the percentage of GCG^+INS^+ cells in INS^+ cells (G) and the average GCG intensity in INS^+ cells (H) of control or 10 μM *trans*-ISRIB-treated human islets at 48 hpi (n = 3 individual islet donors, MOI = 1). Scale bar, 50 μm. Red, GCG; green, INS; blue, DAPI.

(I–K) Representative confocal images (I) and quantification of the percentage of $Typsin1^+INS^+$ cells in INS^+ cells (J) and the average Typsin1 intensity in INS^+ cells (K) of control or 10 μM *trans*-ISRIB-treated human islets at 48 hpi (n = 3 individual islet donors, MOI = 1). Scale bar, 50 μm. Red, trypsin1; green, INS; blue, DAPI.

(legend continued on next page)

higher INS expression of *trans*-ISRIB treated beta cells in SARS-CoV-2-infected condition than mock condition (Figures 7D and 7E). In addition, both the percentage of GCG^+INS^+ cells in INS^+ cells and the average intensity of GCG in INS^+ cells are significantly lower in *trans*-ISRIB-treated beta cells in comparison with control-treated condition upon SARS-CoV-2 infection than mock condition (Figures 7F–7H and S7A). Moreover, both the percentage of trypsin1$^+$INS$^+$ cells in INS^+ cells and the average intensity of trypsin1 in INS^+ cells were significantly lower in *trans*-ISRIB-treated beta cells than control-treated beta cells upon SARS-CoV-2 infection (Figures 7I–7K and S7B).

The protein translation of SARS-CoV-2-infected human islets was higher in *trans*-ISRIB-treated condition than control condition (Figures S7C and S7D). Immunostaining further validated the lower average stress granule intensity (Figures 7L and 7M) and the lower number of stress granules in *trans*-ISRIB-treated INS^+ cells than control-treated INS^+ cells upon SARS-CoV-2 infection (Figures 7L, 7N, and S7E). Furthermore, the cell-stress-associated genes were downregulated in *trans*-ISRIB-treated beta cells compared with control-treated cells upon SARS-CoV-2 infection (Figure 7O). We further examined the impact of *trans*-ISRIB on arsenite-treated human islets, as this compound has been widely used to induce stress granule formation (Wheeler et al., 2016). *Trans*-ISIRB also rescued arsenite-induced increase of stress granule in human islets (Figures S7F and S7G), suggesting that the effect of *trans*-ISIRB on SARS-CoV-2-induced cell stress is not specific to that form of stimulation of the pathway. Together, these data indicate that SARS-CoV-2-induced beta cell transdifferentiation is mediated through the eIF2 pathway and that this program can be effectively abrogated by *trans*-ISRIB.

Recently, Kusmartseva et al. (2020) and Coate et al. (2020) discussed the permissiveness of human beta cells to SARS-CoV-2 infection based on ACE2 and TMPRSS2 expression. However, overall *ACE2* expression based on scRNA-seq analyses is low, and TMPRSS2 is not essential for SARS-CoV-2 entry, as we noted in the introduction. Here, we directly validated the detection of SARS-CoV-2 viral antigen in pancreatic beta cells using autopsy samples of COVID-19 subjects.

In addition, we applied scRNA-seq to systematically explore transcriptional changes at the single-cell level and found that beta cells transdifferentiate upon SARS-CoV-2 infection. Alteration of beta cell identity has been reported in both type 1 and type 2 diabetes. Pancreatic beta cell dedifferentiation was first reported using mice with somatic deletion of *Foxo1* in beta cells (Talchai et al., 2012) and further confirmed in human type 2 diabetes islets (Cinti et al., 2016; Spijker et al., 2015). Beta cell dedifferentiation is manifested by reduced expression of beta cell markers, including key transcription factors, insulin, glucose metabolism genes, protein processing, and secretory pathway genes. The presence of progenitor cell markers has also been observed in the dedifferentiated islets beta cells of diabetic animals (Kim-Müller et al., 2014; Wang et al., 2014), along with the upregulation of alpha cell markers (Brereton et al., 2014; Cinti et al., 2016; Spijker et al., 2015). Consistent with these earlier works, an emerging study using beta cell-specific IRE1α knockout NOD mice led to beta cell dedifferentiation as evidenced by the downregulation of Ins1 and Ins2, and the upregulation of GCG, which prevented T1D progression (Lee et al., 2020). Here, in beta cells we found a lower expression of beta cell marker *INS* and higher levels of alpha cell markers, including *GCG*, *KLHL41*, *RFX6*, *SMARCA1*, *TM4SF4*, and *RGS4*, in SARS-CoV-2-infected condition than in mock condition. Interestingly, we also detected the upregulation of acinar cell markers, including *PRSS1*, *PRSS2*, *CPA1*, *CPA2*, *CPB1*, *SPINK1*, and *OLFM4*. We further validated the upregulation of trypsin1 in beta cells of autopsy samples of COVID-19 subjects. The expression of GCG was not significantly changed in beta cells of COVID-19 subjects, suggesting that the upregulation of GCG might be transient in comparison to the prolonged elevation of trypsin1. Although most beta cell dedifferentiation studies have focused on endocrine cell markers, co-expression of acinar cell marker, CPA, and INS has been reported in type 2 diabetes islets (Masini et al., 2017), suggesting the involvement of acinar cell marker upregulation in beta cell dedifferentiation or transdifferentiation. We also monitored the expression of *ALDH1A3*, a marker of dedifferentiated human beta cells (Cinti et al., 2016). Although *ALDH1A3* is only expressed in a very small proportion of human beta cells, its expression is higher in SARS-CoV-2-infected beta cells than in mock-infected beta cells (Figure S7H), which is reversed by *trans*-ISRIB treatment (Figure S7I). Another study showed polyinosinic-polycytidylic acid, a synthetic double-stranded RNA that mimics a common byproduct of viral replication, diminishes beta cell-specific gene expression and increases the expression of SOX9, a progenitor marker in EndoC-betaH1 cells (Oshima et al., 2018). Our study provides the previously unreported evidence of SARS-CoV-2-induced beta cell transdifferentiation. SARS-CoV-2 infection also causes upregulation of chemokines and cytokines. Considering non-endocrine cells, such as endothelial cells and ductal cells, can also be infected by SARS-CoV-2, we cannot fully exclude the possibility that the non-endocrine cells infected by SARS-CoV-2 secrete chemokines and cytokines and contribute to beta cell transdifferentiation.

To dissect the molecular mechanism controlling beta cell transdifferentiation in SARS-CoV-2-infected human islets, we performed a high-throughput chemical screen and identified *trans*-ISRIB, which reduces the co-expression of hormonal markers (polyhormonal rate) of hESC-derived endocrine cells. In conjunction, trajectory analysis identified the change of eIF2 signaling during beta cell transdifferentiation. *Trans*-ISRIB was shown to reverse the effects of eIF2α phosphorylation (Camunas-Soler et al., 2020; Sidrauski et al., 2015). Together, this suggests that the presence of aberrant viral RNA and the subsequent change

(L–N) Representative confocal images (L) and quantification of the average stress granule intensity in INS^+ cells (M) and the average stress granule number in INS^+ cells (N) of control or 10 μM *trans*-ISRIB-treated human islets at 48 hpi (n = 3 individual islet donors, MOI = 1). Scale bar, 50 μm. Red, G3BP1; green, INS; blue, DAPI.

(O) Dot blot illustrating expression levels of cell-stress-associated genes of control or 10 μM *trans*-ISRIB-treated human islets at 24 hpi (MOI = 1). Dot size shows the fraction of cells with non-zero expression; dot color indicates the relative expression level in the two conditions (n = 1 islet donor).

Data are presented as mean ± SD. p values were calculated by unpaired two-tailed Student's t test. $^*p < 0.05$.

of the eIF2 pathway plays a causal role in beta cell transdifferentiation upon SARS-CoV-2 infection. eIF2, a eukaryotic translation factor required for most forms of translation initiation, is regulated by a mechanism involving both guanine nucleotide exchange and phosphorylation (Adomavicius et al., 2019). Phosphorylation takes place at the alpha subunit of eIF2 by a number of stress-activated serine kinases, such as GCN2 activated by amino acid deprivation (Wek et al., 1995), PERK caused by ER stress (Teske et al., 2011), PKR stimulated by dsRNA (Vyas et al., 2003), or HRI induced by heavy metal (Matts et al., 1991). Here, we found that both phosphorylated PKR and phosphorylated eIF2α were higher in SARS-CoV-2-infected beta cells than in mock-infected beta cells. Beta cells are vulnerable to ER stress due to their elevated rate of insulin biosynthesis in response to glucose stimulation. Inactivation of the adaptive UPR has been associated with beta cell transdifferentiation (Chan et al., 2013). Consistent with these data, we also detected higher cellular stress in SARS-CoV-2-infected beta cells than in mock-infected beta cells, which was relieved by *trans*-ISRIB treatment.

In summary, we performed immunostaining using the autopsy samples of COVID-19 subjects, as well as scRNA-seq analysis and immunostaining on SARS-CoV-2-infected human islets to provide direct evidence that pancreatic endocrine cells, in particularly beta cells, can be infected by SARS-CoV-2. Moreover, human beta cells undergo eIF2-mediated transdifferentiation upon SARS-CoV-2 infection. Finally, we performed a high-throughput screen and identified *trans*-ISRIB as a small molecule that blocks SARS-CoV-2-induced beta cell transdifferentiation, suggesting eIF2 as a potential target to develop therapy to prevent or reverse beta cell transdifferentiation in diabetes.

Limitations of study

These data implicate the detection of viral antigen in the pancreatic autopsy sample of COVID-19 subjects. However, the limitations of working with autopsy samples prevented us from being able to clearly distinguish whether the detection of viral antigen is the result of direct infection of these cells *in vivo* or the result of exposure to viral debris sufficient to induce an antiviral response as reported in small animal models (Boudewijns et al., 2020; Hoagland et al., 2021). Moreover, the heterogeneity of human islets from different individuals might affect scRNA-seq analysis. Additional islets from subjects of different gender and ethnic backgrounds will further strengthen the conclusion. Finally, the consequence of transdifferentiation is not clear. While transdifferentiation might be averted by restoring eIF2 pathway, additional work still needs to be done to determine whether long-term inhibition of such responses could be a treatment modality caused by viral infection.

STAR★METHODS

Detailed methods are provided in the online version of this paper and include the following:

- KEY RESOURCES TABLE
- RESOURCE AVAILABILITY
  - Lead contact
  - Materials availability
  - Data and code availability
- EXPERIMENTAL MODEL AND SUBJECT DETAILS
  - Cell lines
  - Human pancreatic islets
  - Human subjects
- METHOD DETAILS
  - SARS-CoV-2 propagation and infection
  - scRNA-seq of viral infections
  - Sequencing and gene expression UMI counts matrix generation
  - Single-cell RNA-seq data analysis
  - Immunofluorescence staining and confocal microscopy
  - Insulin secretion assays
  - Plaque assays
  - OPP staining
  - hESC maintenance and pancreatic endocrine cell differentiation
  - ELISA Analysis
  - High throughput chemical screening
  - Flow cytometry and intracellular FACS analysis
  - Western blot
  - qRT-PCR
- QUANTIFICATION AND STATISTICAL ANALYSIS

SUPPLEMENTAL INFORMATION

Supplemental information can be found online at https://doi.org/10.1016/j.cmet.2021.05.015.

ACKNOWLEDGMENTS

This work was supported by the National Institute of Diabetes, Digestive and Kidney Diseases (NIDDK, DP3DK111907, R01DK116075, R01DK119667, R01DK119667-02S1, R01 DK124463, and U01 DK127777, S.C.); American Diabetes Association (7–20-COVID-211, S.C.); Department of Surgery, Weill Cornell Medicine (T.E. and S.C.); Bill and Melinda Gates Foundation (S.C., T.E., R.E.S., and B.R.T.); National Cancer Institute (NCI) R01CA234614, National Institute of Allergy and Infectious Diseases (NIAID) 2R01AI107301, and NIDDK R01DK121072 and 1RO3DK117252; Department of Medicine, Weill Cornell Medicine (R.E.S.); the Defense Advanced Research Projects Agency (W911NF-16-C-0050, B.R.T.); the Marc Haas Foundation (B.R.T.); National Institutes of Health Intramural Research Program of the National Human Genome Research Institute (ZIA HG000024, F.S.C.); and F32 postdoctoral fellowship (Eunice Kennedy Shriver National Institute of Child Health and Human Development, NICHD 1F32HD096810-01A1, L.A.L.). S.C. and R.E.S. are supported as Irma Hirschl Trust Research Award Scholars. Human islets received from the University of Pennsylvania human islet center were funded by the NIDDK-supported Human Pancreas Analysis Program (HPAP) (https://hpap.pmacs.upenn.edu/citation) grants UC4 DK112217 to A.N. We also thank Drs. Tingfen Yan and Chad Krilow of the Collins group for data processing and bioinformatics support, as well as Dr. Kohei Oishi of the tenOever lab for providing data for SARS-CoV-2 growth curves in Vero E6 cells.

AUTHOR CONTRIBUTIONS

S.C., R.E.S., T.E., F.S.C., and B.R.T. conceived the project. S.C. and X.T. designed the experiments. X.T. performed human islets culture, confocal imaging, western blot, and flow cytometry analysis. S.U. performed SARS-CoV-2-related experiments and insulin secretion study. X.T. and D.X. performed immunostaining and quantification. B.L. performed high-throughput chemical screen. L.A.L. performed 3D reconstruction of confocal images. J.J.V. and A.C.N.C. provided EndoC-betaH1 cells. C.L., Z.M., and A.N. performed the human islet isolation. J.A.A. and J.K.L. performed ELISA analysis. T.Z., J.X., L.L.B., N.N., and M.R.E. performed the scRNA-seq and bioinformatics

CellPress
OPEN ACCESS

analysis. R.E.S., Y.B., V.C., and A.C.B. collected autopsy samples and performed qRT-PCR. S.C., X.T., S.U., T.Z., and D.X. analyzed the data and wrote the manuscript.

DECLARATION OF INTERESTS

R.E.S. is on the scientific advisory board of Miromatrix Inc. and is a paid consultant and speaker for Alnylam Inc. The other authors declare no competing interests.

Received: December 30, 2020
Revised: March 30, 2021
Accepted: May 7, 2021
Published: May 19, 2021

REFERENCES

Adomavicius, T., Guaita, M., Zhou, Y., Jennings, M.D., Latif, Z., Roseman, A.M., and Pavitt, G.D. (2019). The structural basis of translational control by eIF2 phosphorylation. Nat. Commun. *10*, 2136.

Amin, S., Cook, B., Zhou, T., Ghazizadeh, Z., Lis, R., Zhang, T., Khalaj, M., Crespo, M., Perera, M., Xiang, J.Z., et al. (2018). Discovery of a drug candidate for GLIS3-associated diabetes. Nat. Commun. *9*, 2681.

Ashary, N., Bhide, A., Chakraborty, P., Colaco, S., Mishra, A., Chhabria, K., Jolly, M.K., and Modi, D. (2020). Single-cell RNA-seq identifies cell subsets in human placenta that highly expresses factors driving pathogenesis of SARS-CoV-2. Front. Cell Dev. Biol. *8*, 783.

Baron, M., Veres, A., Wolock, S.L., Faust, A.L., Gaujoux, R., Vetere, A., Ryu, J.H., Wagner, B.K., Shen-Orr, S.S., Klein, A.M., et al. (2016). A single-cell transcriptomic map of the human and mouse pancreas reveals inter- and intra-cell population structure. Cell Syst *3*, 346–360.e4.

Blanco-Melo, D., Nilsson-Payant, B.E., Liu, W.C., Uhl, S., Hoagland, D., Møller, R., Jordan, T.X., Oishi, K., Panis, M., Sachs, D., et al. (2020). Imbalanced host response to SARS-CoV-2 drives development of COVID-19. Cell *181*, 1036–1045.e9.

Boudewijns, R., Thibaut, H.J., Kaptein, S.J.F., Li, R., Vergote, V., Seldeslachts, L., Van Weyenbergh, J., De Keyzer, C., Bervoets, L., Sharma, S., et al. (2020). STAT2 signaling restricts viral dissemination but drives severe pneumonia in SARS-CoV-2 infected hamsters. Nat. Commun. *11*, 5838.

Brereton, M.F., Iberl, M., Shimomura, K., Zhang, Q., Adriaenssens, A.E., Proks, P., Spiliotis, I.I., Dace, W., Mattis, K.K., Ramracheya, R., et al. (2014). Reversible changes in pancreatic islet structure and function produced by elevated blood glucose. Nat. Commun. *5*, 4639.

Camunas-Soler, J., Dai, X.Q., Hang, Y., Bautista, A., Lyon, J., Suzuki, K., Kim, S.K., Quake, S.R., and MacDonald, P.E. (2020). Patch-seq links single-cell transcriptomes to human islet dysfunction in diabetes. Cell Metab *31*, 1017–1031.e4.

Cantuti-Castelvetri, L., Ojha, R., Pedro, L.D., Djannatian, M., Franz, J., Kuivanen, S., van der Meer, F., Kallio, K., Kaya, T., Anastasina, M., et al. (2020). Neuropilin-1 facilitates SARS-CoV-2 cell entry and infectivity. Science *370*, 856–860.

Chan, J.Y., Luzuriaga, J., Bensellam, M., Biden, T.J., and Laybutt, D.R. (2013). Failure of the adaptive unfolded protein response in islets of obese mice is linked with abnormalities in beta-cell gene expression and progression to diabetes. Diabetes *62*, 1557–1568.

Chee, Y.J., Ng, S.J.H., and Yeoh, E. (2020). Diabetic ketoacidosis precipitated by Covid-19 in a patient with newly diagnosed diabetes mellitus. Diabetes Res. Clin. Pract. *164*, 108166.

Cinti, F., Bouchi, R., Kim-Muller, J.Y., Ohmura, Y., Sandoval, P.R., Masini, M., Marselli, L., Suleiman, M., Ratner, L.E., Marchetti, P., and Accili, D. (2016). Evidence of beta-cell dedifferentiation in human type 2 diabetes. J. Clin. Endocrinol. Metab. *101*, 1044–1054.

Coate, K.C., Cha, J., Shrestha, S., Wang, W., Gonçalves, L.M., Almaça, J., Kapp, M.E., Fasolino, M., Morgan, A., Dai, C., et al. (2020). SARS-CoV-2 cell entry factors ACE2 and TMPRSS2 are expressed in the microvasculature and ducts of human pancreas but are not enriched in β cells. Cell Metab *32*, 1028–1040.e4.

Cummings, M.J., Baldwin, M.R., Abrams, D., Jacobson, S.D., Meyer, B.J., Balough, E.M., Aaron, J.G., Claassen, J., Rabbani, L.E., Hastie, J., et al. (2020). Epidemiology, clinical course, and outcomes of critically ill adults with COVID-19 in New York City: a prospective cohort study. Lancet *395*, 1763–1770.

Daly, J.L., Simonetti, B., Klein, K., Chen, K.E., Williamson, M.K., Antón-Plágaro, C., Shoemark, D.K., Simón-Gracia, L., Bauer, M., Hollandi, R., et al. (2020). Neuropilin-1 is a host factor for SARS-CoV-2 infection. Science *370*, 861–865.

D'Amour, K.A., Bang, A.G., Eliazer, S., Kelly, O.G., Agulnick, A.D., Smart, N.G., Moorman, M.A., Kroon, E., Carpenter, M.K., and Baetge, E.E. (2006). Production of pancreatic hormone-expressing endocrine cells from human embryonic stem cells. Nat. Biotechnol. *24*, 1392–1401.

Daniloski, Z., Jordan, T.X., Wessels, H.H., Hoagland, D.A., Kasela, S., Legut, M., Maniatis, S., Mimitou, E.P., Lu, L., Geller, E., et al. (2021). Identification of required host factors for SARS-CoV-2 infection in human cells. Cell *184*, 92–105.e16.

Enge, M., Arda, H.E., Mignardi, M., Beausang, J., Bottino, R., Kim, S.K., and Quake, S.R. (2017). Single-cell analysis of human pancreas reveals transcriptional signatures of aging and somatic mutation patterns. Cell *171*, 321–330.e14.

Fadini, G.P., Morieri, M.L., Boscari, F., Fioretto, P., Maran, A., Busetto, L., Bonora, B.M., Selmin, E., Arcidiacono, G., Pinelli, S., et al. (2020). Newly-diagnosed diabetes and admission hyperglycemia predict COVID-19 severity by aggravating respiratory deterioration. Diabetes Res. Clin. Pract. *168*, 108374.

Han, Y., Duan, X., Yang, L., Nilsson-Payant, B.E., Wang, P., Duan, F., Tang, X., Yaron, T.M., Zhang, T., Uhl, S., et al. (2021). Identification of SARS-CoV-2 inhibitors using lung and colonic organoids. Nature *589*, 270–275.

Heaney, A.I., Griffin, G.D., and Simon, E.L. (2020). Newly diagnosed diabetes and diabetic ketoacidosis precipitated by COVID-19 infection. Am. J. Emerg. Med. *38*, 2491.e3–2491.e4.

Hoagland, D.A., Møller, R., Uhl, S.A., Oishi, K., Frere, J., Golynker, I., Horiuchi, S., Panis, M., Blanco-Melo, D., Sachs, D., et al. (2021). Leveraging the antiviral type I interferon system as a first line of defense against SARS-CoV-2 pathogenicity. Immunity *54*, 557–570.e5.

Hoffmann, M., Kleine-Weber, H., Schroeder, S., Krüger, N., Herrler, T., Erichsen, S., Schiergens, T.S., Herrler, G., Wu, N.-H., Nitsche, A., et al. (2020). SARS-CoV-2 cell entry depends on ACE2 and TMPRSS2 and is blocked by a clinically proven protease inhibitor. Cell *181*, 271–280.e8.

Hollstein, T., Schulte, D.M., Schulz, J., Glück, A., Ziegler, A.G., Bonifacio, E., Wendorff, M., Franke, A., Schreiber, S., Bornstein, S.R., and Laudes, M. (2020). Autoantibody-negative insulin-dependent diabetes mellitus after SARS-CoV-2 infection: a case report. Nat. Metab. *2*, 1021–1024.

Hou, Y.J., Okuda, K., Edwards, C.E., Martinez, D.R., Asakura, T., Dinnon, K.H., 3rd, Kato, T., Lee, R.E., Yount, B.L., Mascenik, T.M., et al. (2020). SARS-CoV-2 reverse genetics reveals a variable infection gradient in the respiratory tract. Cell *182*, 429–446.e14.

Kim-Müller, J.Y., Zhao, S., Srivastava, S., Mugabo, Y., Noh, H.L., Kim, Y.R., Madiraju, S.R., Ferrante, A.W., Skolnik, E.Y., Prentki, M., and Accili, D. (2014). Metabolic inflexibility impairs insulin secretion and results in MODY-like diabetes in triple FoxO-deficient mice. Cell Metab *20*, 593–602.

Kusmartseva, I., Wu, W., Syed, F., Van Der Heide, V., Jorgensen, M., Joseph, P., Tang, X., Candelario-Jalil, E., Yang, C., Nick, H., et al. (2020). Expression of SARS-CoV-2 entry factors in the pancreas of normal organ donors and individuals with COVID-19. Cell Metab *32*, 1041–1051.e6.

Lee, H., Lee, Y.S., Harenda, Q., Pietrzak, S., Oktay, H.Z., Schreiber, S., Liao, Y., Sonthalia, S., Ciecko, A.E., Chen, Y.-G., et al. (2020). Beta cell dedifferentiation induced by IRE1alpha deletion prevents type 1 diabetes. Cell Metab *31*, 822–836.e5.

Li, J., Klughammer, J., Farlik, M., Penz, T., Spittler, A., Barbieux, C., Berishvili, E., Bock, C., and Kubicek, S. (2016). Single-cell transcriptomes reveal characteristic features of human pancreatic islet cell types. EMBO Rep *17*, 178–187.

Li, H., Tian, S., Chen, T., Cui, Z., Shi, N., Zhong, X., Qiu, K., Zhang, J., Zeng, T., Chen, L., and Zheng, J. (2020). Newly diagnosed diabetes is associated with a higher risk of mortality than known diabetes in hospitalized patients with COVID-19. Diabetes Obes. Metab. *22*, 1897–1906.

Liang, X., Xu, J., Xiao, W., Shi, L., and Yang, H. (2021). The association of diabetes with COVID-19 disease severity: evidence from adjusted effect estimates. Hormones (Athens) *20*, 409–414.

Liu, F., Long, X., Zhang, B., Zhang, W., Chen, X., and Zhang, Z. (2020). ACE2 expression in pancreas may cause pancreatic damage After SARS-CoV-2 infection. Clin. Gastroenterol. Hepatol. *18*, 2128–2130.e2.

Masini, M., Marselli, L., Himpe, E., Martino, L., Bugliani, M., Suleiman, M., Boggi, U., Filipponi, F., Occhipinti, M., Bouwens, L., et al. (2017). Co-localization of acinar markers and insulin in pancreatic cells of subjects with type 2 diabetes. PLoS One *12*, e0179398.

Matts, R.L., Schatz, J.R., Hurst, R., and Kagen, R. (1991). Toxic heavy metal ions activate the heme-regulated eukaryotic initiation factor-2 alpha kinase by inhibiting the capacity of hemin-supplemented reticulocyte lysates to reduce disulfide bonds. J. Biol. Chem. *266*, 12695–12702.

Müller, J.A., Groß, R., Conzelmann, C., Krüger, J., Merle, U., Steinhart, J., Weil, T., Koepke, L., Bozzo, C.P., Read, C., et al. (2021). SARS-CoV-2 infects and replicates in cells of the human endocrine and exocrine pancreas. Nat. Metab. *3*, 149–165.

Muraro, M.J., Dharmadhikari, G., Grün, D., Groen, N., Dielen, T., Jansen, E., van Gurp, L., Engelse, M.A., Carlotti, F., de Koning, E.J., and van Oudenaarden, A. (2016). A single-cell transcriptome atlas of the human pancreas. Cell Syst *3*, 385–394.e3.

Ortiz Bezara, M.E., Thurman, A., Pezzulo, A.A., Leidinger, M.R., Klesney-Tait, J.A., Karp, P.H., Tan, P., Wohlford-Lenane, C., McCray, P.B., and Meyerholz, D.K. (2020). Heterogeneous expression of the SARS-Coronavirus-2 receptor ACE2 in the human respiratory tract. bioRxiv. https://doi.org/10.1101/2020.04.22.056127.

Oshima, M., Knoch, K.P., Diedisheim, M., Petzold, A., Cattan, P., Bugliani, M., Marchetti, P., Choudhary, P., Huang, G.C., Bornstein, S.R., et al. (2018). Virus-like infection induces human beta cell dedifferentiation. JCI Insight *3*, e97732.

Ou, X., Liu, Y., Lei, X., Li, P., Mi, D., Ren, L., Guo, L., Guo, R., Chen, T., Hu, J., et al. (2020). Characterization of spike glycoprotein of SARS-CoV-2 on virus entry and its immune cross-reactivity with SARS-CoV. Nat. Commun. *11*, 1620.

Qi, J., Zhou, Y., Hua, J., Zhang, L., Bian, J., Liu, B., Zhao, Z., and Jin, S. (2020). The scRNA-seq expression profiling of the receptor ACE2 and the cellular protease TMPRSS2 reveals human organs susceptible to COVID-19 infection. bioRxiv. https://doi.org/10.1101/2020.04.16.045690.

Qiu, W.L., Zhang, Y.W., Feng, Y., Li, L.C., Yang, L., and Xu, C.R. (2017). Deciphering pancreatic islet beta cell and alpha cell maturation pathways and characteristic features at the single-cell level. Cell Metab *25*, 1194–1205.e4.

Ricordi, C., Goldstein, J.S., Balamurugan, A.N., Szot, G.L., Kin, T., Liu, C., Czarniecki, C.W., Barbaro, B., Bridges, N.D., Cano, J., et al. (2016). National institutes of health-sponsored clinical islet transplantation consortium phase 3 trial: manufacture of a complex cellular product at eight processing facilities. Diabetes *65*, 3418–3428.

Schneider, W.M., Luna, J.M., Hoffmann, H.H., Sánchez-Rivera, F.J., Leal, A.A., Ashbrook, A.W., Le Pen, J., Ricardo-Lax, I., Michailidis, E., Peace, A., et al. (2021). Genome-scale identification of SARS-CoV-2 and pan-coronavirus host factor networks. Cell *184*, 120–132.e14.

Segerstolpe, Å., Palasantza, A., Eliasson, P., Andersson, E.M., Andréasson, A.C., Sun, X., Picelli, S., Sabirsh, A., Clausen, M., Bjursell, M.K., et al. (2016). Single-cell transcriptome profiling of human pancreatic islets in health and type 2 diabetes. Cell Metab *24*, 593–607.

Sekine, Y., Zyryanova, A., Crespillo-Casado, A., Fischer, P.M., Harding, H.P., and Ron, D. (2015). Stress responses. Mutations in a translation initiation factor identify the target of a memory-enhancing compound. Science *348*, 1027–1030.

Shang, J., Wan, Y., Luo, C., Ye, G., Geng, Q., Auerbach, A., and Li, F. (2020). Cell entry mechanisms of SARS-CoV-2. Proc. Natl. Acad. Sci. USA *117*, 11727–11734.

Sidrauski, C., McGeachy, A.M., Ingolia, N.T., and Walter, P. (2015). The small molecule ISRIB reverses the effects of eIF2alpha phosphorylation on translation and stress granule assembly. eLife *4*, e05033.

Soliman, A.T., Al-Amri, M., Alleethy, K., Alaaraj, N., Hamed, N., and De Sanctis, V. (2020). Newly-onset type 1 diabetes mellitus precipitated by COVID-19 in an 8-month-old infant. Acta Biomed *91*. https://doi.org/10.23750/abm.v91i3.10074.

Spijker, H.S., Song, H., Ellenbroek, J.H., Roefs, M.M., Engelse, M.A., Bos, E., Koster, A.J., Rabelink, T.J., Hansen, B.C., Clark, A., et al. (2015). Loss of beta-cell identity occurs in type 2 diabetes and is associated with islet amyloid deposits. Diabetes *64*, 2928–2938.

Sungnak, W., Huang, N., Bécavin, C., Berg, M., Queen, R., Litvinukova, M., Talavera-López, C., Maatz, H., Reichart, D., Sampaziotis, F., et al. (2020). SARS-CoV-2 entry factors are highly expressed in nasal epithelial cells together with innate immune genes. Nat. Med. *26*, 681–687.

Talchai, C., Xuan, S., Lin, H.V., Sussel, L., and Accili, D. (2012). Pancreatic beta cell dedifferentiation as a mechanism of diabetic beta cell failure. Cell *150*, 1223–1234.

Teske, B.F., Wek, S.A., Bunpo, P., Cundiff, J.K., McClintick, J.N., Anthony, T.G., and Wek, R.C. (2011). The eIF2 kinase PERK and the integrated stress response facilitate activation of ATF6 during endoplasmic reticulum stress. Mol. Biol. Cell *22*, 4390–4405.

Unsworth, R., Wallace, S., Oliver, N.S., Yeung, S., Kshirsagar, A., Naidu, H., Kwong, R.M.W., Kumar, P., and Logan, K.M. (2020). New-onset type 1 diabetes in children during COVID-19: multicenter regional findings in the U.K. Diabetes Care *43*, e170–e171.

Vyas, J., Elia, A., and Clemens, M.J. (2003). Inhibition of the protein kinase PKR by the internal ribosome entry site of hepatitis C virus genomic RNA. RNA *9*, 858–870.

Wang, Z., York, N.W., Nichols, C.G., and Remedi, M.S. (2014). Pancreatic beta cell dedifferentiation in diabetes and redifferentiation following insulin therapy. Cell Metab *19*, 872–882.

Wang, Y.J., Schug, J., Won, K.J., Liu, C., Naji, A., Avrahami, D., Golson, M.L., and Kaestner, K.H. (2016). Single-cell transcriptomics of the human endocrine pancreas. Diabetes *65*, 3028–3038.

Wang, S., Ma, P., Zhang, S., Song, S., Wang, Z., Ma, Y., Xu, J., Wu, F., Duan, L., Yin, Z., et al. (2020). Fasting blood glucose at admission is an independent predictor for 28-day mortality in patients with COVID-19 without previous diagnosis of diabetes: a multi-centre retrospective study. Diabetologia *63*, 2102–2111.

Wang, R., Simoneau, C.R., Kulsuptrakul, J., Bouhaddou, M., Travisano, K.A., Hayashi, J.M., Carlson-Stevermer, J., Zengel, J.R., Richards, C.M., Fozouni, P., et al. (2021). Genetic screens identify host factors for SARS-CoV-2 and common cold coronaviruses. Cell *184*, 106–119.e14.

Wei, J., Alfajaro, M.M., DeWeirdt, P.C., Hanna, R.E., Lu-Culligan, W.J., Cai, W.L., Strine, M.S., Zhang, S.M., Graziano, V.R., Schmitz, C.O., et al. (2021). Genome-wide CRISPR screens reveal host factors critical for SARS-CoV-2 infection. Cell *184*, 76–91.e13.

Wek, S.A., Zhu, S., and Wek, R.C. (1995). The histidyl-tRNA synthetase-related sequence in the eIF-2 alpha protein kinase GCN2 interacts with tRNA and is required for activation in response to starvation for different amino acids. Mol. Cell. Biol. *15*, 4497–4506.

Wheeler, J.R., Matheny, T., Jain, S., Abrisch, R., and Parker, R. (2016). Distinct stages in stress granule assembly and disassembly. eLife *5*, e18413.

Xin, Y., Kim, J., Okamoto, H., Ni, M., Wei, Y., Adler, C., Murphy, A.J., Yancopoulos, G.D., Lin, C., and Gromada, J. (2016). RNA sequencing of single human islet cells reveals type 2 diabetes genes. Cell Metab *24*, 608–615.

Xin, Y., Dominguez Gutierrez, G., Okamoto, H., Kim, J., Lee, A.H., Adler, C., Ni, M., Yancopoulos, G.D., Murphy, A.J., and Gromada, J. (2018). Pseudotime ordering of single human beta-cells reveals states of insulin production and unfolded protein response. Diabetes *67*, 1783–1794.

Yang, J.-K., Jin, J.-M., Liu, S., Bai, P., He, W., Wu, F., Liu, X.-F., Chai, Z.-L., and Han, D.-M. (2020a). New onset COVID-19-related diabetes: an indicator of mortality. medRxiv. https://doi.org/10.1101/2020.04.08.20058040.

Yang, L., Han, Y., Nilsson-Payant, B.E., Gupta, V., Wang, P., Duan, X., Tang, X., Zhu, J., Zhao, Z., Jaffré, F., et al. (2020b). A human pluripotent stem cell-based platform to study SARS-CoV-2 tropism and model virus infection in human cells and organoids. Cell Stem Cell *27*, 125–136.e7.

Zeng, C., Mulas, F., Sui, Y., Guan, T., Miller, N., Tan, Y., Liu, F., Jin, W., Carrano, A.C., Huising, M.O., et al. (2017). Pseudotemporal ordering of single cells reveals metabolic control of postnatal beta cell proliferation. Cell Metab *25*, 1160–1175.e11.

Zhou, F., Yu, T., Du, R., Fan, G., Liu, Y., Liu, Z., Xiang, J., Wang, Y., Song, B., Gu, X., et al. (2020). Clinical course and risk factors for mortality of adult inpatients with COVID-19 in Wuhan, China: a retrospective cohort study. Lancet *395*, 1054–1062.

Zhu, L., She, Z.G., Cheng, X., Qin, J.J., Zhang, X.J., Cai, J., Lei, F., Wang, H., Xie, J., Wang, W., et al. (2020). Association of blood glucose control and outcomes in patients with COVID-19 and pre-existing type 2 diabetes. Cell Metab *31*, 1068–1077.e3.

Ziegler, C.G.K., Allon, S.J., Nyquist, S.K., Mbano, I.M., Miao, V.N., Tzouanas, C.N., Cao, Y., Yousif, A.S., Bals, J., Hauser, B.M., et al. (2020). SARS-CoV-2 receptor ACE2 is an interferon-stimulated gene in human airway epithelial cells and is detected in specific cell subsets across tissues. Cell *181*, 1016–1035.e19.

Zou, X., Chen, K., Zou, J., Han, P., Hao, J., and Han, Z. (2020). Single-cell RNA-seq data analysis on the receptor ACE2 expression reveals the potential risk of different human organs vulnerable to 2019-nCoV infection. Front. Med. *14*, 185–192.

Zyryanova, A.F., Weis, F., Faille, A., Alard, A.A., Crespillo-Casado, A., Sekine, Y., Harding, H.P., Allen, F., Parts, L., Fromont, C., et al. (2018). Binding of ISRIB reveals a regulatory site in the nucleotide exchange factor eIF2B. Science *359*, 1533–1536.

STAR★METHODS

KEY RESOURCES TABLE

REAGENT or RESOURCE	SOURCE	IDENTIFIER
Antibodies		
Polyclonal Guinea Pig Anti-Insulin	Dako	Cat#A0564; RRID: AB_10013624
Anti-Glucagon antibody	Abcam	Cat#ab10988; RRID: AB_659831
Human/Mouse Somatostatin Antibody	R&D Systems	Cat#MAB2358; RRID: AB_2722572
Pancreatic Polypeptide/PP Antibody	NOVUS Biologicals	Cat#NB100-1793; RRID: AB_2268669
Human Serpin A1/ alpha 1-Antitrypsin Antibody	R&D Systems	Cat#MAB1268; RRID: AB_2301508
Trypsin 1/PRSS1 Antibody	NOVUS Biologicals	Cat#AF3848; RRID: AB_2171706
Cytokeratin 19 antibody	Abcam	Cat# ab7754; RRID: AB_306048
Human CD31/PECAM-1 Antibody	R&D Systems	Cat#AF806; RRID: AB_355617
Vimentin antibody	Abcam	Cat#ab8978; RRID: AB_306907
NRP1 antibody	Abcam	Cat#ab81321; RRID: AB_1640739
NRP1 antibody	NOVUS Biologicals	Cat#ST05-30; RRID: AB_2809467
NRP1 antibody	R&D Systems	Cat#AF3870; RRID: AB_884367
G3BP1 Antibody	Cell Signaling Technology	Cat#17798; RRID: AB_2884888
SARS-CoV/SARS-CoV-2 Nucleocapsid Antibody	Sino Biological	Cat#40143-R001; RRID: AB_2827974
Human ACE-2 Antibody	R&D Systems	Cat#AF933; RRID: AB_355722
eIF2alpha Rabbit mAb	Cell Signaling	Cat #5324S; RRID: AB_10692650
Phospho-eIF2alpha (Ser51) Rabbit mAb	Cell Signaling	Cat #3398S; RRID: AB_2096481
PKR Rabbit mAb	Cell Signaling	Cat #12297S; RRID: AB_2665515
β-Actin Rabbit mAb	Cell Signaling	Cat #8457S; RRID: AB_10950489
Recombinant Anti-PKR (phospho T446) antibody	Abcam	Cat # ab32036; RRID: AB_777310
IRDye 800CW Donkey anti-Rabbit IgG Secondary Antibody	LI-COR	Cat #926-32213; RRID: AB_621848
Goat anti-Rabbit IgG (H+L) Cross-Adsorbed Secondary Antibody, Pacific Blue	Thermo Fisher Scientific	Cat #P-10994; RRID: AB_2539814
Donkey anti-Goat IgG (H+L) Cross-Adsorbed Secondary Antibody, Alexa Fluor 488	Thermo Fisher Scientific	Cat#A-11055; RRID: AB_2534102
Donkey anti-Rat IgG (H+L) Highly Cross-Adsorbed Secondary Antibody, Alexa Fluor 488	Thermo Fisher Scientific	Cat# A-21208; RRID: AB_2535794
Donkey anti-Sheep IgG (H+L) Cross-Adsorbed Secondary Antibody, Alexa Fluor 488	Thermo Fisher Scientific	Cat#A-11015; RRID: AB_141362
DAPI	Santa Cruz	Cat#sc-3598; CAS: 28718-90-3
Chemicals, peptides, and recombinant proteins		
CHIR99021	Cayman Chemical	Cat#13122; CAS: 252917-06-9
Retinoic acid	Sigma Aldrich	Cat#R2625; CAS:302-79-4
DAPT	Sigma Aldrich	Cat#D5942; CAS: 208255-80-5
Exendin-4	Sigma Aldrich	Cat#E7144; CAS: 141758-74-9
Cyclopamine-KAAD	Sigma Aldrich	Cat#239804; CAS: 306387-90-6
trans-ISRIB	Tocris	Cat#5284; CAS: 1597403-47-8
Critical commercial assays		
Chromium Controller & Next GEM Accessory Kit	10x Genmoics	1000202
Chromium Next GEM Chip G Single Cell Kit	10x Genmoics	1000120
Chromium Next GEM Single Cell 5' Library and Gel Bead Kit v1.1, 16 rxns	10x Genmoics	1000165
Single Index Kit T Set A	10x Genomics	1000213
Culture medium		

(*Continued on next page*)

Continued

REAGENT or RESOURCE	SOURCE	IDENTIFIER
StemFlex	Gibco Thermo Fisher	Cat#A3349401
RPMI 1640	Corning	Cat#10-040-CMR
DMEM	Corning	Cat#MT10013CV
CMRL	Thermo Fisher Scientific	Cat#11530037
Glutamax Supplement	Thermo Fisher Scientific	Cat#35050079
B-27 Supplement	Thermo Fisher Scientific	Cat#17504044
Accutase	Stemcell Technologies	Cat# 07920
Matrigel	Corning	Cat#354234
**Deposited data**		
scRNA-seq	GSE159556	GEO: GSE159556
Code for data analysis	R scran package (v.1.14.1)	https://github.com/shuibingchen/COVID-19_Islets
**Experimental models: Cell lines**		
hESC line MEL-1	Monash University	RRID: CVCL_XA16
Vero E6	ATCC	Cat# CRL-1586; RRID: CVCL_0574
EndoC-betaH1	Cellosaurus	RRID: CVCL_L909
**Growth factors**		
Activin A	R&D Systems	Cat#338-AC-500/CF
IGF-1	Peprotech	Cat#100-11
HGF	Peprotech	Cat#100-39H
FGF10	Peprotech	Cat#100-26
**Software and algorithms**		
Cell Ranger v3.0.2	10× Genomics	https://support.10xgenomics.com/single-cell-gene-expression/software/overview/welcome; RRID: SCR_017344
Scran v1.14.1	Bioconductor	https://bioconductor.org/packages/release/bioc/html/scran.html; RRID: SCR_016944
batchelor v1.2.1	Bioconductor	http://bioconductor.org/packages/release/bioc/html/batchelor.html; RRID: SCR_003470
Seurat v3.1.0	Satijalab	https://satijalab.org/seurat/; RRID: SCR_007322
ggplot2 v3.2.1	Tidyverse	https://ggplot2.tidyverse.org/index.html; RRID: SCR_014601
pheatmap v1.0.12	Raivo Kolde	https://cran.r-project.org/package=pheatmap; RRID: SCR_016418
MetaMoph	Molecular Devices	http://www.moleculardevices.com/Products/Software/Meta-Imaging-Series/MetaMorph.html; RRID: SCR_002368
DAVID6.8	LHRI	https://david.ncifcrf.gov/home.jsp; RRID: SCR_001881
Adobe illustrator CC2017	Adobe	https://www.adobe.com/product/photoshop.html; RRID: SCR_010279
Graphpad Prism 6	Graphpad software	https://www.graphpad.com; RRID: SCR_002798
ToppCell Atlas	Toppgene	https://toppgene.cchmc.org/; RRID: SCR_005726

RESOURCE AVAILABILITY

Lead contact

Further information and requests for resources and reagents should be directed to and will be fulfilled by the Lead Contact, Shuibing Chen (shc2034@med.cornell.edu)

Materials availability

This study did not generate new unique reagents.

Data and code availability

scRNA-seq data is available from the GEO repository database with accession number GEO: GSE159556. The codes used for data analysis and figure generation are available at https://github.com/shuibingchen/COVID-19_Islets.

EXPERIMENTAL MODEL AND SUBJECT DETAILS

Cell lines

Vero E6 (African green monkey [Chlorocebus aethiops] kidney, CVCL_0574; female) were obtained from ATCC (https://www.atcc.org/). Cells were cultured in Dulbecco's Modified Eagle Medium (DMEM) supplemented with 10% FBS and 100 U/mL penicillin and 100 μg/mL streptomycin. EndoC-betaH1 cells (CVCL_L909; female)were obtained from Cellosaurus, and cultured in DMEM containing 5.6 mM glucose, 2% BSA fraction V(Sigma), 50 μM 2-mercaptoethanol, 10 mM nicotinamide (Calbiochem), 5.5 μg/ml transferrin (Sigma-Aldrich), 6.7 ng/ml selenite (Sigma-Aldrich), 100 U/ml penicillin and 100 μg/ml streptomycin. $INS^{GFP/W}$ MEL-1 cells (CVCL_XA16; male) were provided by Dr. Ed Stanley at Monash University, Australia. Cells were cultured on Matrigel-coated 6-well plates in StemFlex medium (Thermo Fisher). All cells were maintained at 37°C with 5% CO2.

Human pancreatic islets

The pancreatic organs were obtained from the local organ procurement organization under the United Network for Organ Sharing (UNOS). The informed consent was obtained for research purposes. The islets were isolated in the Human Islet Core at University of Pennsylvania following the guidelines of Clinical Islet Transplantation consortium protocol (Ricordi et al., 2016). Briefly, the pancreas was digested following intraductal injection of Collagenase & Neutral Protease in Hanks' balanced salt solution. Liberated islets were then purified on continuous density gradients (Cellgro, Mediatech) using the COBE 2991 centrifuge and cultured in CIT culture media and kept in a humidified 5% CO_2 incubator.

Purified human pancreatic islets for scRNA-seq in Table S3 were obtained from Prodo Laboratories (Aliso Viejo, CA) and the National Disease Research Interchange (Philadelphia, PA), were isolated from cadaverous donors. All experimental protocols performed for this study are approved under the National Institutes of Health (NIH) guidelines. Purified islets were cultured in Prodo Islet Media [PIM(S)] complete media at a density of 10,000 Islet Equivalents (IEQ) per 150mm^2 for 72 hrs at 37°C. Islets were transported to the laboratory at 4°C over a period of 24 hrs. Upon receipt, islets were equilibrated to 37°C for 1 hour or overnight prior to processing for downstream scRNA-seq.

The gender and age information of islet donors has been provided as Table S1. The sample size is not large enough to analyze the influence of sex or gender on the results of the studies.

Human subjects

Tissue was acquired from deceased COVID-19 human donors, provided by the Weill Cornell Department of Pathology. The uninfected human pancreatic samples were similarly obtained. The Tissue Procurement Facility operates under Institutional Review Board (IRB) approved protocol and follows guidelines set by HIPAA. Experiments using samples from human subjects were conducted in accordance with local regulations and with the approval of the institutional review board at the Weill Cornell Medicine under protocol 20-04021814. Autopsy consent was obtained from the families of the patients.

The gender and age information of subjects has been provided as Table S1. The sample size was determined by availability. The sample size is not large enough to analyze the influence of sex or gender on the results of the studies.

METHOD DETAILS

All studies were performed in a blinded manner without inclusion and exclusion applied. The sample size and statistical analysis method of each experiments have been provided in the figure legends.

SARS-CoV-2 propagation and infection

SARS-CoV-2 isolate USA-WA1/2020 (NR-52281) was provided by the Center for Disease Control and Prevention and obtained through BEI Resources, NIAID, NIH. SARS-CoV-2 was propagated in Vero E6 cells in DMEM supplemented with 2% FBS, 4.5 g/L D-glucose, 4 mM L-glutamine, 10 mM Non-essential amino acids, 1 mM sodium pyruvate and 10 mM HEPES using a passage-2 stock of virus. Three days after infection, supernatant containing propagated virus was filtered through an Amicon Ultra 15 (100 kDa) centrifugal filter (Millipore Sigma) at ~4000 rpm for 20 minutes. Flow through was discarded and virus was resuspended in DMEM supplemented as described above. Infectious titers of SARS-CoV-2 were determined by plaque assay in Vero E6 cells in Minimum Essential Media supplemented with 2% FBS, 4 mM L-glutamine, 0.2% BSA, 10 mM HEPES and 0.12% $NaHCO_3$ and 0.7% agar. All MOIs were based on titer determined from plaque assays on Vero E6 cells. All work involving live SARS-CoV-2 was performed in the CDC and USDA-approved BSL-3 facility of the Icahn School of Medicine at Mount Sinai in accordance with institutional biosafety requirements.

scRNA-seq of viral infections

Approximately 1×10^6 untreated, control or *trans*-ISRIB treated primary human pancreatic islet cells were infected with passage-3 SARS-CoV-2 at an MOI of 1 for 24 hrs in CIT culture media. After 24 hrs, infected and mock infected pancreatic islets were

dissociated into a single cell suspension using Accutase cell detachment solution (Innovative Cell Technologies) and incubated at 37 °C for ~20 minutes followed by gentle pipetting to break apart groups of cells. Cells were then washed twice in 1x PBS and filtered using a 40 μm Flowmi cell strainer (Bel-Art Scienceware). Cell count and cell viability were then determined using trypan blue staining and a Countess II automatic cell counter (ThermoFisher Scientific). Target cell inputs of 10,000 cells for each condition were then loaded into a Chromium Controller using Chromium Next GEM (Gel Bead-In Emulsion) single Cell 5' Library & Gel Bead Kit v1.1 (10× Genomics) according to manufacturer's instructions. After generation of GEMs, cDNA synthesis and library preparation of all samples was completed using the Chromium Single Cell 5' Library Kit v1.1 (10× Genomics) according to manufacturer's instructions.

Sequencing and gene expression UMI counts matrix generation

The 10× Libraries were sequenced on the Illumina NovaSeq6000 sequencer with pair-end reads (28 bp for read 1 and 91 bp for read 2). The sequencing data were primarily analyzed by the 10× cellranger pipeline (v3.0.2) in two steps. In the first step, cellranger *mkfastq* demultiplexed samples and generated fastq files; and in the second step, cellranger count aligned fastq files to the reference genome and extracted gene expression UMI counts matrix. In order to measure both human and viral gene expression, we built a custom reference genome by integrating the SARS-CoV-2 virus genome into the 10× pre-built human reference (GRCh38 v3.0.0) using cellranger *mkref*. The SARS-CoV-2 virus genome (NC_045512.2) was downloaded from NCBI.

Single-cell RNA-seq data analysis

We filtered cells with less than 500 or more than 6000 genes detected, cells with less than 1000 or more than 60000 UMIs detected, as well as cells with mitochondria gene content greater than 15%, and used the remaining cells (3190 cells for non-COVID-19_5_Mock; 4936 cells for non-COVID-19_5_SARS-CoV-2; 8109 cells for non-COVID-19_6_Mock; 7559 cells for non-COVID-19_6_SARS-CoV-2; 8484 cells for non-COVID-19_6_SARS-CoV-2_*trans*-ISIRB) for downstream analysis.

We normalized the gene expression UMI counts using a deconvolution strategy implemented by the R scran package (v.1.14.1). In particular, we pre-clustered cells using the *quickCluster* function; we computed size factor per cell within each cluster and rescaled the size factors by normalization between clusters using the *computeSumFactors* function; and we normalized the UMI counts per cell by the size factors and took a logarithm transform using the *normalize* function. We further normalized the UMI counts across samples using the *multiBatchNorm* function in the R batchelor package (v1.2.1).

We identified highly variable genes using the *FindVariableFeatures* function in the R Seurat package (v3.1.0) , and selected the top 3000 variable genes after excluding mitochondria genes, ribosomal genes, viral genes and dissociation-related genes. The list of dissociation-related genes was originally built on mouse data ; we converted them to human ortholog genes using Ensembl BioMart. We aligned the five samples based on their mutual nearest neighbors (MNNs) using the *fastMNN* function in the R batchelor package, this was done by performing a principal component analysis (PCA) on the highly variable genes and then correcting the principal components (PCs) according to their MNNs. We selected the corrected top 50 PCs for downstream visualization and clustering analysis.

We ran Uniform Manifold Approximation and Projection (UMAP) dimensional reduction using the *RunUMAP* function in the R Seurat package with the number of neighboring points setting to 30 and training epochs setting to 4000. We clustered cells into twenty clusters by constructing a shared nearest neighbor graph and then grouping cells of similar transcriptome profiles using the *FindNeighbors* function and *FindClusters* function (resolution set to 0.4) in the R Seurat package. We identified marker genes for each cluster by performing differential expression analysis between cells inside and outside that cluster using the *FindMarkers* function in the R Seurat package. After reviewing the clusters, we merged them into nine clusters representing acinar cells, alpha cells, beta cells, ductal cells, fibroblast cells, delta cells, PP cells, endothelial cells and immune cells, for further analysis. We re-identified marker genes for the merged nine clusters and selected top 10 positive marker genes per cluster for heatmap plot using the R pheatmap package.

We generated UMAP and violin plots highlighting expressions of selected genes using the R ggplot2 package. We presented the difference in gene expressions between mock and SARS-CoV-2 infected conditions, and between DMSO and *trans*-ISRIB conditions by dot blot, where the size of a dot indicates the percentage of cells that express a gene and the color presents the relative expression level of a gene. The relative expression was calculated by taking mean of non-zero expressions of a gene across all cells within each condition and then linearly rescaling the two means such that they fall in range [-1, 1] by dividing their means and subtracting one. We generated a bar plot to present the expression changes in different cell types between mock and SARS-CoV-2 infected conditions. This was done by selecting cells with normalized expression greater or equal than 1.7, in order to mitigate possible background noise introduced by ambient RNA contaminations, and then calculating the mean expression over these cells per cluster per condition.

We compared the gene expression levels in beta cells between mock and SARS-CoV-2 infected conditions and identified differentially expressed genes based on Wilcoxon rank-sum test using the *FindMarkers* function in the R Seurat package. We highlighted differentially expressed genes with volcano plot and scatter plot. To generate the scatter plot, we calculated a score per gene per condition by multiplying the percentage of cells expressing the gene by the mean of non-zero expressions of the gene over all cells in the given condition, and showing the score in the two conditions (x axis for Mock and y axis for SARS-CoV-2 infected). We further performed pathway analysis on genes with p value less than 0.1 using QIAGEN Ingenuity Pathways Analysis (IPA).

To recover the transition during beta cell transdifferentiation, we performed trajectory analysis on beta cells from mock and SARS-CoV-2 conditions using R Slingshot package v1.4.0. We extracted beta cells from mock and SARS-CoV-2 infected samples by sub-setting the Seurat object using *subset* function in the R Seurat package. We identified highly variable genes using the

FindVariableFeatures function in the R Seurat package, and selected the top 3000 variable genes after excluding mitochondria genes, ribosomal genes, viral genes and dissociation-related genes. We aligned the four samples based on their mutual nearest neighbors (MNNs) using the *fastMNN* function in the R batchelor package. We selected the corrected top 50 PCs to perform UMAP dimensional reduction using the *RunUMAP* function in the R Seurat package with the number of neighboring points setting to 30 and training epochs setting to 500. We clustered cells into six clusters using the *FindNeighbors* function and *FindClusters* function (resolution set to 0.2) in the R Seurat package. We removed from the analysis a tiny set of likely-mislabeled cells that were far away from the beta cell clusters in the UMAP plot. We performed trajectory inference using function *slingshot* by setting UMAP as reduced dimensional data, cluster with low or high number of SARS-CoV-2 infected cells as the root and leaf node. We then identified genes that change their expression over the trajectory pseudo-time by fitting a general additive model (GAM) using R package gam v1.20. P-values were adjusted for multiple comparisons with Benjamini & Hochberg methods using R function *p.adjust*. We performed pathway analysis on 578 differentially expressed genes with adjusted p value less than 1.0E-100 using QIAGEN Ingenuity Pathways Analysis (IPA). We generated heatmap by sorting cells according to pseudo-time using the R pheatmap package. We generated scatter plot presenting the gene expression per cell as a function of pseudo-time, in which we fitted a line showing the transition trends using R function *loess* with span=1.

To further investigate into beta cells, we ran a sub-clustering analysis on these cells. In particular, we identified highly variable genes in beta cells using the *FindVariableFeatures* function in the R Seurat package (Stuart et al., 2019a), and selected the top 3000 variable genes after excluding mitochondria genes, ribosomal genes, viral genes and dissociation-related genes. We aligned these beta cells from the five samples based on their MNNs using the *fastMNN* function in the R batchelor package. We selected the corrected top 50 PCs for downstream sub-clustering analysis. We ran UMAP dimensional reduction using the *RunUMAP* function in the R Seurat package with the number of neighboring points setting to 35 and training epochs setting to 1000. We clustered cells into five clusters using the *FindNeighbors* function and *FindClusters* function (resolution set to 0.1) in the R Seurat package. We merged these clusters into two representing cell groups of relatively low or high GCG expression, for further analysis. We presented the difference in gene expressions in each sub-cluster between mock and SARS-CoV-2 infected conditions, and between DMSO and *trans*-ISRIB conditions by dot blot.

Immunofluorescence staining and confocal microscopy

Human islets were fixed with 4% paraformaldehyde at 4 °C overnight and transferred to 30% sucrose solution for dehydration. The islets were embedded in O.C.T (Fisher Scientific, Pittsburgh, PA) and serial 5 μm sections were taken with Leica cryostat microtome. The slides were blocked and permeabilized in PBS containing 5% horse serum and 0.1% Triton X-100 for 1 hour at room temperature and then incubated with primary antibodies at 4 °C overnight followed by incubation with fluorescence-conjugated secondary antibodies at room temperature for 1 hour. Nuclei were counterstained by DAPI. Slides were mounted in Prolong Gold Antifade Mountant (Thermo Fisher). The information of antibodies used for immunofluorescence staining are provided in Table S4. Images were taken by Zeiss LSM 800 confocal microscope and were scored using MetaMorph image analysis software (Molecular Devices).

Insulin secretion assays

Human islets were seeded into transwells and infected at an MOI of 1 for 48 hrs (Non-COVID-19_6, Non-COVID-19_7) or 72 hrs (Non-COVID-19_8). To perform glucose stimulated insulin secretion (GSIS) and KCl stimulated insulin secretion (KSIS) after infection, supernatant was removed and cells were starved in Krebs-Ringer Bicarbonate Buffer (KRBH) for 2 hrs at 37°C. After incubation, supernatant was removed and transwells containing islets were transferred into a new plate containing 500 uL of KRBH with 2.8 mM glucose (Non-COVID-19_6, Non-COVID-19_7) or 2 mM glucose (Non-COVID-19_8). An additional 100 uL of buffer was added to each transwell, and cells were incubated for 30 min at 37°C after which supernatant was collected and stored at -80°C. The same procedure was used sequentially with KRBH with 16.7 mM glucose (Non-COVID-19_6, Non-COVID-19_7) or 20 mM glucose (Non-COVID-19_8) followed by KRBH with 30 mM KCl. To measure the total level of insulin in samples, cells were lysed in RIPA buffer and stored at -80°C. 5% Triton X-100 was added to each sample to a final concentration of 0.5% to inactivate any remaining viral particles. Samples were then analyzed with a human insulin ELISA kit (Alpco), according to manufacturer's instructions.

Plaque assays

Human islets and Vero E6 cells were infected at an MOI of either 1.0 or 0.01. For 1.0 MOI infections, after an initial 1 hr incubation, supernatant was removed and cells were washed with fresh media 3 times to remove any remaining virus in supernatant. New media was added to islets and half of supernatant was collected and replaced with fresh media at each time point. For 0.01 MOI infections media was not replaced after addition of viral inoculate. Supernatant collected from each time point was frozen at -80°C once prior to determining titer. Infectious particle titer was determined by plaque assay on Vero E6 cells (ATCC #CRL-1586). $1x10^5$ cells were plated on 12-well plates and grown until confluent. 10-Fold serial dilutions of supernatant from each time point were prepared in triplicate in DMEM (Gibco) + 2% FBS. Vero E6 cells were inoculated with 200 μL of supernatant dilutions for 1 hr at 37°C with agitation every 10 mins. Inoculum was aspirated after 1hr and with an overlay of MEM (Gibco) with 4mM L-glutamine, 0.2% BSA, 10mM 703 HEPES, 0.12% NaHCO3, 2% FBS and 0.7% Oxoid agar and incubated at 37°C for 48 hrs. After 48hrs incubation samples were fixed

by adding 4% PFA on top of overlay for 24 hrs at room temperature. Plaques were then stained with crystal violet solution (0.2% crystal violet w/v, 20% EtOH v/v) for 1hr. Titer (PFU/mL) at each time point for each sample was calculated using the number of plaques counted at each dilution and the inoculation volume.

OPP staining

Human islets were infected at an MOI of 1 for 48 hrs. After fixation, the cells were stained with Click-iT Plus OPP Protein Synthesis Assay Kits (Molecular Probe) and insulin antibody. The relative expression was quantified using MetaMorph Microscopy Automation and Image Analysis Software.

hESC maintenance and pancreatic endocrine cell differentiation

Pancreatic endocrine cell differentiation was performed using $INS^{GFP/W}$ MEL-1 cells. Cells were cultured on Matrigel-coated 6-well plates in StemFlex medium (Thermo Fisher) and maintained at 37°C with 5% CO_2. MEL-1 cells were differentiated using a previously reported strategy (D'Amour et al., 2006). Briefly, on day 0, cells were exposed to basal medium RPMI 1640 (Corning) supplemented with 1× glutamax (Thermo Fisher), 50 μg/mL normocin, 100 ng/mL Activin A (R&D systems), and 3 μM of CHIR99021 (Cayman Chemical) for 24 hrs. The medium was changed on day 1 to basal RPMI 1640 medium supplemented with 1× glutamax, 50 μg/mL normocin, 0.2% FBS (Corning), 100 ng/mL Activin A for 2 days. On day 3, the resulting definitive endoderm cells were cultured in RPMI 1640 medium supplemented with 50 ng/ml FGF10, 0.25 μM Cyclopamine-KAAD and 2% FBS for 2 days to generate primitive gut tube cells. On day 5, the cells were induced to differentiate to posterior foregut in DMEM medium supplemented with 2 μM all-*trans* retinoic acid, 0.25 μM Cyclopamine-KAAD, 50 ng/ml FGF10 and 1% B27 supplement for 2 days. On day 7, the cells were induced to differentiate to pancreatic endoderm and endocrine precursors in DMEM medium supplemented with 1 μM DAPT, 50 ng/ml exendin 4 and 1% B27 for 3 days. On day 10, the cells were induced to differentiate to insulin expressing cells in CMRL medium supplemented with 50 ng/ml exendin 4, 50 ng/ml IGF-1, 50 ng/ml and 1% B-27 for 5 days.

ELISA Analysis

Mock and SARS-CoV-2 infected (MOI=1) pancreatic islet cell supernatants were collected at 24hpi for cytokine and chemokine analysis. The following cytokines/chemokines were evaluated using multiplex ELISA: CCL3, interleukin 1 receptor antagonist (IL-1RA), CXCL1, CCL8, CXCL2, CCL4, CXCL5, Interleukin 1 beta (IL1B), and CCL2. All antibodies and cytokine standards were purchased as antibody pairs from R&D Systems (Minneapolis, Minnesota) or Peprotech (Rocky Hill, New Jersey). Sample supernatants were first diluted 1:2 in a standard luminex buffer containing PBS supplemented with .01% Tween-20, 20 mM TRIS-HCl, 1% naive goat serum and 1% naive mouse serum. Diluted samples or analytic standards were then incubated with magnetic cytokine specific beads overnight at 4°C. Following, the assay plate was washed three times with luminex wash buffer (PBS + 0.01% Tween-20 + 20mM Tris-HCL) utilizing a strong magnet and then co-incubated with a mastermix of biotinylated detection antibodies for each analyte (R&D systems or Peprotech) for 1 hour at room temperature, shaking. Again, samples were washed three times with luminex wash buffer, and samples were then incubated with SA-PE for 1 hour at room temperature, shaking. Lastly, the assay plate was washed 3 more times, and samples were fixed with a solution of 2% PFA in the luminex buffer. The assays were read on a MAGPIX platform after fixation. The median fluorescence intensity of these beads was recorded for each bead and was used for analysis using a custom R script and a 5P regression algorithm. The limit of detection was defined as the pg/ml value associated with 3*sd(Background)+Median(background) for each analysis.

High throughput chemical screening

To perform the high throughput small-molecule screening, hESC-derived endocrine cells were dissociated using Accutase and replated onto 804G-coated 384-well plates at 10,000 cells per well. Cells were treated at 10 μM with compounds from an in-house library. DMSO treatment was used as a negative control. After 4 days of culture, cells were fixed and stained with antibodies against INS (DAKO) and GCG (Abcam). Plates were analyzed using a Molecular Devices ImageXpress High-Content Analysis System. Compounds inducing lower percentage of GCG^+INS^+ cells in INS^+ cells compared to DMSO treated wells were selected as primary hits.

Z score was calculated as below.

$$Z\ score = \frac{the\ polyhormonal\ rate\ of\ chemical\ treated\ condition - mean\ of\ the\ polyhormonal\ rate}{STDEV\ of\ the\ polyhormonal\ rate}$$

Flow cytometry and intracellular FACS analysis

Flow cytometry intracellular staining was performed following the instruction of user manual of Fixation and Permeabilization Solution Kit (BD Biosciences). Briefly, hESC-derived endocrine cells were dissociated and resuspended in Fixation and Permeabilization solution for 20 minutes at 4°C, then washed twice in 1x Perm and Wash buffer. Fixed cells were incubated with primary antibody at 4°C for 1 hr in the dark, washed twice and then incubated with fluorescence-conjugated secondary antibody for 30 minutes at 4°C in the dark. Cells were washed twice prior to flow cytometry analysis with an Accuri C6 flow cytometry instrument and the data were processed using Flowjo v10 software. The information of antibodies used for flow cytometry are provided in Table S4.

Western blot

EndoC-betaH1 cells were infected with SARS-CoV-2 (MOI=1), and protein was extracted at 48 hpi in RIPA buffer (Sigma) supplemented with Protease and Phosphatase Inhibitor Cocktail (Thermo Fisher) before safe removal from the BSL-3 facility. Protein samples were loaded onto NuPAGE 4-12% Bis-Tris Protein Gels (Thermo Fisher), resolved by electrophoresis and transferred onto nitrocellulose membranes. Membranes were incubated with the following primary antibodies: rabbit monoclonal anti-beta-Actin (Cell Signaling, 8457S, 1:1,000), rabbit monoclonal anti-PKR (Cell Signaling, 12297S, 1:1,000), rabbit monoclonal anti-PKR (phosphor T446) (Abcam, ab32036, 1:1,000), rabbit monoclonal anti-eIF2alpha (Cell Signaling, 5324S, 1:1,000) and rabbit monoclonal anti-Phospho-eIF2alpha (Cell Signaling, 3398S, 1:1,000). Primary antibodies were detected by fluorophore-conjugated secondary donkey anti-rabbit (IRDye 800CW, 926-32213, 1:15,000) antibody.

qRT-PCR

Total RNA samples were prepared from formalin-fixed and paraffin-embedded (FFPE) autopsy pancreatic tissues followed by DNaseI treatment using manufacturer's instructions (Qiagen RNeasy FFPE kit Cat# 73604). To quantify viral replication, measured by the expression of sgRNA transcription of the viral N gene, viral N or S gene, two-step RT-qPCR was performed using LunaScript RT SuperMix Kit (E3010L) for c-DNA synthesis and Luna Universal qPCR Master Mix (NEB #M3003) for RT-qPCR. Quantitative real-time PCR reactions were performed on CFX384 Touch Real-Time PCR Detection System (BioRad). The sequences of primers are provided in Table S5. Analysis was performed and -delta CT were calculated and normalized to 18S.

QUANTIFICATION AND STATISTICAL ANALYSIS

For *in vitro* studies, n = 3 independent replicates or 3 individual subjects or donors were used for all experiments unless otherwise indicated. For human samples, there is a limited resource of autopsy samples of COVID-19 subjects. We used all samples available when experiments were performed. Data are shown as mean ± STDEV. For two-group data, we used a two-tailed unpaired Student's t test. For one independent variable data, we used one-way ANOVA. For multiple samples' comparison, we used two-way ANOVA coupled with one-way ANOVA analysis. Statistical analysis was performed using GraphPad Prism 6 software.

Cell Metabolism, Volume *33*

Supplemental information

SARS-CoV-2 infection induces beta cell transdifferentiation

Xuming Tang, Skyler Uhl, Tuo Zhang, Dongxiang Xue, Bo Li, J. Jeya Vandana, Joshua A. Acklin, Lori L. Bonnycastle, Narisu Narisu, Michael R. Erdos, Yaron Bram, Vasuretha Chandar, Angie Chi Nok Chong, Lauretta A. Lacko, Zaw Min, Jean K. Lim, Alain C. Borczuk, Jenny Xiang, Ali Naji, Francis S. Collins, Todd Evans, Chengyang Liu, Benjamin R. tenOever, Robert E. Schwartz, and Shuibing Chen

SUPPLEMENTAL INFORMATION

Supplemental Figure S1-S7 and Tables S1-S5.

Figure S1

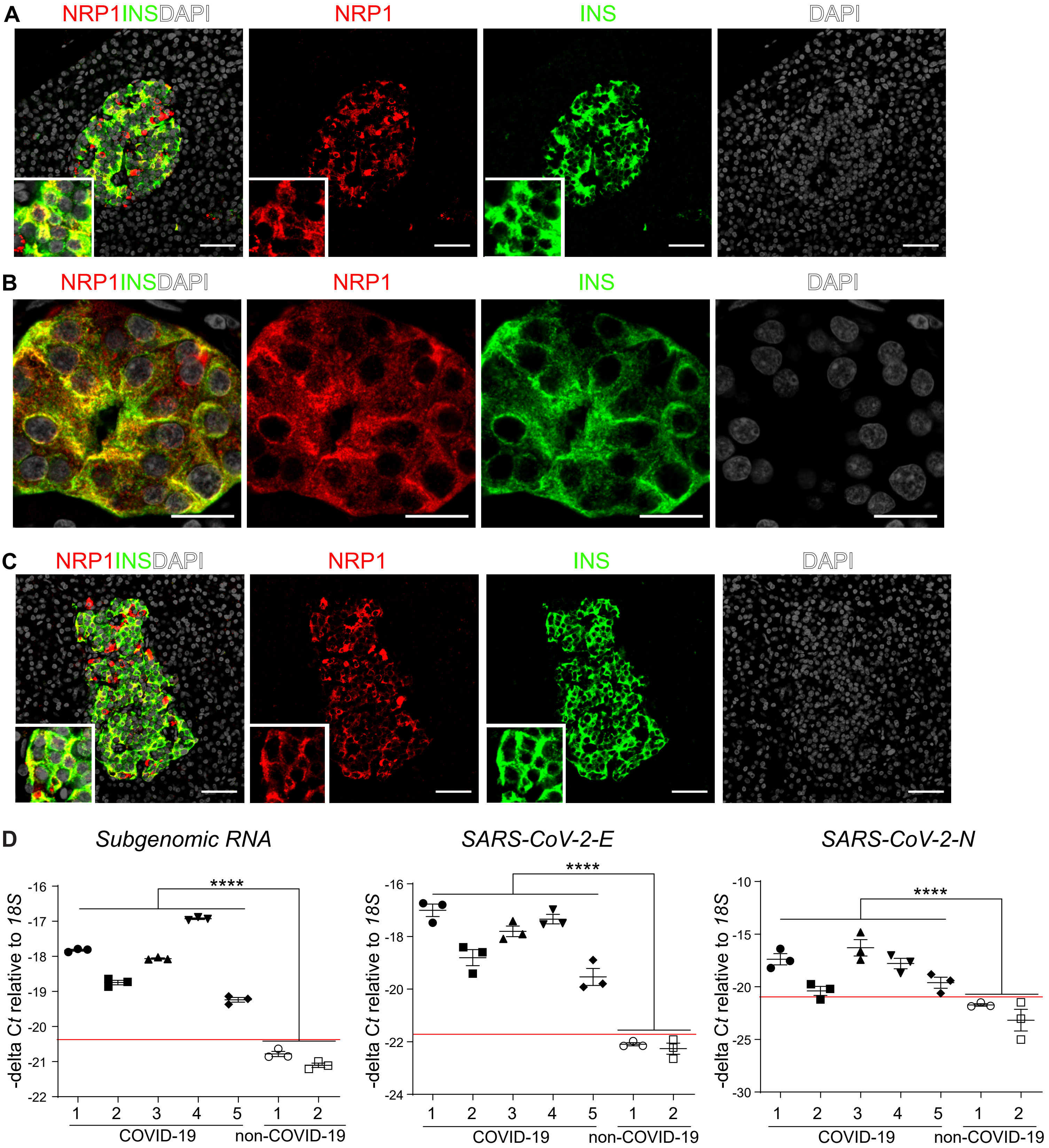
A
NRP1INSDAPI
NRP1
INS
DAPI
B
NRP1INSDAPI
NRP1
INS
DAPI
C
NRP1INSDAPI
NRP1
INS
DAPI
D
Subgenomic RNA
SARS-CoV-2-E
SARS-CoV-2-N
-delta Ct relative to 18S
****
-16
-17
-18
-19
-20
-21
-22
-24
-10
-15
-25
-30
1
2
3
4
5
COVID-19
non-COVID-19

SUPPLEMENTAL FIGURES.

Figure S1. Related to Figure 1 and 2. Immunostaining and qRT-PCR analysis of the autopsy pancreas samples of a non-COVID-19 and COVID-19 subjects.

(A) A representative image of NRP1 expression in the autopsy pancreas sample of a non-COVID-19 subject. 20X confocal image using NRP1 antibody from abcam. The insert represents a high-resolution image from the larger field. Scale bar = 50 μm. (*n* = 6 images examined in total) Red: NRP1; Green: INS; Grey: DAPI.

(B) A representative image of NRP1 in the autopsy pancreas sample of a non-COVID-19 subject. 63X confocal image using NRP1 antibody from NOVUS. Scale bar = 20 μm. (*n* = 2 images examined in total) Red: NRP1; Green: INS; Grey: DAPI.

(C) A representative image of NRP1 in the autopsy pancreas sample of a non-COVID-19 subject. 20X confocal image using NRP1 antibody from NOVUS. The insert represents a high-resolution image from the larger field. Scale bar = 50 μm. (*n* = 6 images examined in total) Red: NRP1; Green: INS; Grey: DAPI.

(D) qRT-PCR analysis of *subgenomic RNA*, *SARS-CoV-2-E*, *SARS-CoV-2-N* in the autopsy pancreas samples of non-COVID-19 and COVID-19 subjects. -Delta C*t* = C*t* (*18S*)-C*t* (*subgenomic RNA*, *SARS-CoV-2-E*, or *SARS-CoV-2-N*). Red line highlights the detection limit. Data was presented as mean ± STDEV. *P* values were calculated by one-way ANOVA, ****$P < 0.0001$.

Figure S2

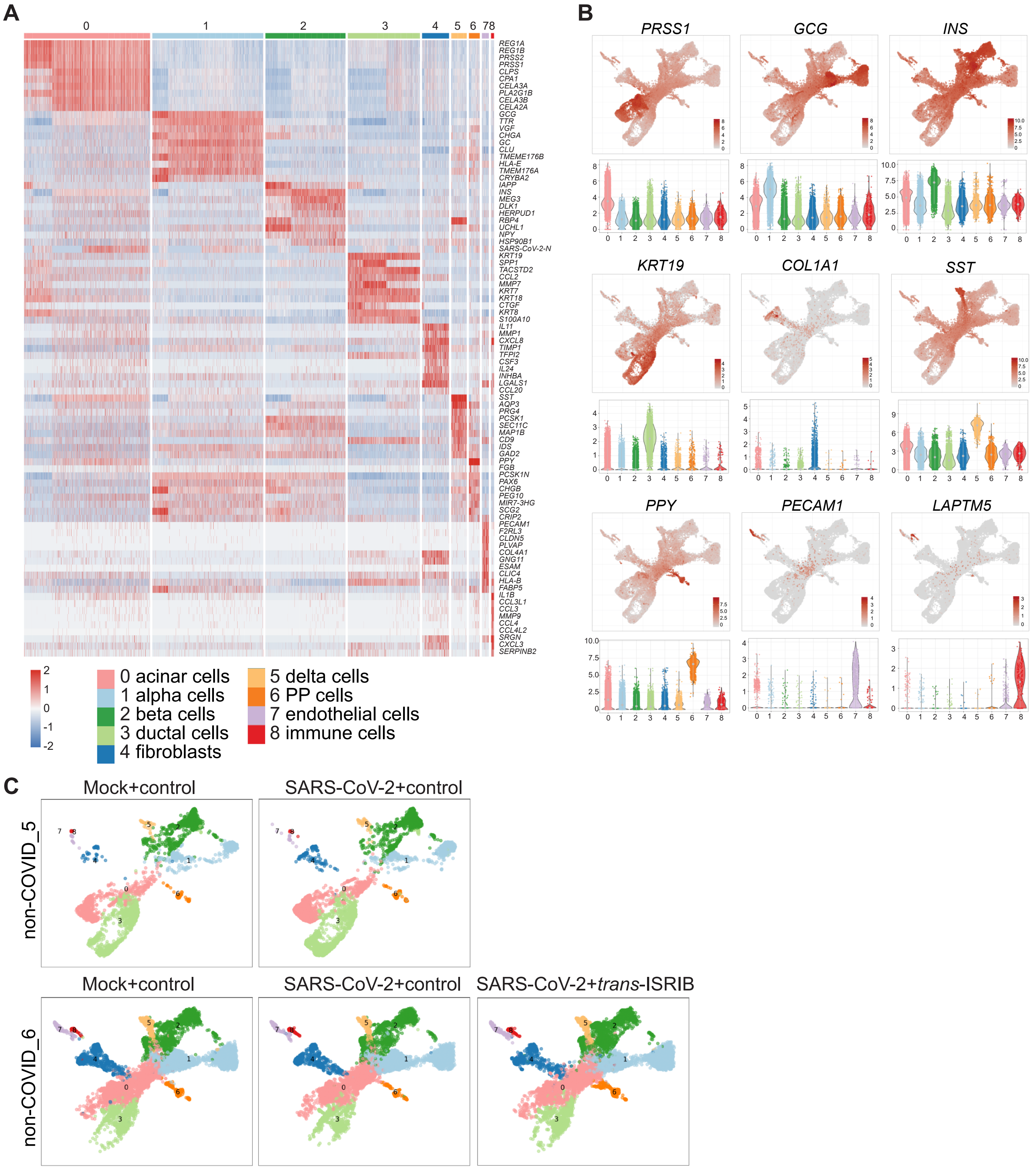
A
0 acinar cells
1 alpha cells
2 beta cells
3 ductal cells
4 fibroblasts
5 delta cells
6 PP cells
7 endothelial cells
8 immune cells
REG1A
REG1B
PRSS2
PRSS1
CLPS
CPA1
CELA3A
PLA2G1B
CELA3B
CELA2A
GCG
TTR
VGF
CHGA
GC
CLU
TMEME176B
HLA-E
TMEM176A
CRYBA2
IAPP
INS
MEG3
DLK1
HERPUD1
RBP4
UCHL1
NPY
HSP90B1
SARS-CoV-2-N
KRT19
SPP1
TACSTD2
CCL2
MMP7
KRT7
KRT18
CTGF
KRT8
S100A10
IL11
MMP1
CXCL8
TIMP1
TFPI2
CSF3
IL24
INHBA
LGALS1
CCL20
SST
AQP3
PRG4
PCSK1
SEC11C
MAP1B
CD9
IDS
GAD2
PPY
FGB
PCSK1N
PAX6
CHGB
PEG10
MIR7-3HG
SCG2
CRIP2
PECAM1
F2RL3
CLDN5
PLVAP
COL4A1
GNG11
ESAM
CLIC4
HLA-B
FABP5
IL1B
CCL3L1
CCL3
MMP9
CCL4
CCL4L2
SRGN
CXCL3
SERPINB2
B
PRSS1
GCG
INS
KRT19
COL1A1
SST
PPY
PECAM1
LAPTM5
C
Mock+control
SARS-CoV-2+control
SARS-CoV-2+trans-ISRIB
non-COVID_5
non-COVID_6

Figure S2. Related to Figure 2. Single-cell RNA-seq analysis of mock versus SARS-CoV-2 infected human islets.
(A) Heatmap showing relative expression of top 10 markers defining the nine cell types in human islets. (*n* = 2 individual islet donors).
(B) UMAP and violin plots showing expression levels of pancreatic cell markers. (*n* = 2 individual islet donors).
(C) UMAP of human islets by conditions.

Figure S3

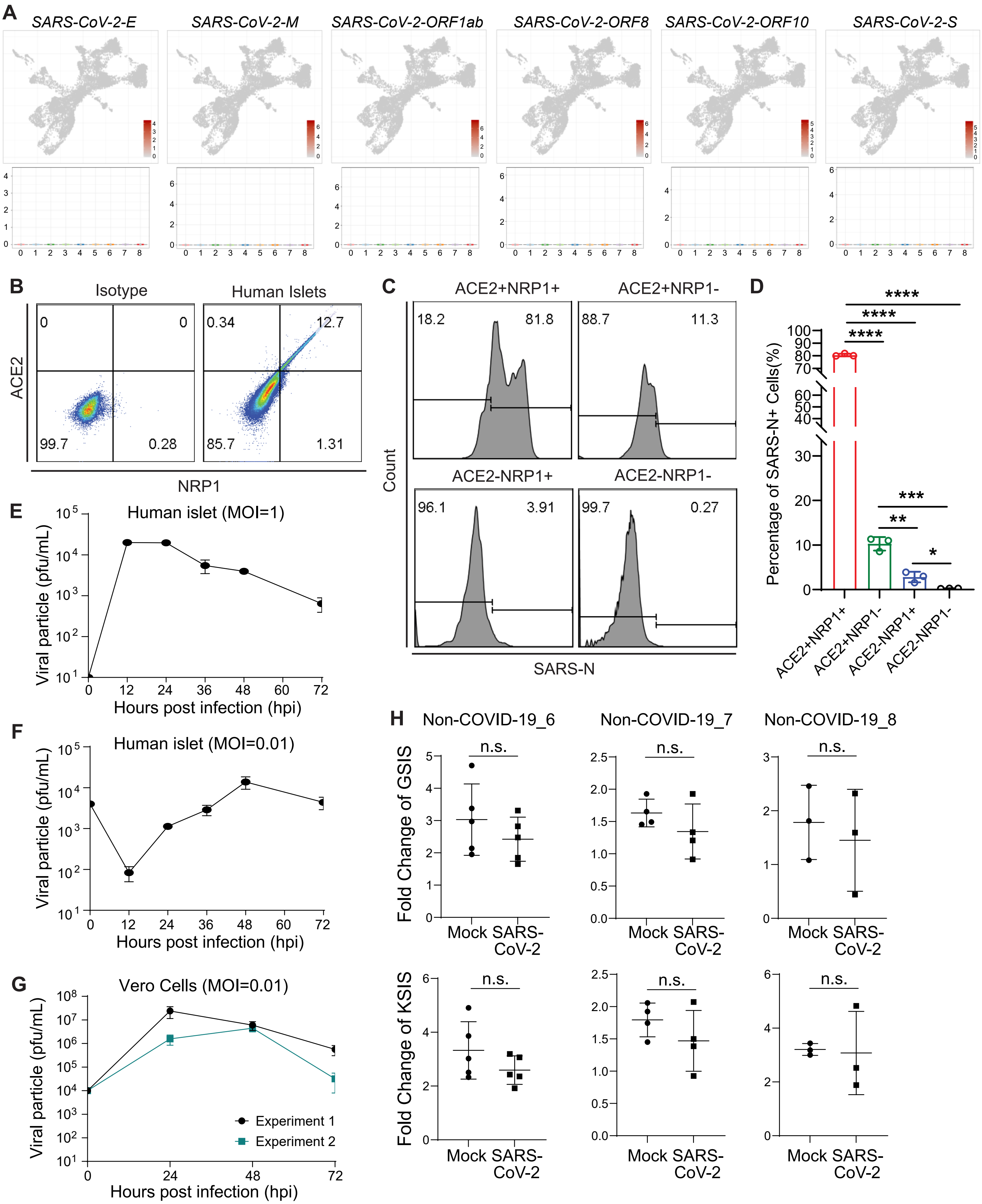
A
SARS-CoV-2-E
SARS-CoV-2-M
SARS-CoV-2-ORF1ab
SARS-CoV-2-ORF8
SARS-CoV-2-ORF10
SARS-CoV-2-S
B
Isotype
Human Islets
ACE2
NRP1
0
0
99.7
0.28
0.34
12.7
85.7
1.31
C
ACE2+NRP1+
ACE2+NRP1-
ACE2-NRP1+
ACE2-NRP1-
18.2
81.8
88.7
11.3
96.1
3.91
99.7
0.27
Count
SARS-N
D
Percentage of SARS-N+ Cells(%)
****
***
**
*
ACE2+NRP1+
ACE2+NRP1-
ACE2-NRP1+
ACE2-NRP1-
E
Human islet (MOI=1)
Viral particle (pfu/mL)
Hours post infection (hpi)
F
Human islet (MOI=0.01)
Viral particle (pfu/mL)
Hours post infection (hpi)
G
Vero Cells (MOI=0.01)
Viral particle (pfu/mL)
Experiment 1
Experiment 2
Hours post infection (hpi)
H
Non-COVID-19_6
Non-COVID-19_7
Non-COVID-19_8
n.s.
Fold Change of GSIS
Fold Change of KSIS
Mock
SARS-CoV-2

Figure S3. Related to Figure 2. SARS-CoV-2 infects multiple types of human pancreatic cells.
(A) UMAP and violin plots showing the expression levels of SARS-CoV-2 genes of mock infected human islets. (*n* = 2 individual islet donors).
(B and C) Flow cytometry analysis of SARS-CoV-2 (MOI=1) infected human islets at 48 hpi. Gating strategy of ACE2 and NRP1 (B). SARS-N expression in $ACE2^+/NRP1^+$, $ACE2^-/NRP1^+$, $ACE2^+/NRP1^-$, and $ACE2^-/NRP1^-$ cells (C). *n* = 3 replicates.
(D) Quantification of $SARS\text{-}N^+$ percentage in $ACE2^+/NRP1^+$, $ACE2^+/NRP1^-$, $ACE2^-/NRP1^+$, and $ACE2^-/NRP1^-$ cells.
(E) Plaque assays of SARS-CoV-2 (MOI=1) infected human islets at 12, 24, 36, 48, 60, and 72 hpi (*n* = 3 replicates per time point) with replacement of media and 3 times washes after initial 3 hr infection.
(F) Plaque assays of SARS-CoV-2 (MOI=0.01) infected human islets at 12, 24, 36, 48, 60 and 72 hpi (*n* = 3 replicates per time point).
(G) Plaque assays of SARS-CoV-2 (MOI=0.01) infected Vero cells at 24, 48, and 72 hpi (*n* = 3 replicates per time points from two individual experiments).
(H) Fold change of glucose stimulated insulin secretion (GSIS) or KCl stimulated insulin secretion (KSIS) of SARS-CoV-2 (MOI=1) infected human islets. Human islets from non-COVID-19_6 (*n* = 5 replicates per condition) and non-COVID-19_7 (*n* = 4 replicates per condition) were analyzed at 48 hpi. Human islets from non-COVID-19_8 (*n* = 3 replicates per condition) were analyzed at 72 hpi.
Data was presented as mean ± STDEV. *P* values were calculated by two-way ANOVA. $^*P < 0.05$, $^{**}P < 0.01$, $^{***}P < 0.001$ and $^{****}P < 0.0001$.

Figure S4

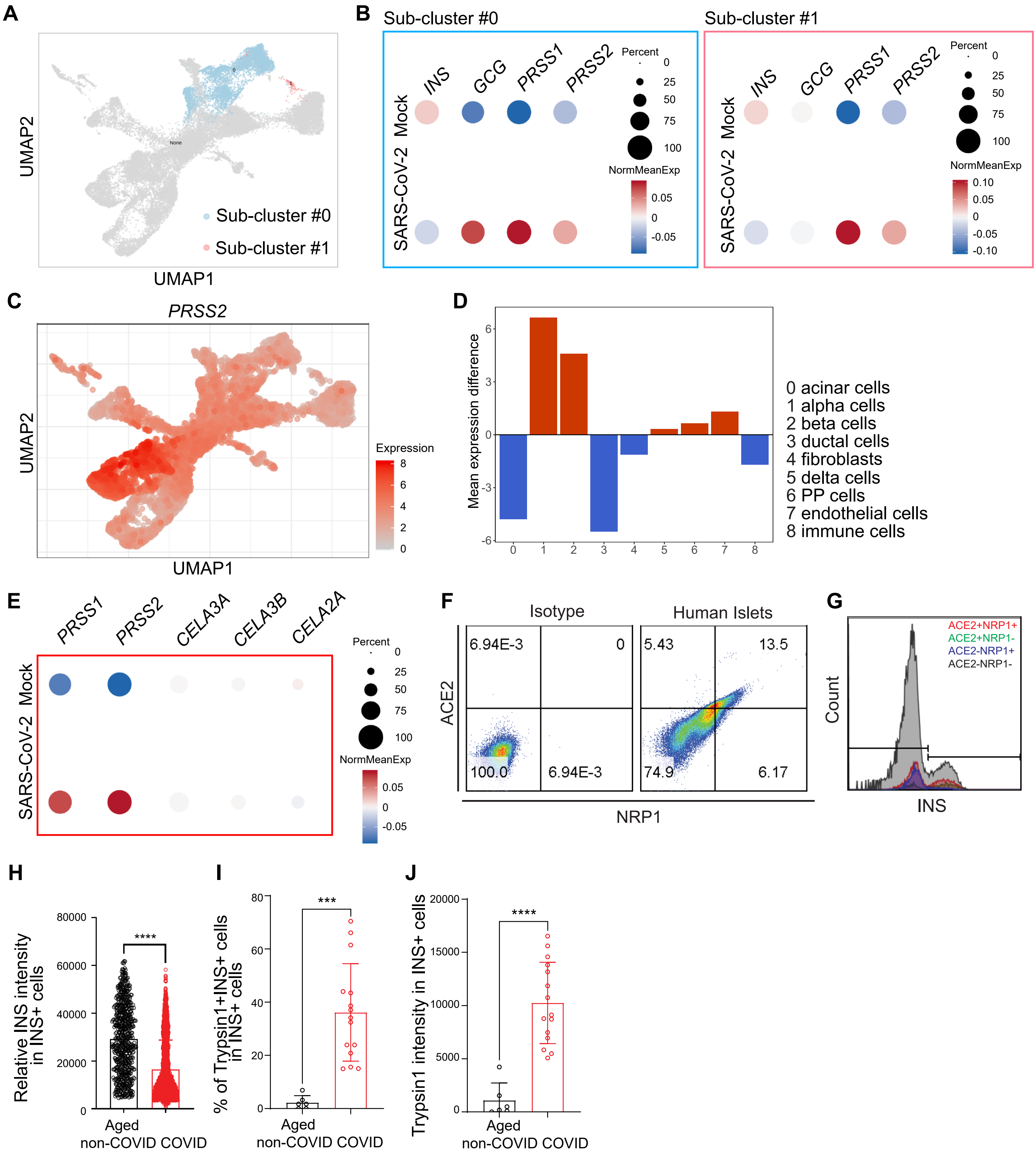

A
UMAP2
UMAP1
Sub-cluster #0
Sub-cluster #1
B
Sub-cluster #0
Sub-cluster #1
INS
GCG
PRSS1
PRSS2
SARS-CoV-2
Mock
Percent
0
25
50
75
100
NormMeanExp
0.10
0.05
0
-0.05
-0.10
C
PRSS2
UMAP2
UMAP1
Expression
8
6
4
2
0
D
Mean expression difference
0 acinar cells
1 alpha cells
2 beta cells
3 ductal cells
4 fibroblasts
5 delta cells
6 PP cells
7 endothelial cells
8 immune cells
E
PRSS1
PRSS2
CELA3A
CELA3B
CELA2A
SARS-CoV-2
Mock
F
Isotype
Human Islets
ACE2
NRP1
6.94E-3
0
100.0
6.94E-3
5.43
13.5
74.9
6.17
G
ACE2+NRP1+
ACE2+NRP1-
ACE2-NRP1+
ACE2-NRP1-
Count
INS
H
Relative INS intensity in INS+ cells
****
Aged non-COVID
COVID
I
% of Trypsin1+INS+ cells in INS+ cells
***
Aged non-COVID
COVID
J
Trypsin1 intensity in INS+ cells
****
Aged non-COVID
COVID

Figure S4. Related to Figure 4. Human beta cells undergo transdifferentiation upon SARS-CoV-2 infection.
(A) UMAP plot showing the two sub-clusters of beta cell population. (*n* = 2 individual islet donors).
(B) Dot blot illustrating expression level of *INS*, *GCG, PRSS1, PRSS2* in two sub-clusters of mock versus SARS-CoV-2-infected human islets at 24 hpi (MOI=1). Dot size shows the fraction of cells with non-zero expression; Dot color indicates the relative expression level in the two conditions. (*n* = 2 individual islet donors).
(C) UMAP plot showing the expression level of *PRSS2* of SARS-CoV-2 infected human islets. (*n* = 2 individual islet donors).
(D) Bar plot illustrating mean expression difference of *PRSS2* between mock and SARS-CoV-2 infected cells in different clusters of human islets; Bar color indicates increased (red) or decreased (blue) expression in the SARS-CoV-2 infected cells. (*n* = 2 individual islet donors).
(E) Dot blot illustrating expression level of acinar cell markers, including *PRSS1, PRSS2, CELA3A, CELA3B, CELA2A* in beta cell cluster of mock versus SARS-CoV-2-infected human islets at 24 hpi (MOI=1). Dot size shows the fraction of cells with non-zero expression; Dot color indicates the relative expression level in the two conditions. (*n* = 2 individual islet donors).
(F and G) Flow cytometry analysis of SARS-CoV-2 (MOI=1) infected human islets at 48 hpi. Gating strategy of ACE2 and NRP1 (F). Insulin expression in $ACE2^+/NRP1^+$, $ACE2^-/NRP1^+$, $ACE2^+/NRP1^-$, and $ACE2^-/NRP1^-$ cells (G). (*n* = 3 replicates).
(H-J) Quantification of the relative INS intensity in INS^+ cells (H), the percentage of $Typsin1^+INS^+$ cells in INS^+ cells (I) and the average Typsin1 intensity in INS^+ cells (J) of autopsy samples of COVID-19 subjects or age-matched non-COVID-19 subjects. Three images of each sample were used for quantification for each subject (*n* = 2 individual non-COVID-19 subjects; *n* = 5 individual COVID-19 subjects).
Data was presented as mean ± STDEV. *P* values were calculated by paired or unpaired two-tailed Student's t test. ***$P < 0.001$ and ****$P < 0.0001$.

Figure S5

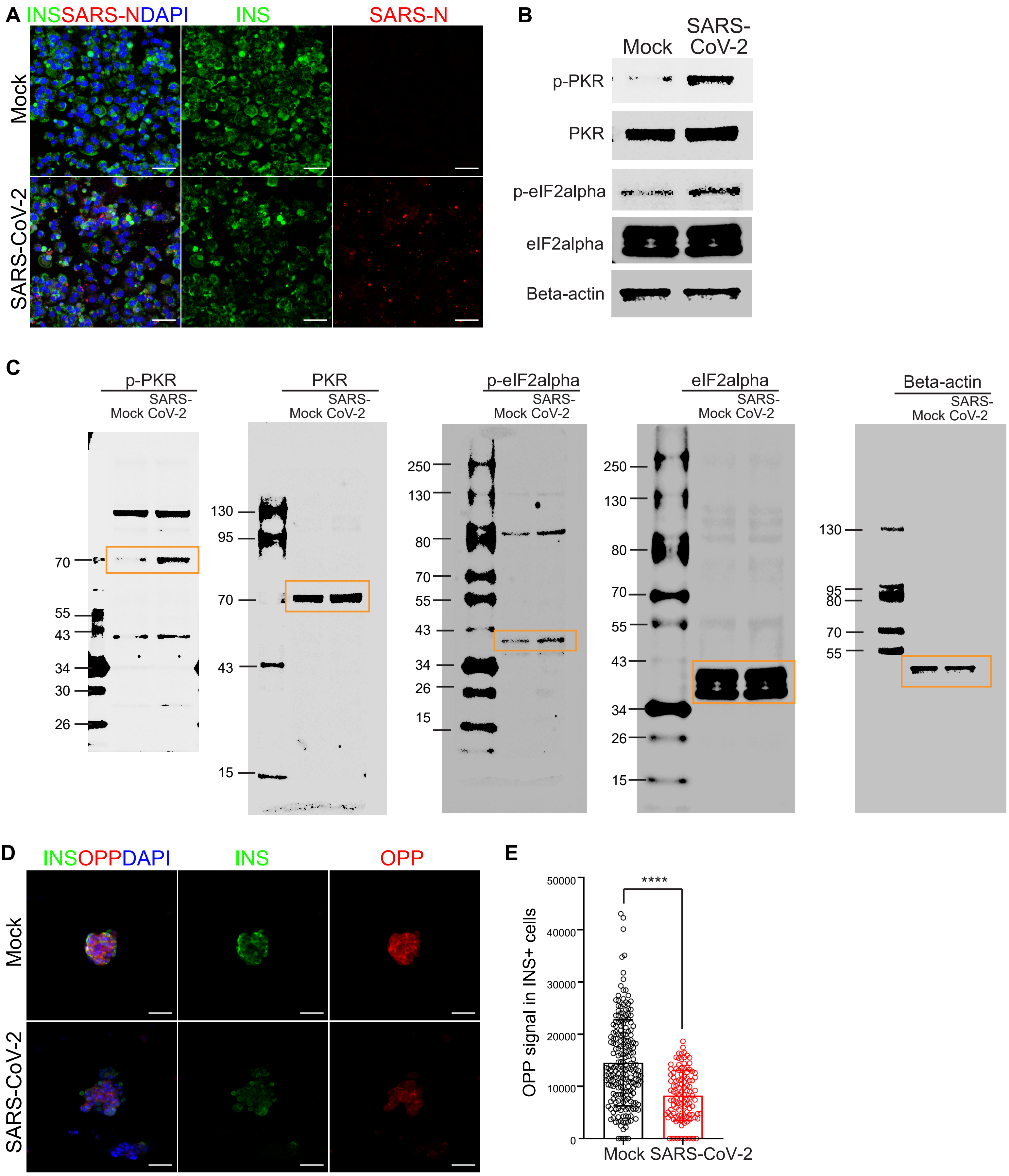
A
INSSARS-NDAPI
INS
SARS-N
Mock
SARS-CoV-2
B
Mock
SARS-CoV-2
p-PKR
PKR
p-eIF2alpha
eIF2alpha
Beta-actin
C
p-PKR
PKR
p-eIF2alpha
eIF2alpha
Beta-actin
SARS-
Mock CoV-2
D
INSOPPDAPI
INS
OPP
Mock
SARS-CoV-2
E
OPP signal in INS+ cells
****
Mock SARS-CoV-2

Figure S5. Related to Figure 4. SARS-CoV-2 infection causes the change of eIF2 pathway and the decrease of protein synthesis.

(A) Representative confocal images of INS and SARS-N of mock and SARS-CoV-2 (MOI=1) infected EndoC-betaH1 cells at 48 hpi (MOI=1). Scale bar = 50 μm. Red: SARS-N; Green: INS; Blue: DAPI.

(B) Western blotting of p-PKR, PKR, p-eIF2alpha, eIF2alpha and beta-actin of mock and SARS-CoV-2 (MOI=1) infected EndoC-betaH1 cells at 48 hpi (MOI=1).

(C) Whole gel images of western blotting at (B).

(D and E) Representative confocal images of INS and OPP (O-propargyl-puromycin) (D) and quantification of OPP staining intensity (E) of mock versus SARS-CoV-2 infected human islets at 48 hpi (n = 3 replicates, MOI=1). Scale bar = 50 μm. Red: OPP; Green: INS; Blue: DAPI.

Figure S6

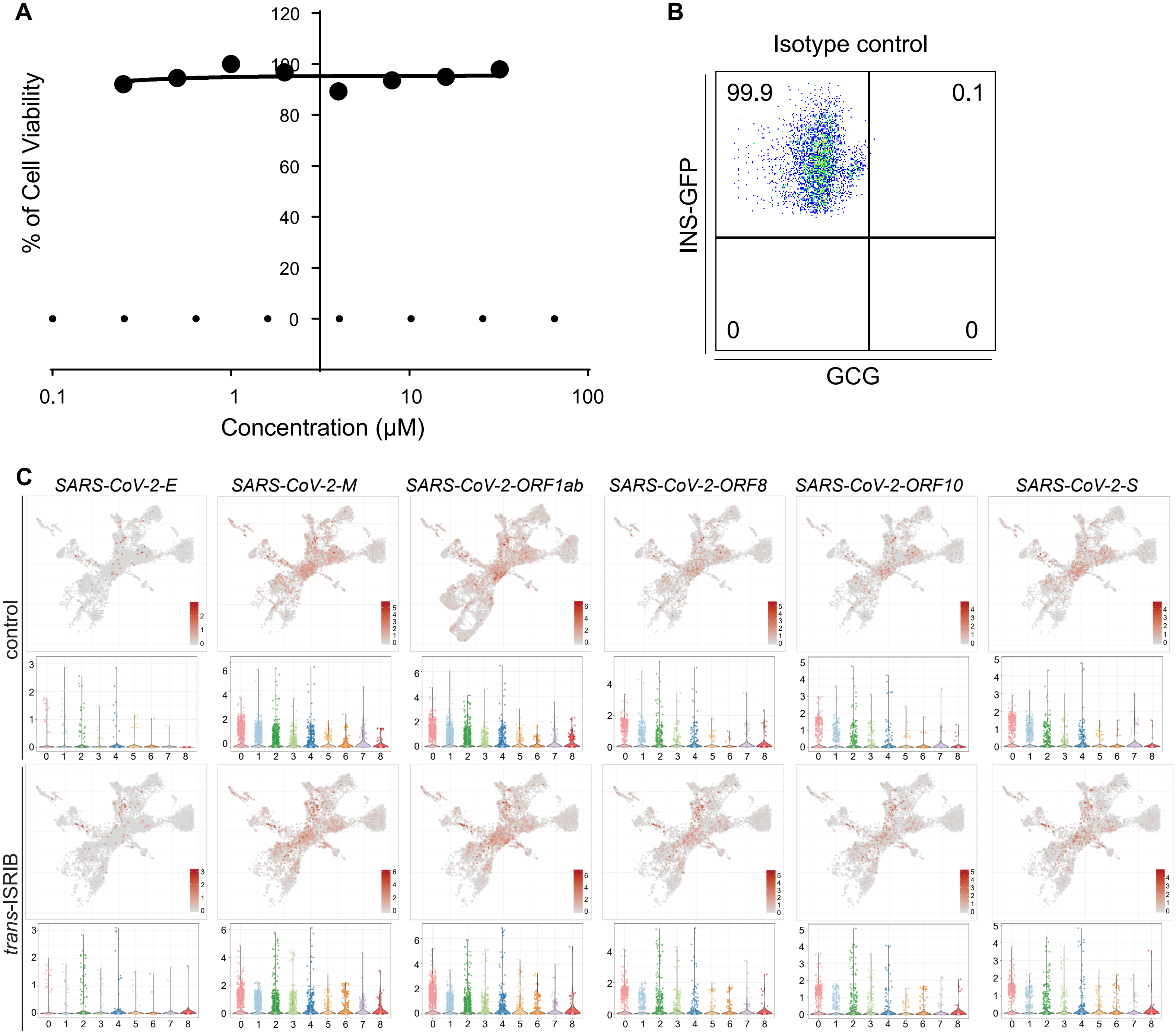
A
% of Cell Viability
120
100
80
60
40
20
0
0.1
1
10
100
Concentration (μM)
B
Isotype control
99.9
0.1
0
0
INS-GFP
GCG
C
SARS-CoV-2-E
SARS-CoV-2-M
SARS-CoV-2-ORF1ab
SARS-CoV-2-ORF8
SARS-CoV-2-ORF10
SARS-CoV-2-S
control
trans-ISRIB

Figure S6. Related to Figure 6 and 7. High-throughput chemical screen.
(A) Toxicity curve of *trans*-ISRIB on hESC-derived *INS*-GFP$^+$ beta-like cells. Data was presented as mean ± STDEV. (*n* = 3 biological replicates).
(B) Isotype control of flow cytometry analysis of *trans*-ISRIB treated hESC-derived *INS*-GFP$^+$ beta-like cells.
(C) UMAP and violin plots showing the expression levels of SARS-CoV-2 genes of control or 10 µM *trans*-ISRIB treated human islets at 24 hpi (*n* = 2 individual islet donors, MOI=1).

Figure S7

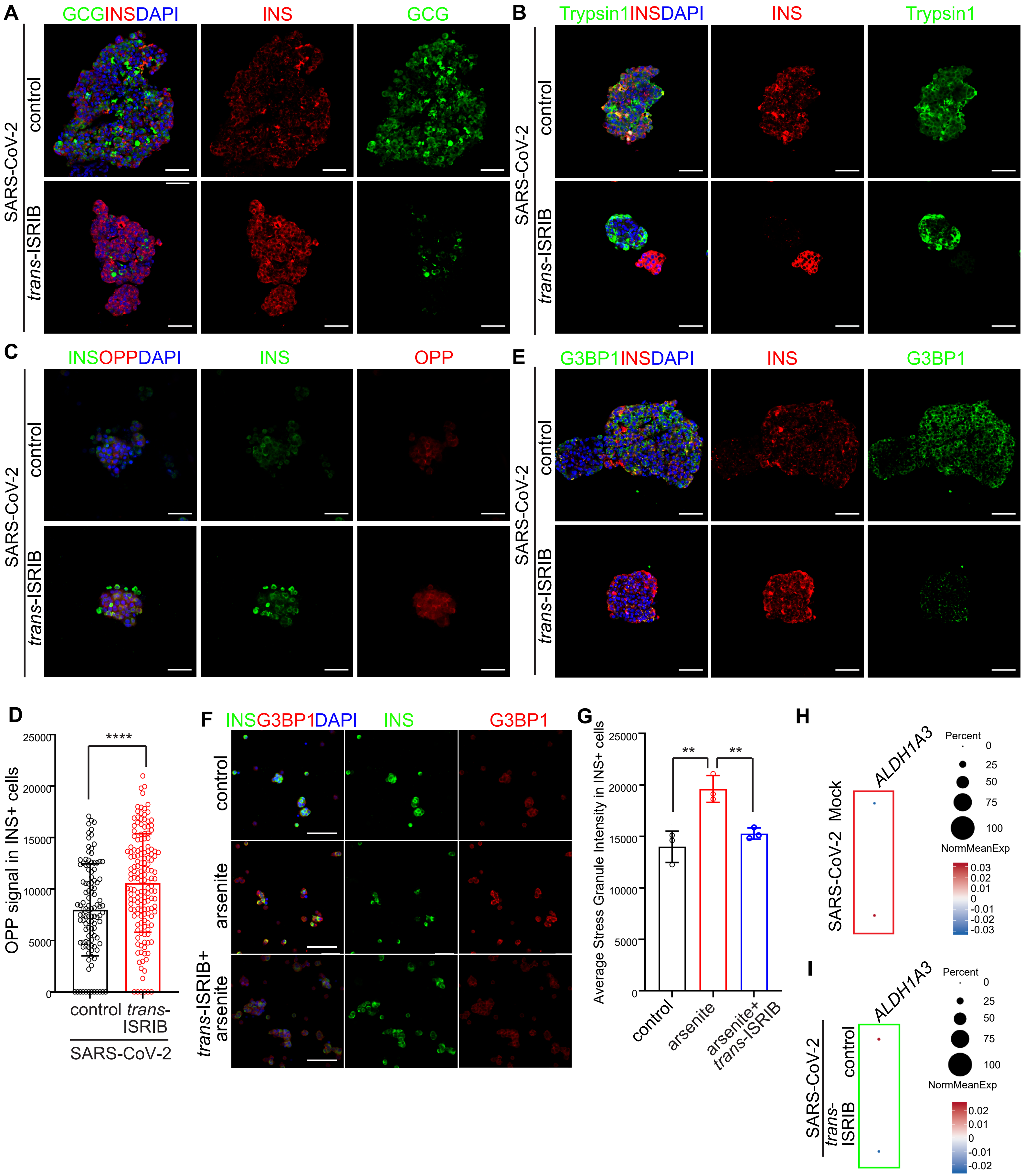

A
GCGINSDAPI
INS
GCG
SARS-CoV-2
control
trans-ISRIB
B
Trypsin1INSDAPI
INS
Trypsin1
C
INSOPPDAPI
INS
OPP
E
G3BP1INSDAPI
INS
G3BP1
D
OPP signal in INS+ cells
25000
20000
15000
10000
5000
0
****
control
trans-ISRIB
SARS-CoV-2
F
INSG3BP1DAPI
INS
G3BP1
control
arsenite
trans-ISRIB+ arsenite
G
Average Stress Granule Intensity in INS+ cells
**
**
control
arsenite
arsenite+ trans-ISRIB
H
ALDH1A3
SARS-CoV-2 Mock
Percent
0
25
50
75
100
NormMeanExp
0.03
0.02
0.01
0
-0.01
-0.02
-0.03
I
ALDH1A3
SARS-CoV-2
control
trans-ISRIB
Percent
NormMeanExp
0.02
0.01
0
-0.01
-0.02

Figure S7. Related to Figure 7. *Trans*-ISRIB blocks human beta cells transdifferentiation upon SARS-CoV-2 infection.

(A) Representative confocal images of GCG and INS expression in control or 10 μM *trans*-ISRIB treated human islets at 48 hpi (*n* = 3 individual islet donors, MOI=1). Scale bar = 50 μm. Red: INS; Green: GCG; Blue: DAPI.

(B) Representative confocal images of Typsin1 and INS expression in control or 10 μM *trans*-ISRIB treated human islets at 48 hpi (*n* = 3 individual islet donors, MOI=1). Scale bar = 50 μm. Red: INS; Green: Typsin1; Blue: DAPI.

(C and D) Representative confocal images of INS and OPP (C) and quantification of OPP staining intensity (D) of control or 10 μM *trans*-ISRIB treated human islets at 48 hpi (*n* = 3 replicates, MOI=1). Scale bar = 50 μm. Red: OPP; Green: INS; Blue: DAPI.

(E) Representative confocal images of G3BP1 and INS expression in control or 10 μM *trans*-ISRIB treated human islets at 48 hpi (*n* = 3 individual islet donors, MOI=1). Scale bar = 50 μm. Red: INS; Green: G3BP1; Blue: DAPI.

(F and G) Representative confocal images of G3BP1 and INS (F) and quantification of G3BP1 expression (G) in control, 0.5 mM $NaAsO_2$ and 10 μM *trans*-ISRIB+ 0.5 mM $NaAsO_2$ treated human islets after 30 min treatment (*n* = 3 replicates). Scale bar = 50 μm. Green: INS; Red: G3BP1; Blue: DAPI.

(H) Dot blot illustrating expression level of *ALDH1A3* in mock versus SARS-CoV-2 infected human islets at 24 hpi (*n* = 2 individual islet donors, MOI=1).

(I) Dot blot illustrating expression level of *ALDH1A3* in control or 10 μM *trans*-ISRIB treated human islets at 24 hpi (*n* = 2 individual islet donors, MOI=1).

Dot size shows the fraction of cells with non-zero expression; Dot color indicates the relative expression level in the two conditions.

Data was presented as mean ± STDEV. *P* values were calculated by paired or unpaired two-tailed Student's t test. $^{**}P < 0.01$ and $^{****}P < 0.0001$.

Table S1. Related to Figure 1. Summary of samples.

Sample ID	Gender	Age	Assays	Chronic comorbiditi es	Inflammat ory status WBC	COVID-19 disease course	Blood glucose level (mg/dL)
COVID-19_1	Female	87	Immunostaining	DM2, HTN	17.4	7	248
COVID-19_2	Female	70	Immunostaining	Dementia, DM2	4.9	12	223
COVID-19_3	Male	87	Immunostaining	None	9.7	26	213
COVID-19_4	Male	91	Immunostaining	Dementia, HTN	6.8	5	229
COVID-19_5	Female	71	Immunostaining	Alzheimer's, HTN, AFIB	4.2	7	100
Non-COVID-19_1	Male	75	Immunostaining	HTN, CAD	18.9	N/A	201
Non-COVID-19_2	Female	74	Immunostaining	CAD, mild HTN	9.2	N/A	190
Non-COVID-19_3	Male	19	Immunostaining	Asthma	20.4	N/A	150
Non-COVID-19_4	Male	27	Immunostaining	Ashama, HTN, OB	21.6	N/A	137
Non-COVID-19_5	Male	27	SARS-CoV-2 infection, scRNA-seq and immunostaining	Allergic rhinitis, pharyngitis	7.7	N/A	129
Non-COVID-19_6	Female	58	SARS-CoV-2 infection, scRNA-seq and immunostaining, ELISA	HTN, Stroke	12.4	N/A	144
Non-COVID-19_7	Male	52	SARS-CoV-2 infection and immunostaining, ELISA	HTN	7.1	N/A	104
Non-COVID-19_8	Male	18	SARS-CoV-2 infection, immunostaining, ELISA	N/A	N/A	N/A	N/A
Non-COVID-19_15	Male	50	scRNA-seq (Table S3)	N/A	N/A	N/A	N/A
Non-COVID-19_16	Male	28	scRNA-seq (Table S3)	N/A	N/A	N/A	N/A
Non-COVID-19_17	Male	35	scRNA-seq (Table S3)	N/A	N/A	N/A	N/A

Non-COVID-19_18	Male	22	scRNA-seq (Table S3)	N/A	N/A	N/A	N/A
Non-COVID-19_19	Female	41	scRNA-seq (Table S3)	N/A	N/A	N/A	N/A
Non-COVID-19 (T2D)_20	Male	61	scRNA-seq (Table S3)	N/A	N/A	N/A	N/A

DM2: diabetes mellitus, type 2; HTN: Hypertension; AFIB: Atrial fibrillation; CAD: Coronary Artery Disease; OB: obesity.

Table S2. Related to Figure 1. The SARS-N$^+$ cells in autopsy samples*.

Sample ID	COVID-19_1	COVID-19_2	COVID-19_3	COVID-19_4	COVID-19_5
# of SARS-N$^+$INS$^+$ cells	6.5±2.0	9.7±4.7	14.5±4.8	8.0±7.0	23.6±7.5
% of SARS-N$^+$INS$^+$ cells in INS$^+$ cells	10.0±10.9%	15.7±15.3%	7.7±4.1%	8.1±5.9%	12.0±9.1%
# of SARS-N$^+$ KRT19$^+$ cells	22.0±18.5	4.9±5.8	8.3±8.5	3.3±3.2	8.0±13.9
% of SARS-N$^+$ KRT19$^+$ cells in KRT19$^+$ cells	9.0±6.8%	2.1±2.4%	7.8±6.6%	1.9±1.4%	3.0±5.1%
# of SARS-N$^+$ CD31$^+$ cells	29.3±6.0	34.1±22.6	3.0±3.0	9.3±3.5	1.6±1.5
% of SARS-N$^+$ CD31$^+$ cells in CD31$^+$ cells	21.0±0.9%	27.4±13.1%	4.1±4.8%	11.8±4.2%	2.3±2.3%
# of SARS-N$^+$ VIM$^+$ cells	84.7±68.7	5.3±1.5	10.4±4.8	4.2±2.4	7.7±4.0
% of SARS-N$^+$ VIM$^+$cells in VIM $^+$ cells	16.3±10.8%	2.4±1.5%	2.6±1.0%	4.1±1.9%	4.1±0.4%
# of SARS-N$^+$ Trypsin1$^+$ cells	133.7±111.4	4.0±1.0	21.6±15.9	18.2±4.4	5.7±4.5
% of SARS-N$^+$ Trypsin1$^+$ cells in Trypsin1$^+$ cells	15.4±11.0%	0.8±0.0%	2.9±2.1%	2.2±0.5%	1.2±1.0%

*The staining was only performed using autopsy samples that contain viral transcripts as confirmed by qRT-PCR.

Table S3. Related to Figure 2. The percentage of *FURIN*$^+$ or *CTSL*$^+$ cells in six human islet samples.

	**Percentage of *FURIN*$^+$ cells** (Mean±STDEV)	**Percentage of *CTSL*$^+$ cells** (Mean±STDEV)
acinar cells	27.3±17.5	25.9±19.0
alpha cells	23.5±13.8	51.5±22.9
beta cells	31.1±19.0	51.4±30.2
delta cells	33.1±12.1	62.7±22.7
PP cells	36.6±26.0	48.5±28.7
ductal cells	42.8±17.9	51.3±24.6
fibroblasts	47.2±19.4	76.1±18.5
endothelial cells	32.8±32.8	47.3±28.6
immune cells	29.9±17.8	90.5±11.5

Table S4. Related to STAR Methods. Antibodies used for immunocytochemistry, intracellular flow cytometry analysis.

Usage	Antibody	Clone#	Host	Catalog #	Vendor	Dilution
Immuno-staining	Polyclonal Guinea Pig Anti-Insulin	Polyclonal	Guinea Pig	#A0564	Dako	1:500
Immuno-staining	Anti-Glucagon antibody	Monoclonal	Mouse	#ab10988	Abcam	1:1,000
Immuno-staining	Human/Mouse Somatostatin Antibody	Monoclonal	Rat	#MAB2358	R&D Systems	1:100
Immuno-staining	Pancreatic Polypeptide/PP Antibody	Polyclonal	Goat	#NB100-1793	NOVUS Biologicals	1:200
Immuno-staining	Human Serpin A1/ alpha 1-Antitrypsin Antibody	Monoclonal	Mouse	#MAB1268	R&D Systems	1:200
Immuno-staining	Trypsin1/PRSS1 Antibody	Polyclonal	Sheep	#AF3848	NOVUS Biologicals	1:2,500
Immuno-staining	Anti-Cytokeratin 19 antibody	Monoclonal	Mouse	# ab7754	Abcam	1:200
Immuno-staining	Human CD31/PECAM-1 Antibody	Polyclonal	Sheep	#AF806	R&D Systems	1:200
Immuno-staining	Vimentin antibody	Monoclonal	Mouse	#ab8978	Abcam	1:200
Immuno-staining	NRP1 antibody	Monoclonal	Rabbit	#ab81321	Abcam	1:200
Immuno-staining	NRP1 antibody	Monoclonal	Rabbit	#ST05-30	NOVUS Biologicals	1:200
Immuno-staining	G3BP1 Antibody	Polyclonal	Rabbit	#17798	Cell Signaling Technology	1:500
Immuno-staining	SARS-CoV/SARS-CoV-2 Nucleocapsid Antibody	Monoclonal	Rabbit	#40143-R001	Sino Biological	1:500
Immuno-staining	Human ACE-2 Antibody	Polyclonal	Goat	#AF933	R & D Systems	1:400
Immuno-staining	Alexa Fluor 488 AffiniPure Donkey Anti-Guinea Pig IgG (H+L)	Polyclonal	Donkey	#706-545-148	Jackson ImmunoResearch Labs	1:1,000
Immuno-staining	Donkey anti-Mouse IgG (H+L) Highly Cross-Adsorbed Secondary Antibody, Alexa Fluor 488	Polyclonal	Donkey	#A-21202	Thermo Fisher Scientific	1:1,000

Immuno-staining	Donkey anti-Mouse IgG (H+L) Highly Cross-Adsorbed Secondary Antibody, Alexa Fluor 594	Polyclonal	Donkey	#A-21203	Thermo Fisher Scientific	1:1,000
Immuno-staining	Donkey anti-Mouse IgG (H+L) Highly Cross-Adsorbed Secondary Antibody, Alexa Fluor 647	Polyclonal	Donkey	#A-31571	Thermo Fisher Scientific	1:1,000
Immuno-staining	Donkey anti-Rabbit IgG (H+L) Secondary Antibody, Alexa Fluor 594 conjugate	Polyclonal	Donkey	#A-21207	Thermo Fisher Scientific	1:1,000
Immuno-staining	Donkey anti-Rabbit IgG (H+L) Secondary Antibody, Alexa Fluor 647 conjugate	Polyclonal	Donkey	#A-31573	Thermo Fisher Scientific	1:1,000
Immuno-staining	Donkey anti-Goat IgG (H+L) Cross-Adsorbed Secondary Antibody, Alexa Fluor 488	Polyclonal	Donkey	#A-11055	Thermo Fisher Scientific	1:1,000
Immuno-staining	Donkey anti-Rat IgG (H+L) Highly Cross-Adsorbed Secondary Antibody, Alexa Fluor 488	Polyclonal	Donkey	#A-21208	Thermo Fisher Scientific	1:1,000
Immuno-staining	Donkey anti-Sheep IgG (H+L) Cross-Adsorbed Secondary Antibody, Alexa Fluor 488	Polyclonal	Donkey	#A-11015	Thermo Fisher Scientific	1:1,000
Flow Cytometry	Polyclonal Guinea Pig Anti-Insulin	Polyclonal	Guinea Pig	#A0564	Dako	1:500
Flow Cytometry	Anti-Glucagon antibody	Monoclonal	Mouse	#ab10988	Abcam	1:1,000
Flow Cytometry	Neuropilin-1 antibody	Polyclonal	Sheep	#AF3870	NOVUS Biologicals	1:200
Flow Cytometry	Alexa Fluor 488 AffiniPure Donkey Anti-Guinea Pig IgG (H+L)	Polyclonal	Donkey	#706-545-148	Jackson ImmunoResearch Labs	1:1,000
Flow Cytometry	Donkey anti-Mouse IgG (H+L) Highly Cross-Adsorbed Secondary Antibody, Alexa Fluor 647	Polyclonal	Donkey	#A-31571	Thermo Fisher Scientific	1:1,000

Flow Cytometry	Goat anti-Rabbit IgG (H+L) Cross-Adsorbed Secondary Antibody, Pacific Blue	Polyclonal	Goat	#P-10994	Thermo Fisher Scientific	1:1,000
Western Blot	eIF2alpha Rabbit mAb	Monoclonal	Rabbit	#5324S	Cell Signaling	1:1,000
Western Blot	Phospho-eIF2alpha (Ser51) Rabbit mAb	Monoclonal	Rabbit	#3398S	Cell Signaling	1:1,000
Western Blot	PKR Rabbit mAb	Monoclonal	Rabbit	#12297S	Cell Signaling	1:1,000
Western Blot	β-Actin Rabbit mAb #8457	Monoclonal	Rabbit	#8457S	Cell Signaling	1:1,000
Western Blot	Recombinant Anti-PKR (phospho T446) antibody	Monoclonal	Rabbit	# ab32036	Abcam	1:1,000
Western Blot	IRDye® 800CW Donkey anti-Rabbit IgG Secondary Antibody	Polyclonal	Donkey	#926-32213	LI-COR	1:15,000

Table S5. Related to STAR Methods. Primers used for qRT-PCR.

Primer name	Sequence
ACTB-human-F	*CGTCACCAACTGGGACGACA*
ACTB- human-R	*CTTCTCGCGGTTGGCCTTGG*
SARS-CoV-2-TRS-F	*CTCTTGTAGATCTGTTCTCTAAACGAAC*
SARS-CoV-2-TRS-R	*GGTCCACCAAACGTAATGCG*
SARS-CoV-2-N-R	*TAATCAGACAAGGAACTGATTA*
SARS-CoV-2-N-R	*CGAAGGTGTGACTTCCATG*
SARS-CoV-2-E-F	*ACAGGTACGTTAATAGTTAATAGCGT*
SARS-CoV-2-E-R	*ATATTGCAGCAGTACGCACACA*
18S-F	*GGCCCTGTAATTGGAATGAGTC*
18S-R	*CCAAGATCCAACTACGAGCTT*
